# Design, synthesis, anticancer activity, and mechanistic investigation of 4,5,6,7-tetrahydrobenzo[*b*]thiophene carboxamides as CDK-2 inhibitors: *in vitro* and *in silico* DFT and molecular docking study

**DOI:** 10.1080/14756366.2026.2656826

**Published:** 2026-05-29

**Authors:** Kurls E. Anwer, Ramadan M. Ramadan, Eman S. Nossier, Najla A. Altwaijry, Asmaa Saleh, Stefan Bräse, Ebtehal M. Husseiny

**Affiliations:** ^a^ Chemistry Department, Faculty of Science, Ain Shams University, Cairo, Egypt; ^b^ Pharmaceutical Medicinal Chemistry and Drug Design Department, Faculty of Pharmacy (Girls), Al-Azhar University, Cairo, Egypt; ^c^ Department of Pharmaceutical Sciences, College of Pharmacy, Princess Nourah bint Abdulrahman University, Riyadh, Saudi Arabia; ^d^ Institute of Biological and Chemical Systems, IBCS-FMS, Karlsruhe Institute of Technology, Karlsruhe, Germany; ^e^ Pharmaceutical Organic Chemistry Department, Faculty of Pharmacy (Girls), Al-Azhar University, Cairo, Egypt

**Keywords:** Anticancer, CDK-2, DFT, synthesis, tetrahydrobenzo[*b*]thiophene

## Abstract

Utilising drug design methodologies including bioisosteric modification and substituents variation, sets of 4,5,6,7-tetrahydrobenzo[*b*]thiophene carboxamides were synthesised, by conventional heating and eco-friendly microwave-assisted techniques, as CDK-2 inhibitors. These entities were assessed for their antitumor effects against hepatic HepG-2 and breast MCF-7 and MDA-MB-231 carcinomas, in which dimethoxy **5** and dimethyl-bearing analogues **6** and **11** demonstrated significant cytotoxicity and selectivity against the examined cancer cells. Consequently, they were chosen for further assays to determine their mechanism. The findings suggest that these compounds may exert cytotoxicity by inhibiting CDK-2. Compound **11** displayed the highest CDK-2 inhibition, exceeding roscovitine by nearly threefold. Besides, it arrested the MDA-MB-231 cell cycle at the G0/G1 phase by apoptotic stimulation. Molecular modelling showed strong binding of the bioactive analogues to the active pocket of CDK-2 receptor, suggesting their potential as lead inhibitors.

## GRAPHICAL ABSTRACT

**Figure:**
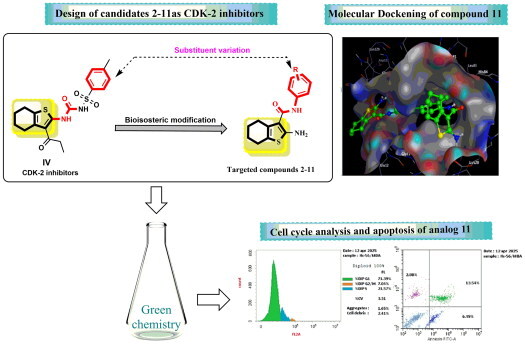

## Introduction

Cancer is the primary cause of mortality globally^1^. Despite substantial advancements in cancer therapy, certain restrictions persist. These drawbacks include side effects, non-selectivity for cancer cells, and the emergence of numerous drug-resistant carcinomas^2^. Therefore, considerable effort is being made to inhibit tumour growth by employing newly prepared compounds^3^. To design and develop safer, more selective anticancer medications, it is critical to understand the mechanisms that control the cell cycle^4^.

Cyclin-dependent kinases (CDKs) are enzymes that are responsible for transferring a phosphate moiety from adenosine triphosphate to proteins with serine/threonine residues^5^. Human cells contain many types of CDKs, which are divided into transcription-associated and cell-cycle-associated categories^6^. CDKs bind to cyclins and play a critical role in regulating cell cycle progression, transcription, and apoptosis^7^. Hence, CDKs are considered important targets in cancer therapy due to their roles in cell cycle regulation and transcription, as well as their overexpression in numerous cancer types^8^^,^^9^. The overexpression of CDK-2 was noted in various tumours, including breast^10^, liver^11^, kidney^12^, ovary^13^, prostate^14^, and colon cancers^15^. Consequently, CDK-2 has attracted close attention recently as a crucial target in drug discovery^16^.

**Figure 1.**
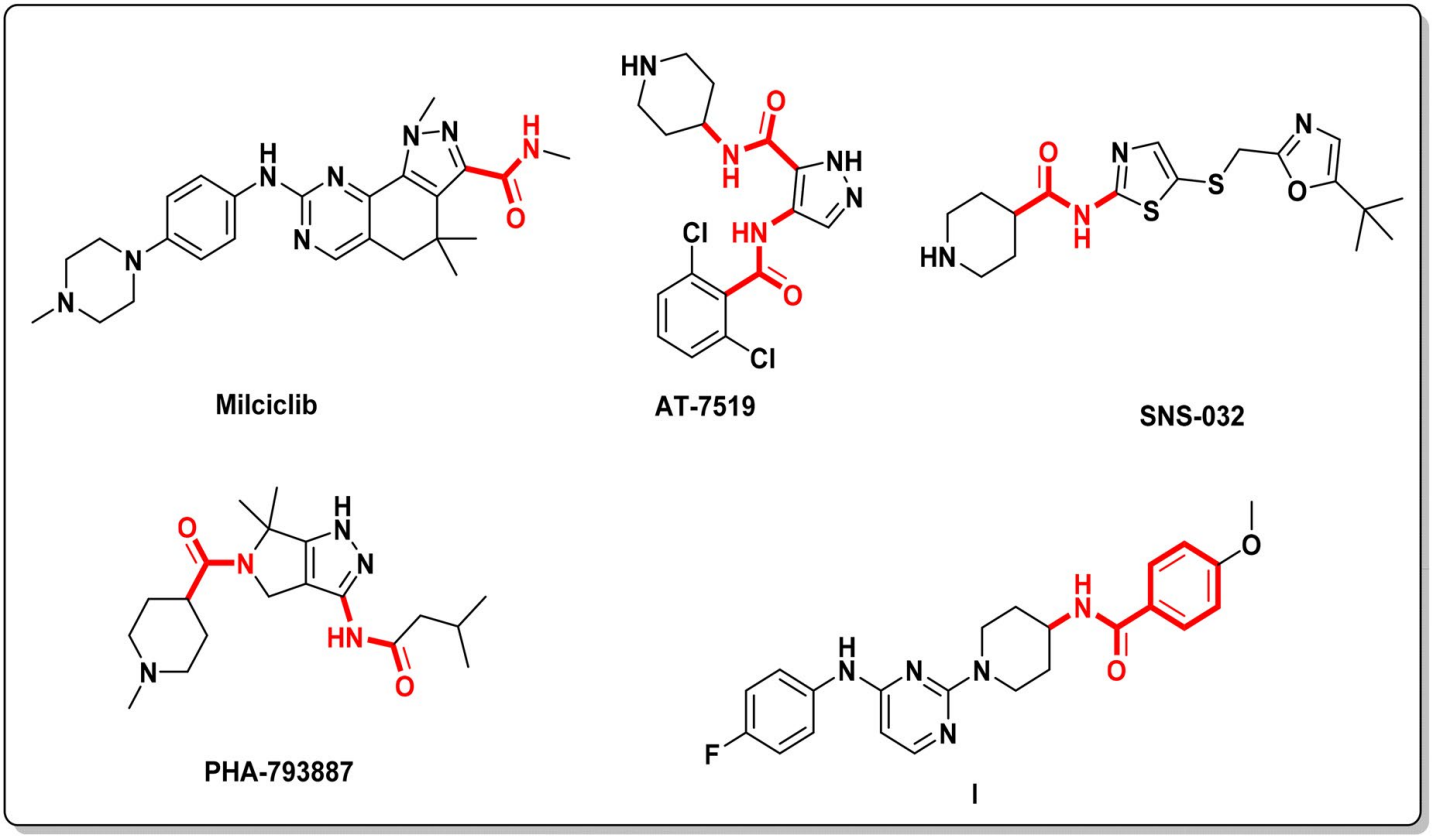
Carboxamide-containing compounds as potent CDK-2 inhibitors.

Carboxamide-containing heterocycles have several biological applications in pharmaceutical chemistry, including anticancer^17^, anti-inflammatory^18^, antimicrobial^19^, antiviral^20^, analgesic^21^, and antimalarial^22^ effects. A literature survey showed that carboxamide-containing heterocycles play a crucial role as antitumor agents^8^. Milciclib is a promising selective ATP-competitive CDK-2 inhibitor (IC_50_ = 45 nM) that entered phase 2 clinical trials for the treatment of hepatocellular carcinoma. AT7519 is a pan-multi-CDK inhibitor 1, 2, 4, 6, and 9 (IC_50_ range = 10–210 nM) that entered phase 2 clinical trials for the management of chronic lymphocytic leukaemia. Also, SNS-032 is a CDK-2 inhibitor (IC_50_ = 48 nM) used in the treatment of chronic lymphocytic leukaemia, but it is currently in a phase 1 clinical trial. PHA-793887 is a potent, selective CDK-2 inhibitor (IC_50_ = 8 nM) in phase 1 clinical trials for the treatment of metastatic solid tumours^8^. It was reported that pyrimidine carboxamide analogue **I** exhibited promising antitumor activity against MDA-MB-468 through CDK-2 inhibition, apoptosis induction, and cell cycle arrest. The importance of the carboxamide moiety in compound **I** was confirmed by its molecular docking, which illustrated a hydrogen bond (HB) formation with Gln131 residue (Figure 1)^17^.

**Figure 2.**
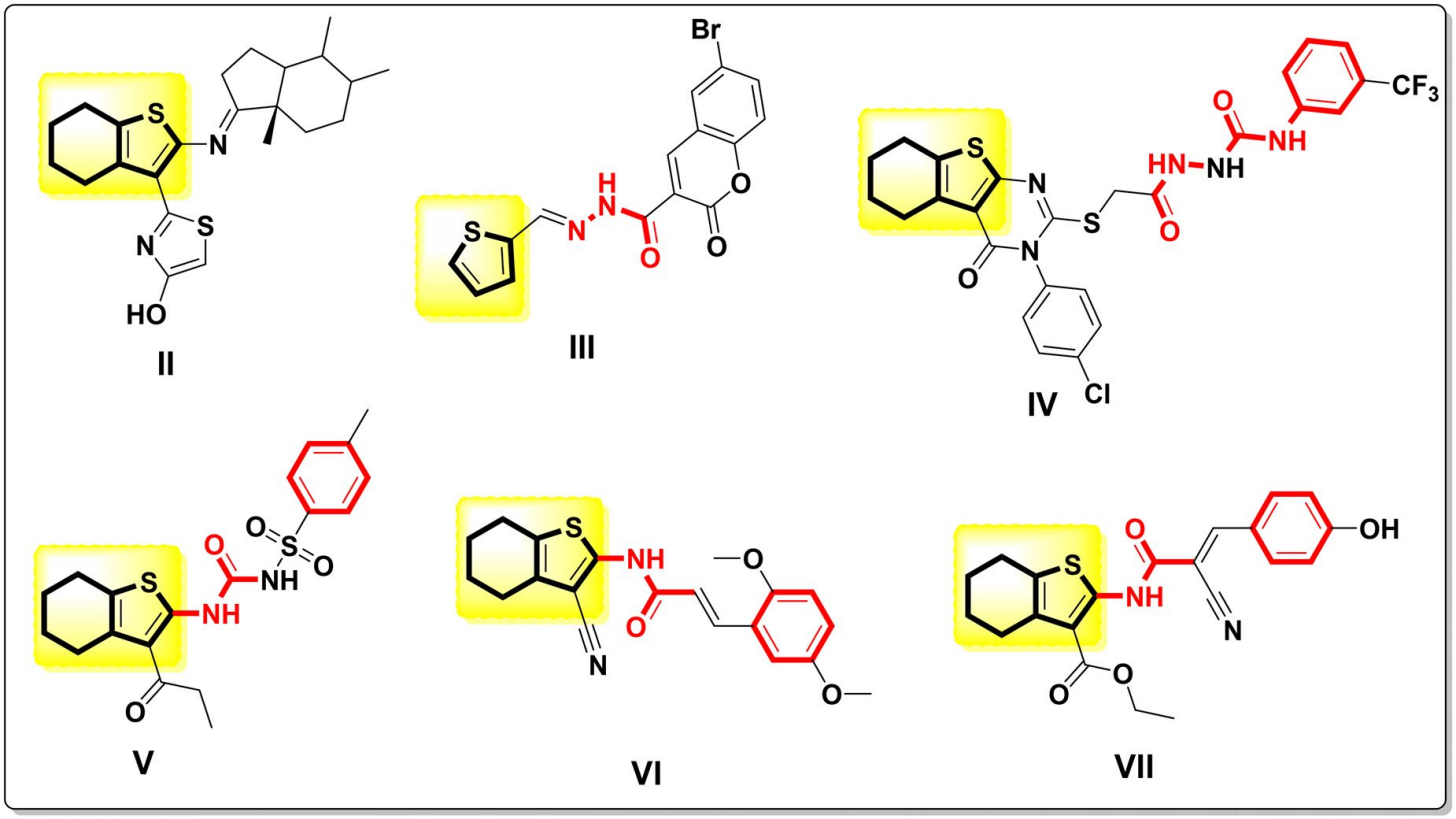
Reported thiophene and tetrahydrobenzo[*b*]thiophene as promising anticancer agents and CDK-2 inhibitors.

Thiophenes and their fused hybrids have attracted the attention of numerous chemists owing to their fascinating biological activities, including antitumor^23^^,^^24^, antimicrobial^25^, anti-influenza^26^, anti-inflammatory^27^, and antioxidant^23^ effects. Tetrahydrobenzo[*b*]thienothiazoloandrostane **II** exhibited promising cytotoxicity against HCT-116, HepG-2, A-549, and MDA-MB-231 cells, with an IC_50_ in the low micromolar range, via CDK-2 inhibition and apoptosis stimulation^18^. Thiophene carbohydrazide **III** displayed significant anticancer activity against CCRF, Panc-1, and HepG-2 cells by elevating CDKN1A, GDF-15, and DDIT4 and decreasing CDC-20, CDC-2, and CDK-2^19^^,^^20^. Furthermore, the tetrahydrobenzothienopyrimidine-containing hydrazine-1-carboxamide tail **IV** exhibited broad-spectrum anticancer activity against multiple NCI carcinomas and arrested the MCF-7 cell cycle^21^^,^^22^. Tetrahydrobenzo[*b*]thiophene carbamoyl analogue **V** showed a potent antitumor effect against HepG-2 cells, with an IC_50_ in the low micromolar range, through CDK-2 inhibition and increased DNA fragmentation^28^. Besides, tetrahydrobenzo[*b*]thiophene acrylamide **VI** demonstrated potent cytotoxic and selective activity against the examined carcinomas, including HepG-2, MCF-7, and PC-3, with an IC_50_ exceeding that of 5-fluorouracil by more than twofold^23^. Additionally, acrylamidotetrahydrobenzo[*b*]thiophene-3-carboxylate **VII** showed very potent inhibitory activity against HepG-2, MCF-7, and A-549 that may be assigned to the presence of the tetrahydro[*b*]benzothiophene-3-carboxylate scaffold (Figure 2).^24^

**Figure 3.**
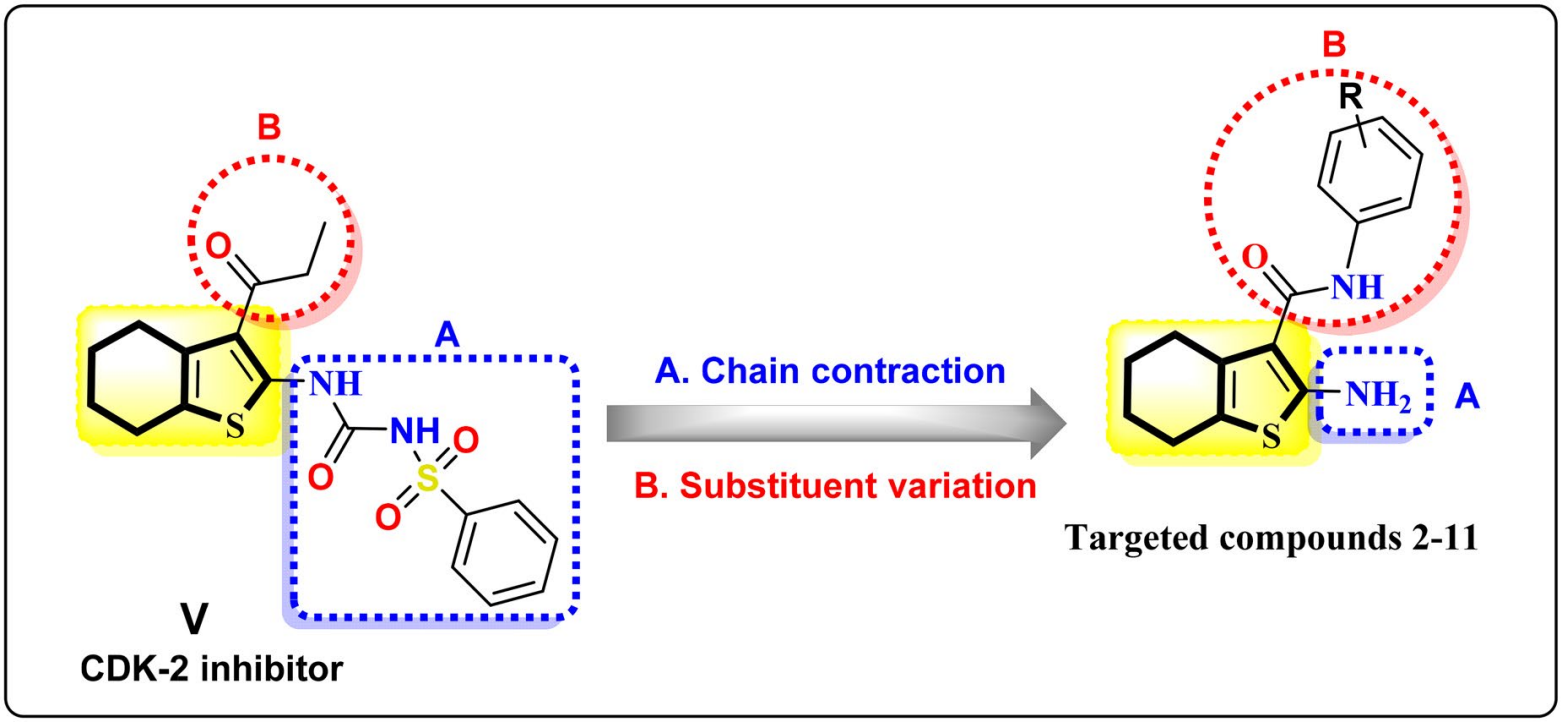
Design of tetrahydrobenzo[*b*]thiophene carboxamide candidates **2**–**11**.

Guided by the previous data, new sets of tetrahydrobenzo[*b*]thiophene carboxamides **2–11** were designed using tetrahydrobenzo[*b*]thiophene carbamoyl analogue **V** by keeping the tetrahydrobenzo[*b*]thiophene scaffold and bioisosteric modifications that include chain contraction at C-2 position and substituent variations at C-3 position, to get new anticancer agents with CDK-2 inhibitory activity (Figure 3). All the prepared analogues were estimated against HepG-2, breast MCF-7, and MDA-MB-231 cells. The superior analogues were selected for the CDK-2 inhibition assay. The potent derivative was also assessed in apoptosis and cell cycle analysis. Also, DFT and molecular docking were used to identify a valid approach to optimising anticancer medications.

## Materials and methods

### Conventional heating procedures

The tools used to characterise the synthesised analogues are provided in the supplementary materials. Compound **1** was synthesised, and its X-ray crystallography was reported earlier^25^.

#### General procedure for the synthesis of 4,5,6,7-tetrahydrobenzo[b]thiophenes (2–6)

A solution of entity **1** (2.25 g, 0.01 mol) in acetone (15 ml) was refluxed for 4–16 h with each of *p*-phenylenediamine (1.08 g, 0.01 mol), *m*-phenylenediamine (1.08 g, 0.01 mol), benzidine (1.84 g, 0.01 mol), *o*-dianisidine (2.44 g, 0.01 mol), and 3,3′-dimethylbenzidine (2.12 g, 0.01 mol). Upon cooling, the solid was filtered, washed three times with ethanol (50 ml), and recrystallised from a suitable solvent to yield analogues **2–6**, respectively.

##### 2-Amino-N-(4-aminophenyl)-4,5,6,7-tetrahydrobenzo[b]thiophene-3-carboxamide (2)

Grey crystals; recrystallised from methanol, m.p.: 182–184 °C. IR (KBr) *υ* cm^−1^: 3402, 3297, 3231 (NH_2_), 3168 (NH), 1646 (C═O), 1596, 1575 (C═C). ^1^H NMR (DMSO-*d*_6_) *δ* (ppm): 1.60–1.77 (m, 4H, tetrahydrobenzothiophene-C_5,6_-CH_2_), 2.39–2.45 (m, 2H, tetrahydrobenzothiophene-C_4_-CH_2_), 2.59–2.65 (m, 2H, tetrahydrobenzothiophene-C_7_-CH_2_), 6.13 (s, 2H, NH_2_, D_2_O exchangeable), 7.23 (d, 2H, *J* = 8 Hz, C_6_H_4_-C_3,5_-H), 7.68 (d, 2H, *J* = 8 Hz, C_6_H_4_-C_2,6_-H), 8.32 (s, 2H, NH_2_, D_2_O exchangeable), 9.51 (s, 1H, NH, D_2_O exchangeable). ^13^C NMR (DMSO-*d*_6_) *δ* (ppm): 22.9, 23.3, 24.1, 24.4, 115.8, 121.7, 121.8, 122.1, 125.5, 128.7, 131.6, 137.7, 149.1, 149.6, 163.2. MS: 287 *m/z* (29.24%). Anal. Calcd for C_15_H_17_N_3_OS (287): C, 62.69; H, 5.96; N, 14.62; S, 11.16. Found: C, 62.68; H, 6.01; N, 14.59; S, 11.23.

##### 2-Amino-N-(3-aminophenyl)-4,5,6,7-tetrahydrobenzo[b]thiophene-3-carboxamide (3)

Black crystals; recrystallised from acetone, m.p.: 156–158 °C. IR (KBr) *υ* cm^−1^: 3402, 3296 (NH_2_), 3168 (NH), 1682 (C═O), 1640, 1595, 1575 (C═C). ^1^H NMR (DMSO-*d*_6_) *δ* (ppm): 1.55–1.73 (m, 4H, tetrahydrobenzothiophene-C_5,6_-CH_2_), 2.40–2.49 (m, 2H, tetrahydrobenzothiophene-C_4_-CH_2_), 2.55–2.68 (m, 2H, tetrahydrobenzothiophene-C_7_-CH_2_), 5.20 (s, 2H, NH_2_, D_2_O exchangeable), 6.52–6.70 (m, 2H, C_6_H_4_-C_2,4_-H), 7.17–7.40 (m, 2H, C_6_H_4_-C_5,6_-H), 8.32 (s, 2H, NH_2_, D_2_O exchangeable), 10.20 (s, 1H, NH_,_ D_2_O exchangeable). ^13^C NMR (DMSO-*d*_6_) *δ* (ppm): 22.9, 23.3, 24.4, 27.0, 115.8 (2C), 117.1, 121.2, 125.4, 128.7, 131.6, 141.7, 156.0, 162.8, 165.9. MS: 287 *m/z* (9.98%). Anal. Calcd for C_15_H_17_N_3_OS (287): C, 62.69; H, 5.96; N, 14.62; S, 11.16. Found: C, 62.79; H, 5.87; N, 14.71; S, 11.09.

##### 2-Amino-N-(4′-amino-[1,1′-biphenyl]-4-yl)-4,5,6,7-tetrahydrobenzo[b]thiophene-3-carboxamide (4)

Brown crystals; recrystallised from ethanol, m.p.: 142–144 °C. IR (KBr) *υ* cm^−1^: 3402, 3296, 3225 (NH_2_), 3168 (NH), 1702 (C═O), 1646, 1596, 1575 (C═C). ^1^H NMR (DMSO-*d*_6_) *δ* (ppm): 1.55–1.80 (m, 4H, tetrahydrobenzothiophene-C_5,6_-CH_2_), 2.70–2.80 (m, 2H, tetrahydrobenzothiophene-C_4_-CH_2_), 2.85–2.95 (m, 2H, tetrahydrobenzothiophene-C_7_-CH_2_), 5.01 (s, 2H, NH_2_, D_2_O exchangeable), 7.21–7.70 (m, 2H, C_6_H_4_-C_3′,5′_-H), 6.59–6.66 (m, 6H, C_6_H_4_-C_2′,6′_-H & C_6_H_4_-C_2,3,5,6_-H), 7.97 (s, 2H, NH_2_, D_2_O exchangeable), 8.01 (s, 1H, NH, D_2_O exchangeable). ^13^C NMR (DMSO-*d*_6_) *δ* (ppm): 22.9, 23.3, 24.4, 27.0, 114.8, 115.9, 120.0 (2C), 127.1, 127.3, 127.4, 127.5, 131.8, 136.6, 147.3, 148.6, 159.9, 162.8, 162.9, 163.4, 165.6. MS: 363 *m/z* (11.31%). Anal. Calcd for C_21_H_21_N_3_OS (363): C, 69.39; H, 5.82; N, 11.56; S, 8.82. Found: C, 69.40; H, 5.84; N, 11.66; S, 8.77.

##### 2-Amino-N-(4′-amino-3,3′-dimethoxy-[1,1′-biphenyl]-4-yl)-4,5,6,7-tetrahydrobenzo[b]thiophene-3-carboxamide (5)

Off-white crystals; recrystallised from methanol, m.p.: 160–162 °C. IR (KBr) *υ* cm^−1^: 3430, 3339, 3296, 3228 (NH_2_), 3167 (NH), 1645 (C═O), 1595, 1574 (C═C). ^1^H NMR (DMSO-*d*_6_) *δ* (ppm): 1.60–1.75 (m, 4H, tetrahydrobenzothiophene-C_5,6_-CH_2_), 2.35–2.45 (m, 2H, tetrahydrobenzothiophene-C_4_-CH_2_), 2.58–2.70 (m, 2H, tetrahydrobenzothiophene-C_7_-CH_2_), 3.85 (s, 3H, OCH_3_), 3.95 (s, 3H, OCH_3_), 4.68 (s, 2H, NH_2_, D_2_O exchangeable), 6.66–7.00 (m, 6H, Ar-H), 7.23 (s, 2H, NH_2_, D_2_O exchangeable), 10.67 (s, 1H, NH, D_2_O exchangeable). ^13^C NMR (DMSO-*d*_6_) *δ* (ppm): 22.9, 23.3, 24.4, 27.0, 55.8, 56.3, 114.5, 115.9, 118.8, 121.0, 121.7, 121.9, 122.1, 125.5, 125.6, 130.2, 131.8, 136.5, 147.0, 147.1, 149.3, 160.3, 163.4. MS: 423 *m/z* (29.17%). Anal. Calcd for C_23_H_25_N_3_O_3_S (423): C, 65.23; H, 5.95; N, 9.92; S, 7.57. Found: C, 65.19; H, 6.02; N, 9.87; S, 7.63.

##### 2-Amino-N-(4′-amino-3,3′-dimethyl-[1,1′-biphenyl]-4-yl)-4,5,6,7-tetrahydrobenzo[b]thiophene-3-carboxamide (6)

Grey crystals; recrystallised from acetone, m.p.: 198–200 °C. IR (KBr) *υ* cm^−1^: 3403, 3375, 3338, 3297 (NH_2_), 3168 (NH), 1645 (C═O), 1626, 1596, 1574 (C═C). ^1^H NMR (DMSO-*d*_6_) *δ* (ppm): 1.65–1.82 (m, 4H, tetrahydrobenzothiophene-C_5,6_-CH_2_), 2.13 (s, 3H, CH_3_), 2.28 (s, 3H, CH_3_), 2.35–2.45 (m, 2H, tetrahydrobenzothiophene-C_4_-CH_2_), 2.55–2.70 (m, 2H, tetrahydrobenzothiophene-C_7_-CH_2_), 4.78 (s, 2H, NH_2_, D_2_O exchangeable), 6.62–7.25 (2m, 6H, Ar-H), 7.77 (s, 2H, NH_2_, D_2_O exchangeable), 10.39 (s, 1H, NH, D_2_O exchangeable). ^13^C NMR (DMSO-*d*_6_) *δ* (ppm): 18.1, 18.6, 22.9, 23.3, 24.4, 27.0, 115.9, 121.7, 121.8, 122.0, 122.1, 123.4, 123.6, 124.1, 124.9, 125.6, 127.7, 127.8, 128.4, 131.7, 145.2, 163.4, 165.6. MS: 391 *m/z* (36.51%). Anal. Calcd for C_23_H_25_N_3_OS (391): C, 70.56; H, 6.44; N, 10.73; S, 8.19. Found: C, 70.63; H, 6.42; N, 10.69; S, 8.18.

#### General method for the synthesis of 4,5,6,7-tetrahydrobenzo[b]thiophenes (7–11)

A solution of entity **1** (4.51 g, 0.02 mol) in acetone (15 ml) was refluxed for 10–28 h with each of *p*-phenylenediamine (1.08 g, 0.01 mol), *m*-phenylenediamine (1.08 g, 0.01 mol), benzidine (1.84 g, 0.01 mol), *o*-dianisidine (2.44 g, 0.01 mol), and 3,3′-dimethylbenzidine (2.12 g, 0.01 mol). Upon cooling, the reaction mixture was poured onto cold H_2_O (50 ml). The resulting mass was filtered and recrystallised from the appropriate solvent to yield compounds **7–11**.

##### N,N′-(1,4-phenylene)bis(2-amino-4,5,6,7-tetrahydrobenzo[b]thiophene-3-carboxamide) (7)

Grey crystals; recrystallised from acetone, m.p.: 232–234 °C. IR (KBr) *υ* cm^−1^: 3324, 3231 (NH_2_), 3184, 3078 (NH), 1730, 1710 (C═O), 1645, 1595, 1575 (C═C). ^1^H NMR (DMSO-*d*_6_) *δ* (ppm): 1.58–1.78 (m, 8H, tetrahydrobenzothiophene-C_5,6_-4CH_2_), 2.37–2.47 (m, 4H, tetrahydrobenzothiophene-C_4_-2CH_2_), 2.55–2.65 (m, 4H, tetrahydrobenzothiophene-C_7_-2CH_2_), 7.23 (s, 4H, Ar-H), 8.51 (s, 4H, 2NH_2_, D_2_O exchangeable), 10.16 (s, 2H, 2NH, D_2_O exchangeable). ^13^C NMR (DMSO-*d*_6_) *δ* (ppm): 22.9 (2C), 23.3 (2C), 24.4 (2C), 27.0 (2C), 115.9 (2C), 122.0 (2C), 125.6 (2C), 129.2 (2C), 131.8 (2C), 137.8 (2C), 163.4 (2C), 165.6 (2C). MS: 466 *m/z* (11.53%). Anal. Calcd for C_24_H_26_N_4_O_2_S_2_ (466): C, 61.78; H, 5.62; N, 12.01; S, 13.74. Found: C, 61.69; H, 5.67; N, 11.95; S, 13.81.

##### N,N′-(1,3-phenylene)bis(2-amino-4,5,6,7-tetrahydrobenzo[b]thiophene-3-carboxamide) (8)

Brown crystals; recrystallised from acetone, m.p.: 266–268 °C. IR (KBr) *υ* cm^−1^: 3315, 3220 (NH_2_), 3172, 3090 (NH), 1735, 1710 (C═O), 1640, 1590, 1574 (C═C). ^1^H NMR (DMSO-*d*_6_) *δ* (ppm): 1.57–1.77 (m, 8H, tetrahydrobenzothiophene-C_5,6_-4CH_2_), 2.32–2.42 (m, 4H, tetrahydrobenzothiophene-C_4_-2CH_2_), 2.54–2.65 (m, 4H, tetrahydrobenzothiophene-C_7_-2CH_2_), 7.21–7.70 (m, 8H, 4 Ar-H and 2NH_2_), 10.14 (s, 1H, NH, D_2_O exchangeable), 10.17 (s, 1H, NH, D_2_O exchangeable). ^13^C NMR (DMSO-*d*_6_) *δ* (ppm): 22.8 (2C), 23.3 (2C), 24.4 (2C), 27.0 (2C), 115.9 (2C), 121.7, 122.1 (2C), 126.8 (2C), 128.8, 137.8 (2C), 144.6 (2C), 159.9 (2C), 160.1 (2C). MS: 466 *m/z* (25.78%). Anal. Calcd for C_24_H_26_N_4_O_2_S_2_ (466): C, 61.78; H, 5.62; N, 12.01; S, 13.74. Found: C, 61.81; H, 5.73; N, 12.12; S, 13.60.

##### N,N′-([1,1′-biphenyl]-4,4′-diyl)bis(2-amino-4,5,6,7-tetrahydrobenzo[b]thiophene-3-carboxamide) (9)

Grey crystals; recrystallised from butanol, m.p.: >300 °C. IR (KBr) *υ* cm^−1^: 3440, 3393, 3350, 3295 (NH_2_), 3227, 3168 (NH), 1755, 1707 (C═O), 1645, 1595, 1575 (C═C). ^1^H NMR (DMSO-*d*_6_) *δ* (ppm): 1.59–1.79 (m, 8H, tetrahydrobenzothiophene-C_5,6_-4CH_2_), 2.38–2.48 (m, 4H, tetrahydrobenzothiophene-C_4_-2CH_2_), 2.56–2.66 (m, 4H, tetrahydrobenzothiophene-C_7_-2CH_2_), 7.23 (s, 4H, 2NH_2_, D_2_O exchangeable), 7.33–7.69 (m, 8H, Ar-H), 11.20 (s, 2H, 2NH, D_2_O exchangeable). ^13^C NMR (DMSO-*d*_6_) *δ* (ppm): 22.9 (2C), 23.3 (2C), 24.4 (2C), 27.0 (2C), 120.0 (4C), 126.4 (2C), 127.3 (2C), 127.7 (4C), 131.8 (2C), 136.6 (2C), 148.6 (2C), 163.4 (2C), 165.6 (2C). MS: 542 *m/z* (22.54%). Anal. Calcd for C_30_H_30_N_4_O_2_S_2_ (542): C, 66.39; H, 5.57; N, 10.32; S, 11.81. Found: C, 66.38; H, 5.62; N, 10.29; S, 11.90.

##### N,N′-(3,3′-dimethoxy-[1,1′-biphenyl]-4,4′-diyl)bis(2-amino-4,5,6,7-tetrahydrobenzo[b]thiophene-3-carboxamide) (10)

Grey crystals; recrystallised from dioxane, m.p.: >300 °C. IR (KBr) *υ* cm^−1^: 3426, 3365, 3297, 3229 (NH_2_), 3168, 3077 (NH), 1674, 1656 (C═O), 1645, 1595, 1574 (C═C). ^1^H NMR (DMSO-*d*_6_) *δ* (ppm): 1.56–1.77 (m, 8H, tetrahydrobenzothiophene-C_5,6_-4CH_2_), 2.36–2.47 (m, 4H, tetrahydrobenzothiophene-C_4_-2CH_2_), 2.55–2.66 (m, 4H, tetrahydrobenzothiophene-C_7_-2CH_2_), 4.13 (s, 6H, 2OCH_3_), 6.74 (s, 4H, 2NH_2_, D_2_O exchangeable), 7.23 (s, 2H, C_6_H_3_-C_2,2′_-H), 7.83–7.90 (m, 4H, C_6_H_3_-C_5,5′, 6, 6′_-H), 9.67 (s, 2H, 2NH, D_2_O exchangeable). ^13^C NMR (DMSO-*d*_6_) *δ* (ppm): 22.9 (2C), 23.3 (2C), 24.4 (2C), 27.0 (2C), 59.6 (2C), 115.9 (2C), 119.7 (2C), 121.9 (2C), 128.2 (2C), 129.1 (2C), 130.4 (2C), 137.8 (2C), 140.6 (2C), 159.9 (2C), 162.8 (2C), 164.9 (2C). MS: 602 *m/z* (18.24%). Anal. Calcd for C_32_H_34_N_4_O_4_S_2_ (602): C, 63.76; H, 5.69; N, 9.30; S, 10.64. Found: C, 63.81; H, 5.49; N, 9.21; S, 10.74.

##### N,N′-(3,3′-dimethyl-[1,1′-biphenyl]-4,4′-diyl)bis(2-amino-4,5,6,7-tetrahydrobenzo[b]thiophene-3-carboxamide) (11)

Off-white crystals; recrystallised from dioxane, m.p.: >300 °C. IR (KBr) *υ* cm^−1^: 3462, 3400, 3347, 3285 (NH_2_), 3230, 3168 (NH), 1683, 1665 (C═O), 1645, 1594, 1575 (C═C). ^1^H NMR (DMSO-*d*_6_) *δ* (ppm): 1.58–1.79 (m, 8H, tetrahydrobenzothiophene-C_5,6_-4CH_2_), 2.14 (s, 6H, 2CH_3_), 2.35–2.46 (m, 4H, tetrahydrobenzothiophene-C_4_-2CH_2_), 2.54–2.66 (m, 4H, tetrahydrobenzothiophene-C_7_-2CH_2_), 6.59–7.70 (m, 10H, Ar-H and 2NH_2_, D_2_O exchangeable), 10.20 (s, 2H, 2NH, D_2_O exchangeable). ^13^C NMR (DMSO-*d*_6_) *δ* (ppm): 18.4 (2C), 22.9 (2C), 23.3 (2C), 24.4 (2C), 27.0 (2C), 115.9 (2C), 123.3 (2C), 124.5 (2C), 126.8 (2C), 131.0 (2C), 131.8 (2C), 134.0 (2C), 144.6 (2C), 146.6 (2C), 163.4 (2C), 165.6 (2C). MS: 570 *m/z* (15.39%). Anal. Calcd for C_32_H_34_N_4_O_2_S_2_ (570): C, 67.34; H, 6.00; N, 9.82; S, 11.23. Found: C, 67.21; H, 6.04; N, 9.84; S, 11.34.

### Microwave-aided protocol

The amounts of reactants and the synthetic procedure of the microwave-aided protocol are similar to the conventional heating technique, but without solvent. Thin-layer chromatography was applied to follow the reaction’s progress. The products were washed with methanol three times and recrystallised with a suitable solvent. A microwave-aided protocol was performed in an Anton Paar Monowave 300 using “10 ml” borosilicate glass vials where the vial was irradiated at 120 °C under 2–5 bar pressure and 200–400 W power for 1.5–5 min with 750 rpm magnetic stirring rate. It was found that the end product of the same reaction was identical in TLC, m.p., and mixed m.p. in both techniques. Compared to the traditional heating method, the microwave method produced higher yields in less time.

### Biological evaluation

#### Cytotoxicity assay

The examined cancer cells, hepatic HepG-2 and breast MCF-7 and MDA-MB-231, have been purchased from American Type Culture Collection (Manassas, VA). MTT technique was applied and the IC_50_ was determined using a non-linear regression model in GraphPad Prism software (San Diego, CA). Doxorubicin and roscovitine were used as standards guided by the reported method^26^.

#### CDK-2 inhibition

CDK-2 kit was obtained from Biosciences (San Diego, CA) and Biovision (Mountain View, CA) and the IC_50_ was detected after repeating the experiments three successive times according to the manufacturer’s guidelines^27^. The detailed procedure was discussed in the supplementary materials.

#### Apoptosis assay

Apoptosis assay using Annexin V-FITC/PI kit was applied by FACS Calibre flow cytometer on dealing with the potent compound **11** at three concentrations (IC_50_ (*x*), 5*x* and 10*x*) following the recorded technique^29^. The detailed procedure was discussed in the supplementary materials.

#### Cell cycle analysis

Analysis of MDA-MB-231 cycle was performed by FACS Calibur flow cytometer (Biosciences, San Jose, CA) on dealing with the potent compound **11** at three concentrations (IC_50_ (*x*), 5*x* and 10*x*) according to the reported procedure^30^. The detailed procedure was discussed in the supplementary materials.

#### In silico studies

##### Molecular orbital computations

The theoretical molecular orbital calculations were carried out utilising (DFT/B3LYP) as in Gaussian 09W software. The energy optimised structures of the synthesised analogues were executed by the standard double zeta plus polarisation with 6–31G (d,p) basis set.

##### Molecular docking analysis

It was performed utilising Molecular Operating Environment (MOE-Dock) software, version 2024.0601. The co-crystallised structure of CDK-2/cyclin A2 with its natural ligand, roscovitine (PDB code: 3DDQ) extracted from the Protein Data Bank was applied.

## Results and discussion

### Chemistry

**Scheme 1.**
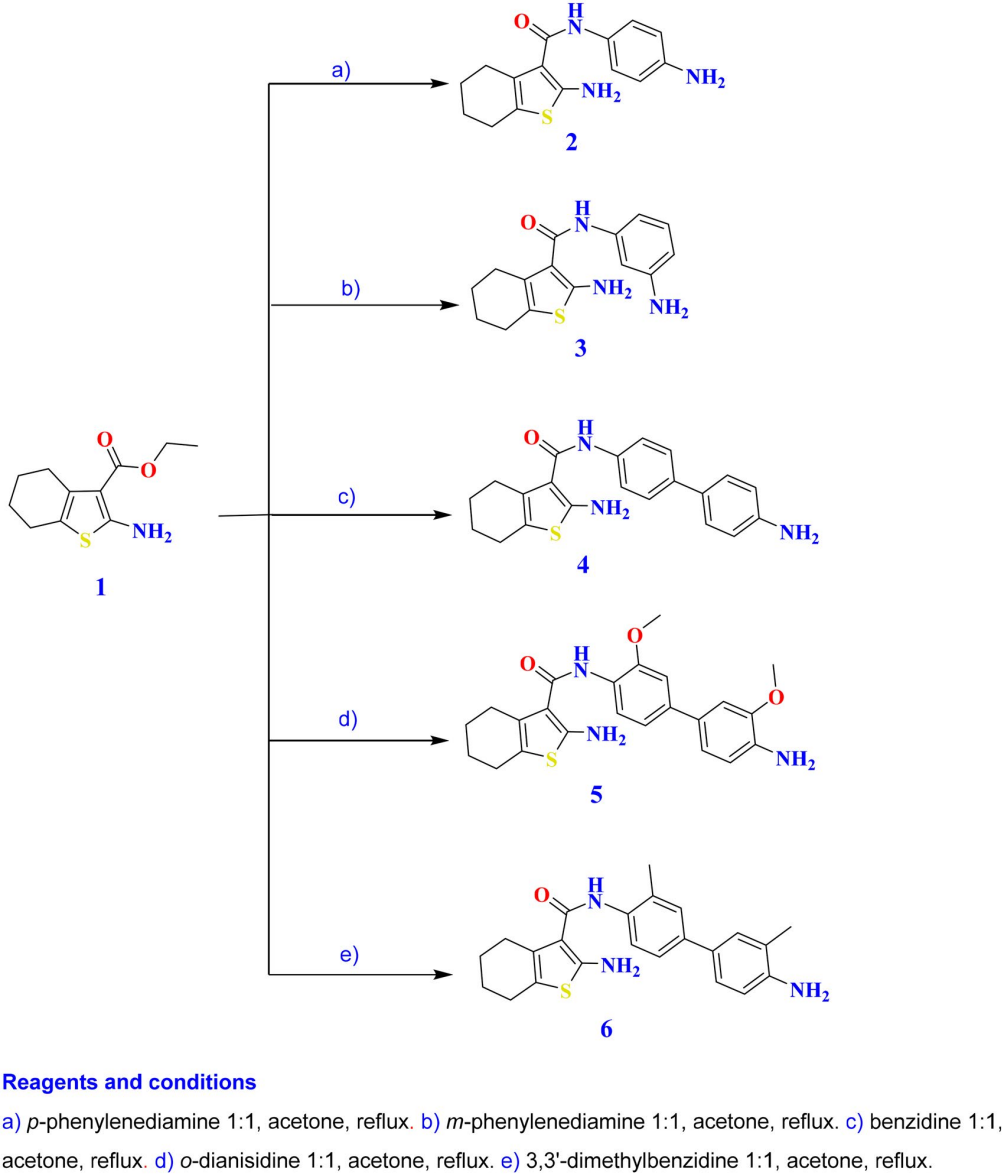
Synthesis of the targeted analogues **2**–**6**.

**Scheme 2.**
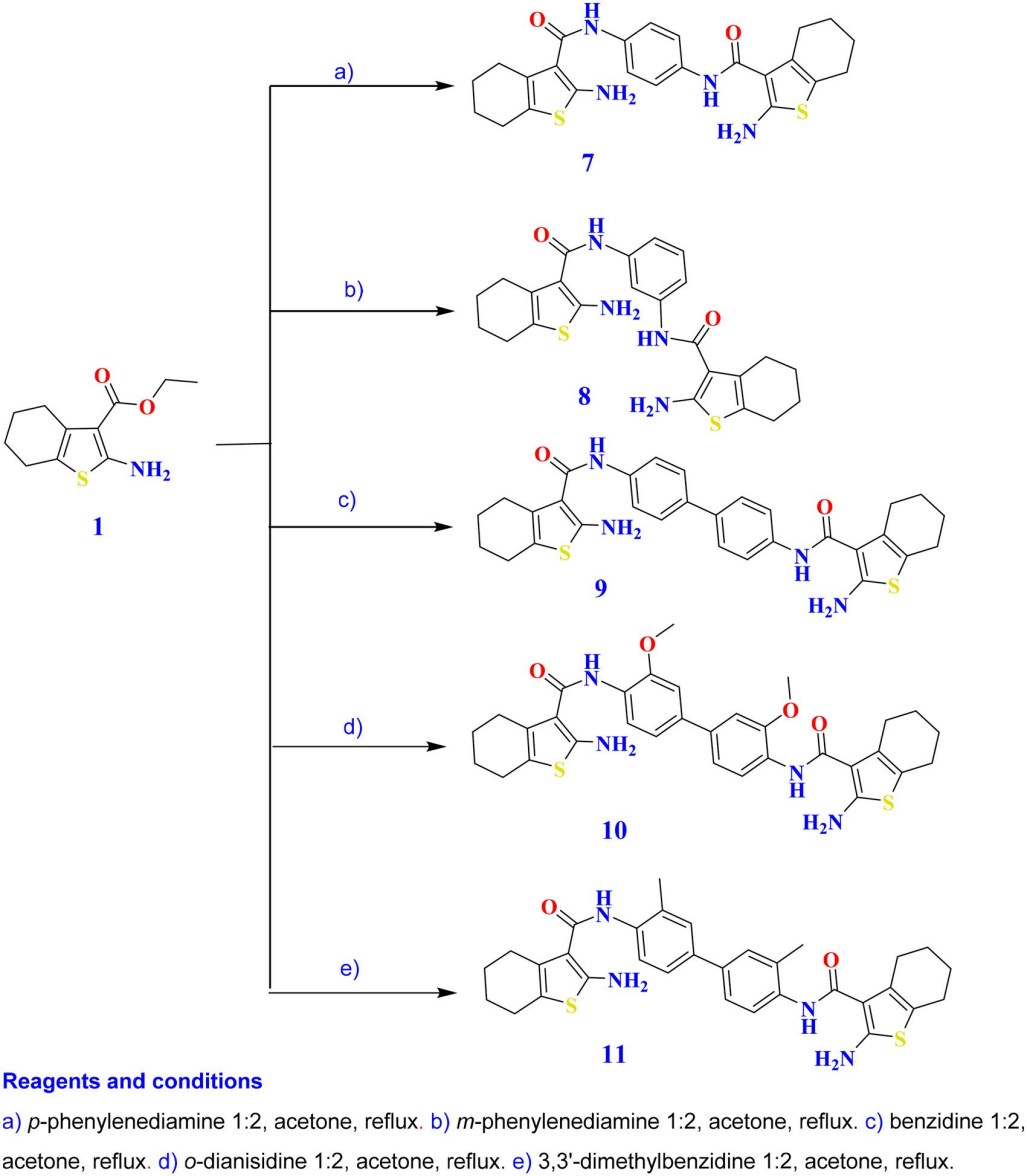
Synthesis of the target compounds **7**–**11**.

The microwave-assisted technique is a significant, eco-friendly approach that has recently been employed in the synthesis of heterocycles^31–33^. The condensation of amines with esters was reported to yield the corresponding amides^32^^,^^34–38^. Hence, unimolecular and bimolecular condensation of ethyl 2-aminobenzo[*b*]thiophene-3-carboxylate **1** with appropriate diamines, including *p*-phenylenediamine, *m*-phenylenediamine, benzidine, *o*-dianisidine, and 3,3′-dimethylbenzidine, furnished the corresponding 4,5,6,7-tetrahydrobenzo[*b*]thiophene-3-carboxamides **2**–**11** as shown in Schemes 1 and 2.

The reactions were intended to proceed via nucleophilic addition of NH_2_ to the ester carbonyl, with the removal of an ethanol moiety to afford carboxamides **2**–**11**. The structures of newly synthesised analogues were evidenced by NH and amidic C═O bands in IR spectra at 3230–3077 and 1755–1645 cm^−1^, respectively. Meanwhile, ^1^H NMR spectra presented the disappearance of triplet and quartette signals connected with the ethyl carboxylate protons in the starting compound. They exhibited two D_2_O exchangeable singlets in the range of 4.68–8.51 and 8.01–11.20 ppm attributed to NH_2_ and NH. Additionally, compounds **5** and **10** presented singlets at 3.85–4.13 ppm corresponding to methoxy protons, while compounds **6** and **11** illustrated singlets at 2.13–2.28 ppm due to methyl protons.

The mentioned spectral data of compounds **2**–**6** were consistent with the proposed mono-carboxamide structures and did not indicate the presence of bis-amide products. High-performance liquid chromatography (HPLC) analysis of compounds **5**, **6**, and **11** emphasised the high purity of the prepared entities. They showed single sharp peaks with retention times ranging from 4.32 to 4.56 min, indicating acceptable chromatographic homogeneity. The purity percent were 98.7, 98.1, and 100 for entities **5**, **6**, and **11**, respectively.

#### Comparison between microwave and conventional thermal methods

A comparison of the newly synthesised compounds prepared by microwave and conventional procedures was conducted with respect to yields and timeframes. Table 1 shows that although the molar concentrations of all reactants were identical in the traditional and microwave techniques, the reaction durations and product yields differed greatly. However, employing both traditional and microwave approaches, the yield economy (YE) was employed to assess synthetically differing efficiencies of the same reaction^39–41^.

$$\text{YE}=\frac{\text{yield\%}}{\text{reaction time }(\text{min})}.$$

**Table 1.** Comparison of reaction efficiencies using conventional heating versus microwave-assisted synthesis.

Cpd. no.	Time (min)		Yield %		YE		OE		RME		AE
	Con.	M.W.	Con.	M.W.	Con.	M.W.	Con.	M.W.	Con.	M.W.	
**2**	240	1.5	42	95	0.1750	63.33	36.213	81.91247	28.63	64.76	79.06
**3**	720	2.5	43	93	0.0597	37.20	37.07311	80.19226	29.31	63.40	79.06
**4**	480	2	41	92	0.0854	46.00	36.21962	81.26738	29.95	67.20	82.69
**5**	960	3	44	95	0.0458	31.67	39.94591	86.26099	29.54	63.79	73.95
**6**	840	4	42	91	0.0500	22.75	37.35818	80.93873	31.28	67.77	83.73
**7**	600	4	42	91	0.0700	22.75	37.55968	81.39257	28.32	61.37	75.40
**8**	900	4	41	92	0.0456	23.00	36.67109	82.28117	27.65	62.04	75.40
**9**	840	4.5	44	93	0.0524	20.67	39.80794	84.14853	31.09	65.72	78.10
**10**	1320	5	45	95	0.0341	19.00	41.35183	87.29221	30.10	63.54	72.79
**11**	1680	5	45	91	0.0268	18.20	40.8613	82.64725	32.26	65.25	78.95

Con.: conventional; M.W.: microwave.

Reaction mass efficiency (RME), on the other hand, is a useful indicator of reaction efficiency and can be calculated using the following formula^42–44^:

$$\text{RME}=\frac{\text{wt of isolated product }}{\text{wt of reactants}}.$$

The two reaction types were directly compared using the optimal efficiency (OE), which may be computed using $\text{OE}=\frac{\text{RME}}{\text{AE}}\times 100$.

AE stands for “atomic economy.” The AE is the same in both procedures used to synthesise the same target product, even though most parameters are larger in the microwave than in the conventional method^45^^,^^46^

### Biological tests

#### In vitro cytotoxicity study

Using the MTT colorimetric method, the compounds’ cytotoxicity was evaluated against three cancer cells, liver HepG-2, breast MCF-7, and MDA-MB-231. Based on their sensitivity to a pharmacophore-bearing tetrahydrobenzo[*b*]thiophene, the target carcinomas were chosen^25^^,^^47–49^. Additionally, the selected cancer cells are found to overexpress CDK-2^50^^,^^51^. Doxorubicin and roscovitine were used as references to assess the anticancer activity of the produced compounds. The findings in Table 2 demonstrate that tetrahydrobenzo[*b*]thiophene-3-carboxamides exhibit mild to extremely potent growth inhibition of the tested cancer cells. Compound **5** demonstrated the most potent cytotoxic effect, particularly against breast cancer cell lines, significantly surpassing doxorubicin. The reliability of these potency estimates was confirmed by the high goodness-of-fit (*R*^2^ ≥ 0.94) and the narrow 95% confidence intervals (CIs) for all tested compounds (detailed statistical parameters are provided in Table S1). The influence of structural variations on biological activity was systematically analysed to develop a clear structure–activity relationship (SAR) profile.

**Table 2.** *In vitro* cytotoxicity.

Analog number	*In vitro* cytotoxicity IC_50_ (µM)*		
	MCF-7	MDA-MB-231	HepG-2
**2**	70.74 ± 3.7 *^**, $$$^	62.41 ± 3.5 ^***, $$$^	82.27 ± 4.1 ^***, $$$^
**3**	47.30 ± 2.8 ^***, $$$^	41.60 ± 2.4 ^***, $$$^	55.77 ± 3.2 ^***, $$$^
**4**	15.26 ± 1.2 ^***, $$^	8.24 ± 0.5	42.19 ± 2.4 ^***, $$$^
**5**	3.59 ± 0.2	2.79 ± 0.1	6.49 ± 0.4
**6**	9.50 ± 0.7	11.93 ± 0.8 ***	8.74 ± 0.6
**7**	29.05 ± 1.9 ^***, $$$^	17.16 ± 1.3 ^***, $$$^	38.04 ± 2.3 ^***, $$$^
**8**	61.41 ± 3.4 ^***, $$$^	58.38 ± 3.3 ^***, $$$^	30.63 ± 2.1 ^***, $$$^
**9**	36.18 ± 2.2 ^***, $$$^	33.01 ± 2.0 ^***, $$$^	64.90 ± 3.5 ^***, $$$^
**10**	22.82 ± 1.5 ^***, $$$^	24.58 ± 1.6 ^***, $$$^	18.28 ± 1.4 ^***, $$^
**11**	6.88 ± 0.5	5.61 ± 0.3	21.51 ± 1.7 ^***, $$$^
**Doxorubicin**	4.17 ± 0.2	3.18 ± 0.1	4.50 ± 0.2
**Roscovitine**	7.26 ± 0.3	7.64 ± 0.4	9.18 ± 0.6

*The findings are the mean of 3 independent biological replicates ± SD. The cancer cells were treated with the prepared analogs at concentrations of 100, 50, 25, 12.5, 6.25, 3.125, and 1.56 µM and incubated for 24 h.

The statistical significance was assessed by one-way ANOVA followed by Tukey post-hoc test. GraphPad InStat (version 3.06) was used for analysis.

***Significantly different from doxorubicin at *p* < 0.001.

$$$ and $$: significantly different from roscovitine at *p* < 0.001 and *p* < 0.01, respectively.

#### Structure–activity relationship

Regarding monocarboxamide analogues **2–6**, compounds **2** and **3**, containing simple 4-aminophenyl and 3-aminophenyl substituents, respectively, demonstrated weak to moderate cytotoxicity (IC_50_ values ranged from 47.3 to 82.27 μM). The slight improvement observed in compound **3** suggests that meta-amino substitution optimises geometric alignment for hydrogen bonding but remains insufficient for high activity. A significant improvement emerged upon introducing biphenyl-based anilide moieties (**4**–**6**). Compound **4**, featuring an unsubstituted biphenyl system, showed a substantial increase in potency (IC_50_ = 8.24 μM on MDA-MB-231), confirming the beneficial role of extended aromatic systems in enhancing π–π stacking and hydrophobic interactions. Electron-donating substituents further modulated activity. The 3,3′-dimethoxy biphenyl analogue **5** displayed the highest potency in the entire series, with IC_50_ values of 3.59 and 2.79 μM against MCF-7 and MDA-MB-231, respectively, surpassing doxorubicin on breast cancer cell lines. Replacing methoxy groups with methyl groups in compound **6** slightly reduced activity but maintained strong potency (IC_50_s ranging from 9.50 to 11.93 μM), indicating that steric and hydrophobic effects contribute positively. However, the hydrogen-bonding properties of the methoxy group confer optimal activity.

**Figure 4.**
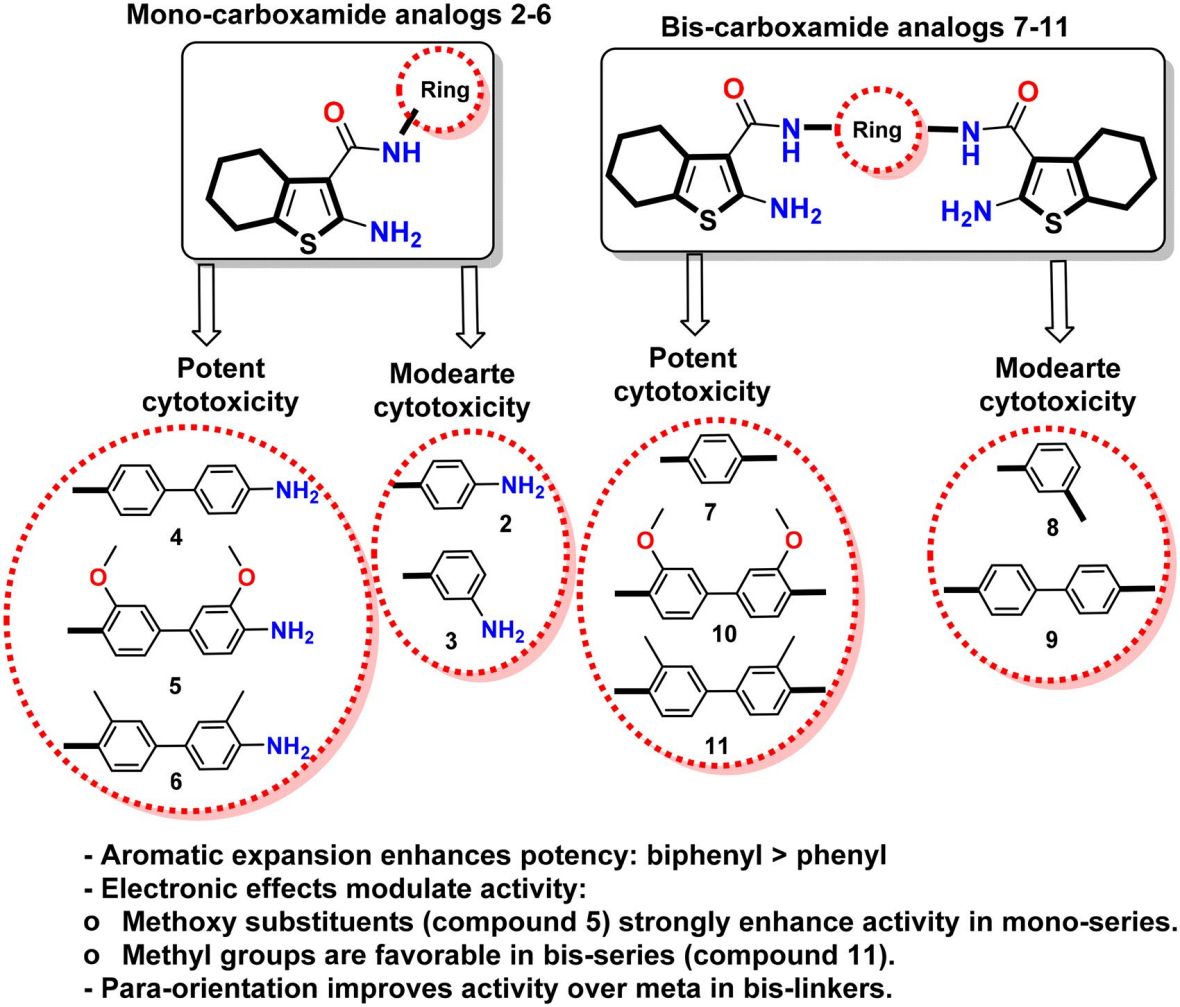
Graphical presentation of SAR.

On the other hand, bis-carboxamide analogues **7**–**11**, compounds with two tetrahydrobenzo[*b*]thiophene pharmacophores linked through aromatic spacers, activity varied depending on spacer geometry and electronic nature. The para-phenylene-linked bis-amide **7** showed moderate activity with IC_50_ values ranging from 17.16 to 38.04 μM, surpassing the meta-phenylene analogue **8**, which had IC_50_ values from 58.38 to 61.41 μM. This implies that a linear para-orientation enhances better molecular alignment and interaction with cellular targets. Introduction of biphenyl spacers **9**–**11** significantly restored biological activity. Among them, compound **11**, possessing 3,3′-dimethyl substituents, revealed the highest potency (IC_50_s ranging from 5.61 to 6.88 μM in breast cancer lines). These results suggest that hydrophobicity and steric reinforcement improve binding affinity within the bis-series. The dimethoxy analogue **10** exhibited moderate potency, weaker than that of **11**, indicating that, in bis-amide structures, increased hydrophobic character is more influential than H-bonding capacity. A graphical presentation of SAR is summarised in Figure 4.

The previous outcomes confirm that biphenyl-based tetrahydrobenzo[*b*]thiophene carboxamides, particularly those bearing electron-rich substituents, represent promising anticancer candidates. Compound **5** is the standout analogue with excellent potency, while compound **11** demonstrates the optimal design for bis-carboxamide derivatives.

#### Calculation of selectivity indices

A compound’s selectivity index (SI) is determined by dividing its IC_50_ against a normal cell by that of a malignant cell^52–55^. To verify the selectivity of these candidates towards cancer cells, the cytotoxicity of the strongest anticancer compounds, **4**, **5**, **6**, and **11**, against WI-38 cells was measured. According to the results, they showed encouraging selectivity for the studied carcinomas (SI range of 1.37–12.71) compared with the references, roscovitine and doxorubicin (SI range of 1.16–2.11). Moreover, the superior cytotoxic analogue **5**, with dimethoxybiphenyltetrahydrobenzo[*b*]thiophene-3-carboxamide, exhibited the greatest selectivity towards MCF-7, MDA-MB-231, and HepG-2 cells, with SI values of 9.87, 12.71, and 5.46, respectively. Also, compound **6**, containing dimethylbiphenyltetrahydrobenzo[*b*]thiophene-3-carboxamide scaffold, displayed significant selectivity towards MCF-7, MDA-MB-231, and HepG-2 carcinomas with SI of 7.50, 5.97, and 8.15, respectively. Additionally, compound **11**, having dimethylbiphenylbis(tetrahydrobenzo[*b*]thiophene-3-carboxamide), exhibited significant selectivity against breast MCF-7 and MDA-MB-231 cells (SI = 7.63 and 9.36, respectively) and moderate selectivity against HepG-2 (SI = 2.44). However, compound **4**, with biphenyltetrahydrobenzo[*b*]thiophene-3-carboxamide, exhibited potent selectivity against MDA-MB-231 (SI = 7.02) and modest selectivity towards MCF-7 (SI = 3.79) with no selectivity against HepG-2 cells. Concerning cancerous cells, the order of selectivity of the synthesised analogues towards the tested carcinomas is MDA-MB-231 > MCF-7 > HepG-2. Meanwhile, the strongest cytotoxic and selective compound against the examined cancer cells is compound **5**, followed by **6** and **11**, with compound **4** coming last **(**Table 3**)**.

**Table 3.** Selectivity indices of the superior compounds.

Compound’s number	Cytotoxicity against WI-38 IC_50_^a^ (µM) ± SD	Selectivity index (SI)^b^		
	WI-38	MCF-7	MDA-MB-231	HepG-2
**4**	57.88 ± 3.2***	3.79	7.02	1.37
**5**	35.45 ± 2.2***	9.87	12.71	5.46
**6**	71.22 ± 3.7***	7.50	5.97	8.15
**11**	52.52 ± 2.9***	7.63	9.36	2.44
**Doxorubicin**	6.72 ± 0.5	1.61	2.11	1.49
**Roscovitine**	10.65 ± 0.8	1.47	1.39	1.16

The statistical significance was assessed by one-way ANOVA followed by Tukey’s post hoc test. GraphPad InStat (version 3.06) was used for analysis.

*** Significantly different from doxorubicin or roscovitine at *p* < 0.001.

^a^ IC_50_ is the average ± SD of three experiments.

^b^ SI: ≥5 (very selective), >2 (moderately selective), and <2 (not selective).

#### CDK-2 inhibition

CDK-2 overexpression correlates with the advancement of multiple types of malignancy, such as hepatic and breast tumours^56^. CDK is a prime option for pharmacological treatments in cancer therapy because of its broad function in proliferation^50^. The most effective and selective entities, **5**, **6**, and **11**, were selected for further testing to elucidate their molecular pathways based on the cytotoxicity assessment results. Consequently, the inhibition of CDK-2 was assessed for these compounds using roscovitine as a standard CDK-2 inhibitor. Compound **11**, containing dimethylbiphenylbis(tetrahydrobenzo[*b*]thiophene-3-carboxamide), exhibited the highest CDK-2 inhibition with an IC_50_ of 0.096 µM that exceeded roscovitine by nearly threefold. Furthermore, dimethoxybiphenyltetrahydrobenzo[*b*]thiophene-3-carboxamide **5** displayed promising CDK-2 inhibition with an IC_50_ of 0.245 µM, which is nearly identical to that of roscovitine (Table 4). Moreover, dimethylbiphenyltetrahydrobenzo[*b*]thiophene-3-carboxamide **6** showed a considerable CDK-2 inhibition with an IC_50_ of 0.852 µM.

**Table 4.** CDK-2 inhibition.

Compound number	IC_50_ (µM) ± SD^a^
**5**	0.245 ± 0.009
**6**	0.852 ± 0.031***
**11**	0.096 ± 0.003***
**Roscovitine**	0.288 ± 0.011

The statistical significance was assessed by one-way ANOVA followed by Tukey’s post hoc test. GraphPad InStat (version 3.06) was used for analysis.

*** Significantly different from roscovitine at *p* < 0.001.

^a^ The data were computed following three consecutive experiments.

From previous data, bis-carboxamide architecture in **11** exhibited the most potent CDK-2 inhibition, which could enable additional hydrogen-bonding interactions within the hinge region and enhanced occupancy of the ATP-binding cleft. However, compound **5** (with dimethoxy groups) displayed stronger CDK-2 inhibition than compound **6** (with dimethyl ones), as methoxy groups could provide electron density and potentially stabilise ligand–enzyme interactions.

#### Apoptosis induction

**Figure 5.**
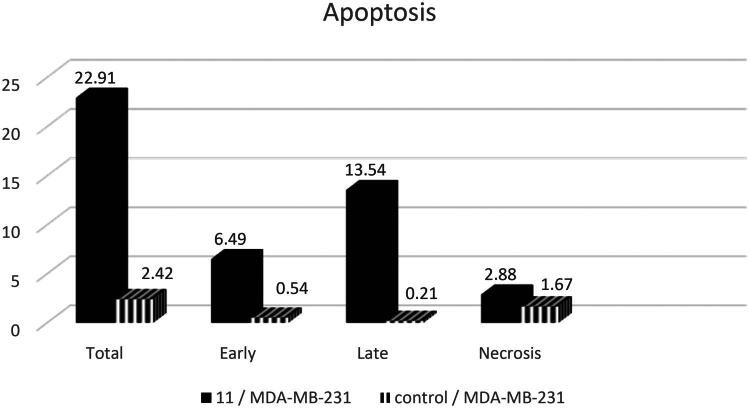
Apoptosis assay of **11**.

**Figure 6.**
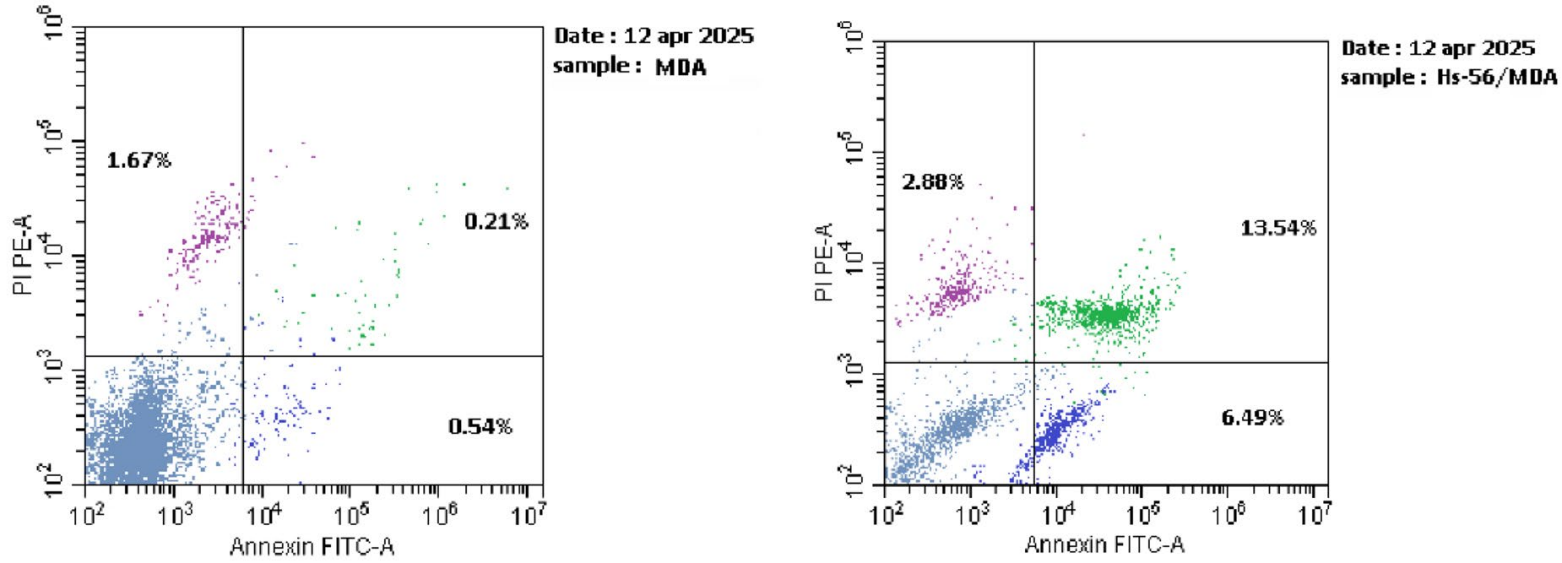
Apoptosis of MDA-MB-231 cells on treatment with control (at left) and **11** (at right). The lower left quadrants showed live cells (AV-negative/PI-negative), the lower right quadrants represented early apoptotic cells (AV-positive/PI-negative), the upper right quadrants showed late apoptotic cells or necrotic cells (AV-positive/PI-positive), and the upper left quadrants represented necrotic cells (AV-negative/PI-positive).

Apoptosis is a programmed form of cell death that occurs during normal cell proliferation and is induced by antitumor drugs. Many anticancer medications induce apoptosis as a crucial mechanism of action. In this study, compound **11**, the most potent CDK-2 inhibitor, was selected for further investigation to confirm its mechanism by assessing apoptosis in the most sensitive carcinoma, MDA-MB-231 cells, using the annexin V procedure^29^. The results demonstrated a distinct increase in both early and late apoptosis in MDA-MB-231 cells treated with compound **11**, with cellular percentages 12.02- and 64.48-fold higher than control, respectively (Figure 5 and Table S1). Also, the percentage of necrosis was elevated somewhat, exceeding the control by nearly 1.72-fold. Consequently, **11** was supposed to exert its activity through apoptosis induction (Figure 6).

#### Cell cycle analysis

**Figure 7.**
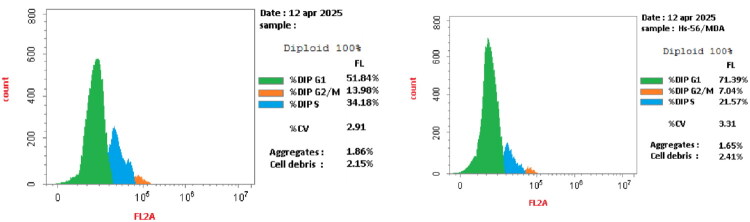
The effect of DMSO (left) and **11** (right) on MDA-MB-231 cell cycle.

CDK-2 regulates the G1/S and S/G2 phases, so its suppression is important for apoptosis induction and cell cycle arrest^57^. Herein, analogue **11** was selected to evaluate its impact on the MDA-MB-231 cell cycle to determine its mechanism. The outcomes showed that **11** increased cell accumulation in the G0/G1 phase to 71.39% compared with the control (51.84%). A concurrent increase was observed in this diminishment at the S and G2/M phases (Table 5). Hence, entity **11** induced apoptosis and arrested the cell cycle at the G0/G1 phase (Figure 7).

**Table 5.** Cell cycle analysis.

Code	G0/G1 (%)	S phase (%)	G2/M (%)
**11**	71.39 ± 4.23	21.57 ± 1.56	7.04 ± 0.61
Control	51.84 ± 2.75	34.18 ± 2.27	13.98 ± 1.14

### *In silico* studies

#### Molecular orbital computation

Geometry optimisations of compounds **2**–**11** were performed using the hybrid density functional theory (DFT) method at the B3LYP/6-31G(d,p) level as implemented in Gaussian 09W. The DFT calculations were carried out to obtain optimised ground-state geometries, frontier molecular orbital energies (HOMO and LUMO), dipole moments, and global reactivity descriptors. The parent compound **1** had previously been characterised by single-crystal X-ray diffraction and DFT analysis^25^.

**Figure 8.**
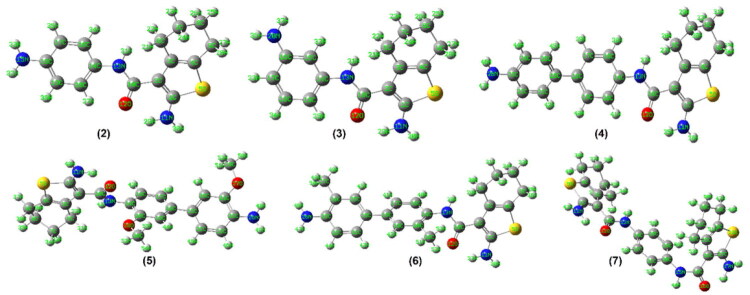
The energy optimised structures of **2**–**7**.

**Figure 9.**
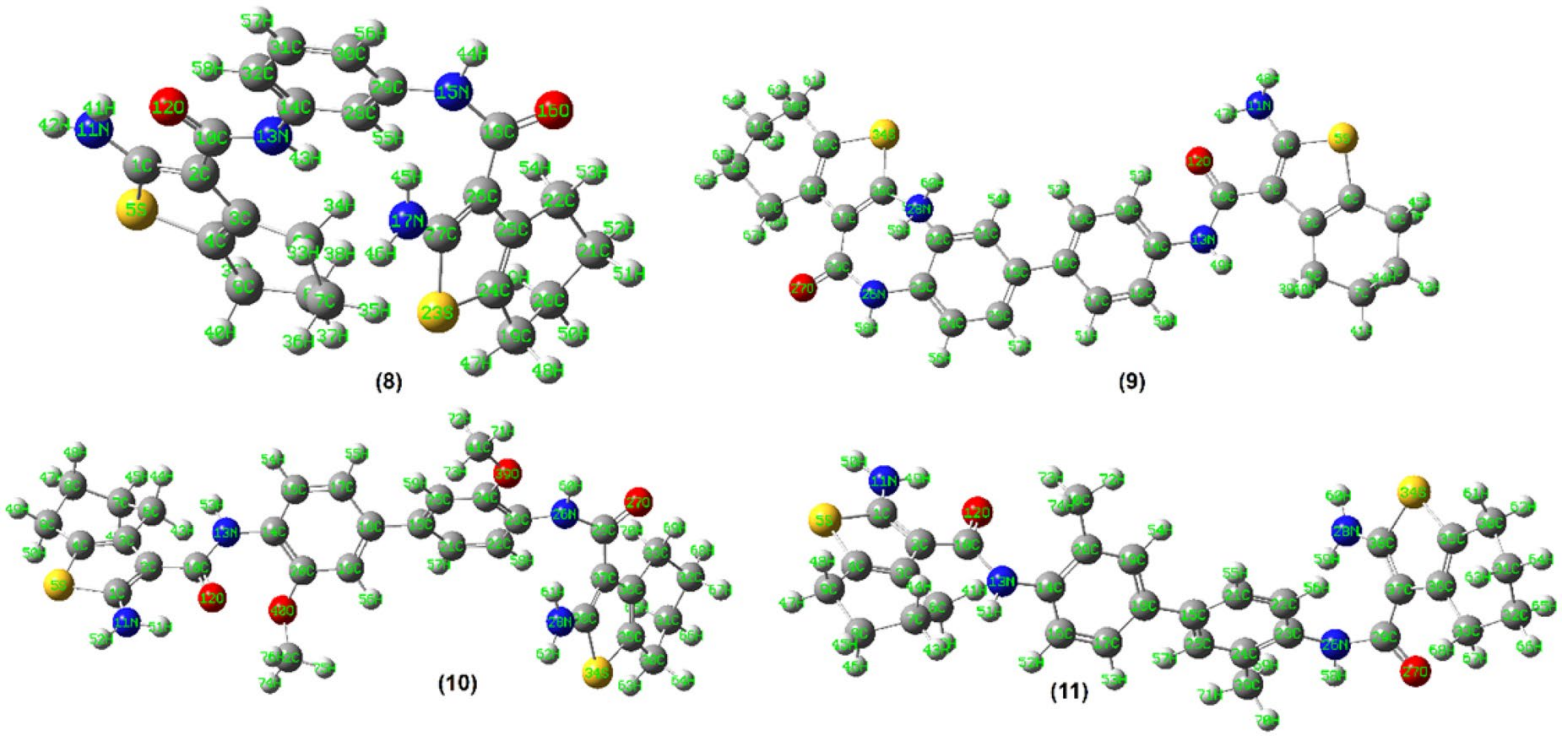
The energy optimised structures of **8**–**11**.

Before DFT optimisation, preliminary conformational searches and geometry refinements were performed using molecular mechanics (MMFF94x force field). The energy values reported in Figures 8 and 9 (kcal/mol) correspond to MM minimisation energies and represent relative conformational strain within the same force-field framework. These values do not represent DFT total electronic energies and should not be interpreted as absolute thermodynamic stability indicators or compared across molecules of different sizes.

The DFT total energies reported in Table 6 (eV) correspond to raw electronic energies obtained from Gaussian calculations. As these energies scale with molecular size and electron count, they are not directly comparable between different derivatives and are reported only for completeness.

**Table 6.** The global chemical reactivity descriptors for the prepared analogues.

Parameter	1^a^	2	3	4	5	6	7	8	9	10	11
Total energy (eV)	−28 066	−33 178	−33 178	−39 464	−45 694	−41 603	−57 026	−57 026	−63 300	−69 542	−65 451
*DM* (Debye)	0.71	2.74	3.97	2.58	2.39	2.82	4.51	3.09	8.56	5.09	4.04
*HOMO* (eV)	−5.16	−4.64	−4.95	−4.77	−4.73	−4.84	−5.00	−5.20	−4.63	−5.08	−5.13
*LUMO* (eV)	−0.53	−0.31	−0.46	−0.61	−0.39	−0.44	−0.85	−0.82	−2.45	−1.00	−0.98
Δ*E* (eV)	4.63	4.33	4.49	4.16	4.34	4.40	4.14	4.38	2.18	4.07	4.15
*Χ* (eV)	2.85	2.47	2.71	2.69	2.56	2.64	2.93	3.01	3.54	3.04	3.06
*V* (eV)	−2.85	−2.47	−2.71	−2.69	−2.56	−2.64	−2.93	−3.01	−3.54	−3.04	−3.06
*EA* (eV)	0.53	0.31	0.46	0.61	0.39	0.44	0.85	0.82	2.45	1.00	0.98
*IP* (eV)	5.16	4.64	4.95	4.77	4.73	4.84	5.00	5.20	4.63	5.08	5.13
*η* (eV)	2.31	2.17	2.24	2.08	2.17	2.20	2.07	2.19	1.09	2.04	2.08
*S* (eV)	1.16	1.08	1.12	1.04	1.09	1.10	1.04	1.09	0.55	1.02	1.04
*ω* (eV)	1.75	1.41	1.63	1.74	1.51	1.58	2.07	2.07	5.74	2.27	2.25

^a^ Ref^25^.

The optimised geometries reveal that compounds **2**–**7** adopt unsymmetrical conformations (*C1* point group). Intramolecular N–H···O hydrogen bonds were observed in several derivatives, with N–H···O distances ranging from 1.62 to 1.92 Å, supporting conformational stabilisation. In compounds **5** and **6**, the substituted biphenyl fragments adopt nearly perpendicular orientations relative to the parent core (dihedral angles of 88.1° and 76.3°, respectively), reflecting steric effects imposed by methoxy and methyl substituents. Compound **7** exhibits partial bending of one parent moiety towards the central phenyl ring (dihedral angle ≈35°), suggesting possible intramolecular π–π interactions.

#### Global reactivity descriptors

Global reactivity descriptors were calculated from the DFT-derived *HOMO* and *LUMO* energies, including the energy gap (Δ*E*), electronegativity (*χ*), chemical potential (*V*), ionisation potential (*IP*), electron affinity (*EA*), global hardness (*η*), softness (*S*), and electrophilicity index (*ω*). The *HOMO–LUMO* energy gap (Δ*E*) reflects electronic excitation energy and charge-transfer tendency rather than biological potency.

**Figure 10.**
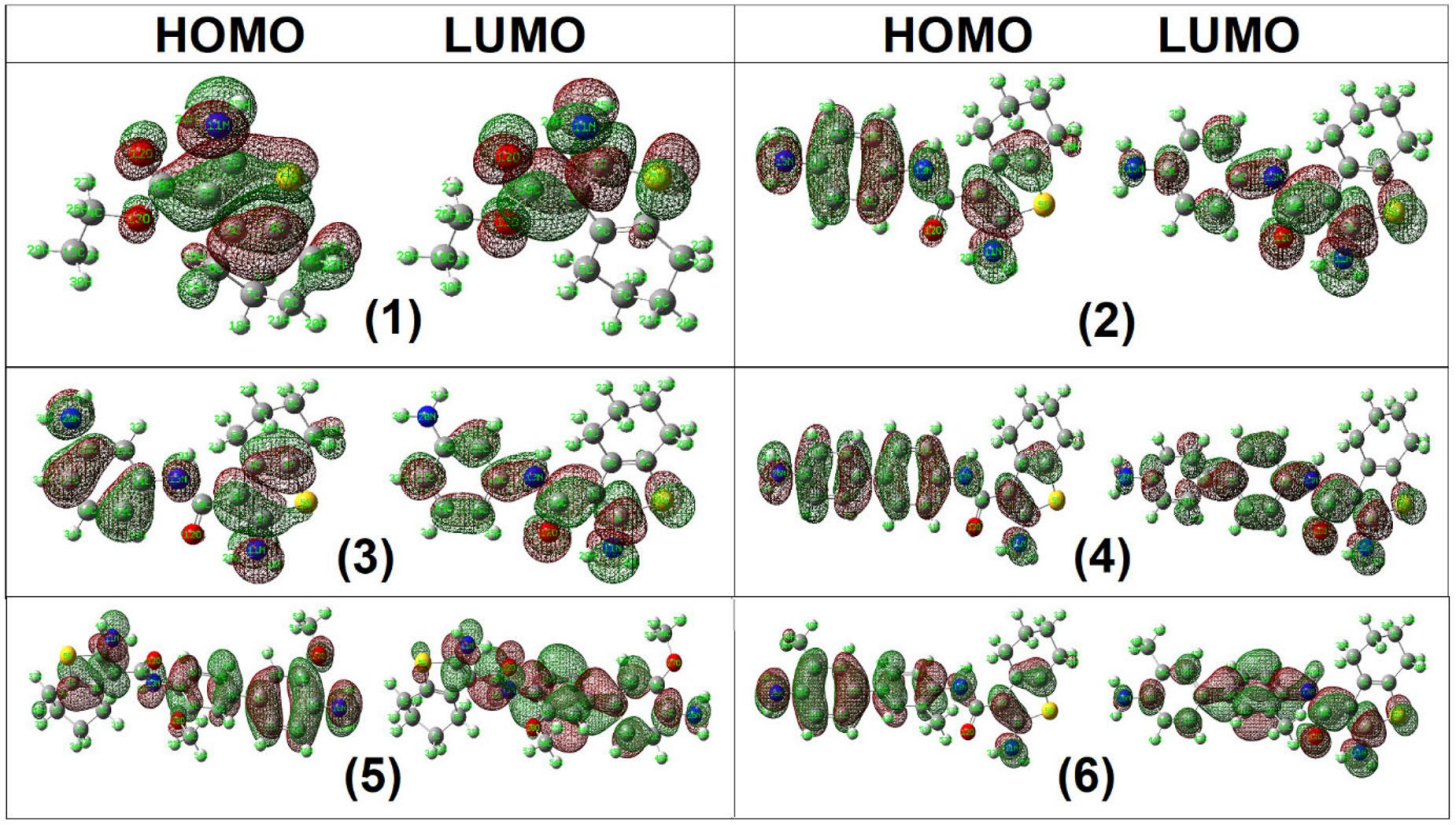
The frontier *HOMO* and *LUMO* orbitals of analogues **1**–**6**.

**Figure 11.**
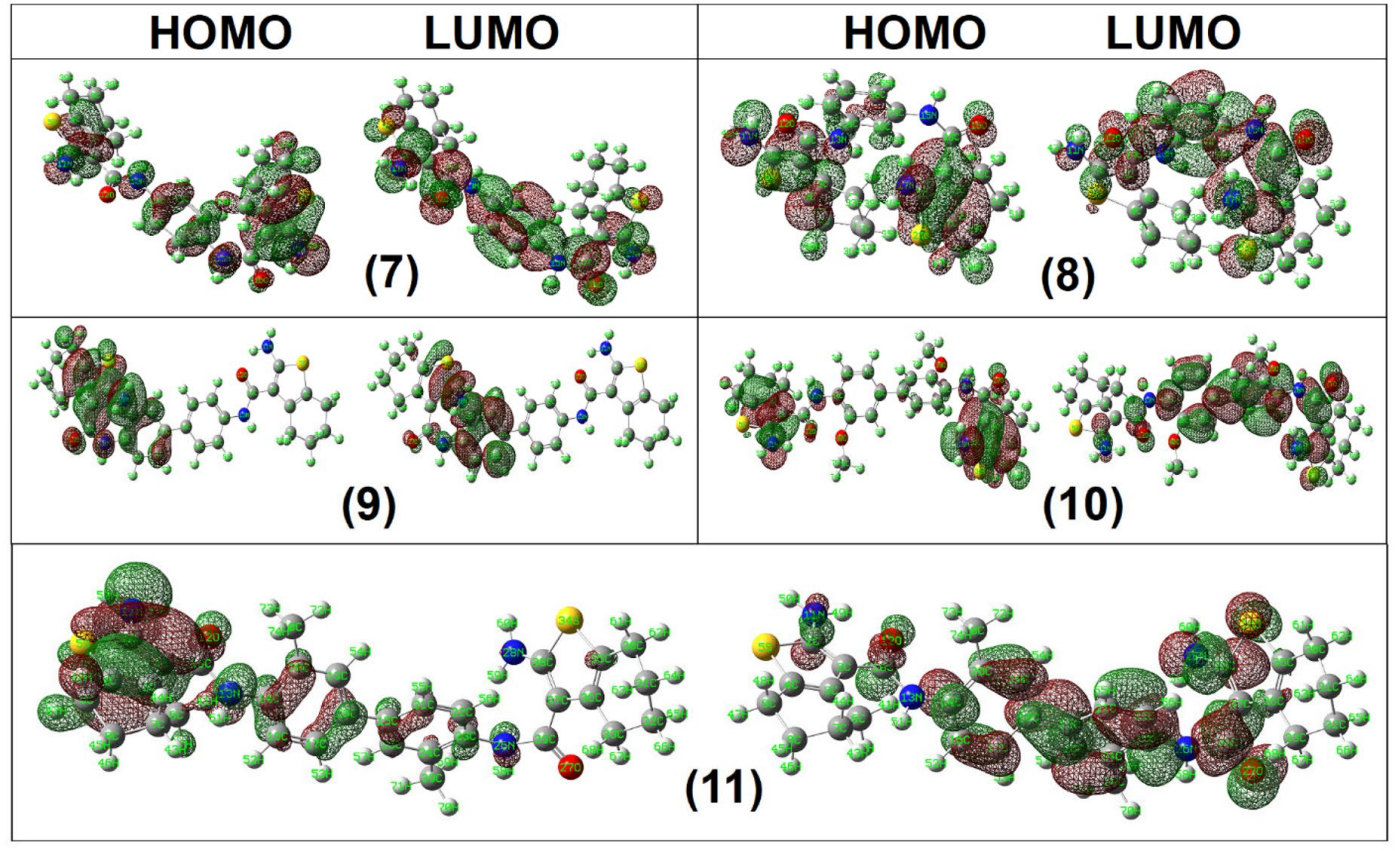
The frontier *HOMO* and *LUMO* orbitals of analogues **7**–**11**.

Among the studied compounds, derivative **9** exhibited the smallest Δ*E* value (2.18 eV), whereas the remaining derivatives showed Δ*E* values in the range of 4.07–4.49 eV. Compound **9** also displayed the highest *EA* (2.45 eV), lowest hardness (*η* = 1.09 eV), and highest electrophilicity index (*ω* = 5.74 eV), indicating increased electronic softness and charge-accepting capability (Table 6, Figures 10 and 11).

However, no direct quantitative correlation was observed between Δ*E*, electrophilicity index, or other global descriptors and the experimentally measured biological activity. Notably, although compound **9** reveals the highest electrophilicity and smallest energy gap, it is not among the most biologically active derivatives. This indicates that cytotoxic potency is governed by multiple factors, including target binding interactions, steric complementarity, and pharmacokinetic properties, rather than frontier orbital parameters alone. Therefore, the DFT descriptors are discussed here as indicators of intrinsic electronic properties and molecular reactivity trends, without implying predictive SARs.

#### Molecular docking analysis

Molecular docking was applied to the most potent cytotoxic analogues, tetrahydrobenzo[*b*]thiophene carboxamides **5**, **6**, and **11**, to explain differences in their enzyme inhibitory activity and to justify their *in vitro* cytotoxicity. Docking simulations were carried out using the Molecular Operating Environment (MOE-Dock) software, version 2024.0601^58^^,^^59^. The co-crystallised structure of CDK-2/cyclin A2 with its natural ligand, roscovitine (PDB code: 3DDQ), has been deposited in the Protein Data Bank^60^^,^^61^. When the docking procedure was verified using the original ligand, energy scores of −11.26 kcal/mol and a low RMSD of 0.79 Å between the docked pose and the co-crystallised structure were obtained. Purine’s H-bond interactions with **Glu81** and **Leu83** in the hinge region enabled roscovitine to fit into the CDK-2/cyclin A2 binding pocket, as reported^62^. Furthermore, the benzyl moiety established an arene-H contact with **Ile10**, whereas benzylamino showed further H-bonding with the **Leu83** backbone.

**Figure 12.**
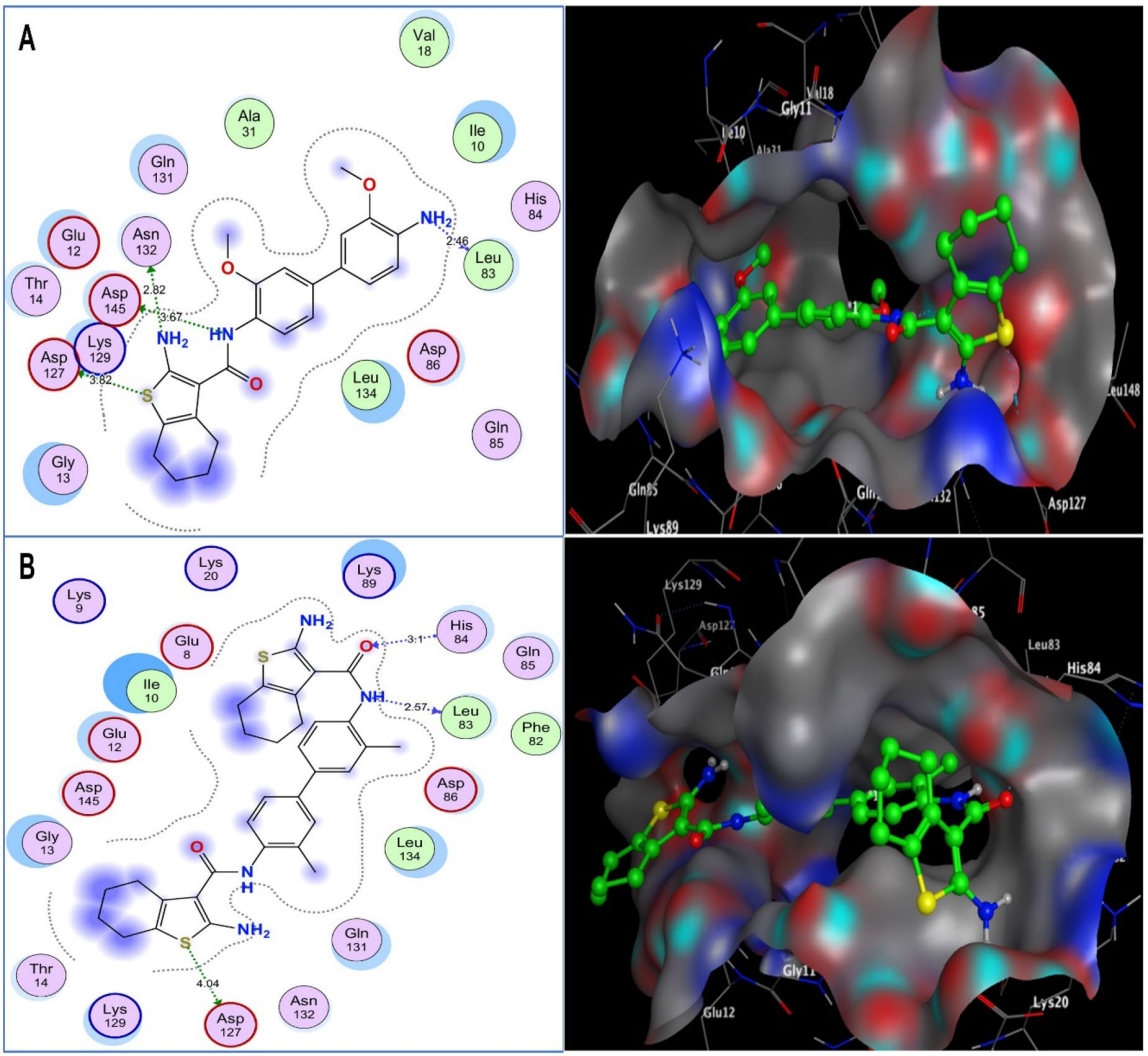
(A, B) Diagrams demonstrate two and three-dimensional views of the excellent tetrahydrobenzo[*b*]thiophene carboxamides **5** and **11** within CDK-2/cyclin A2 active site (PDB code: 3DDQ), respectively.

**Figure 13.**
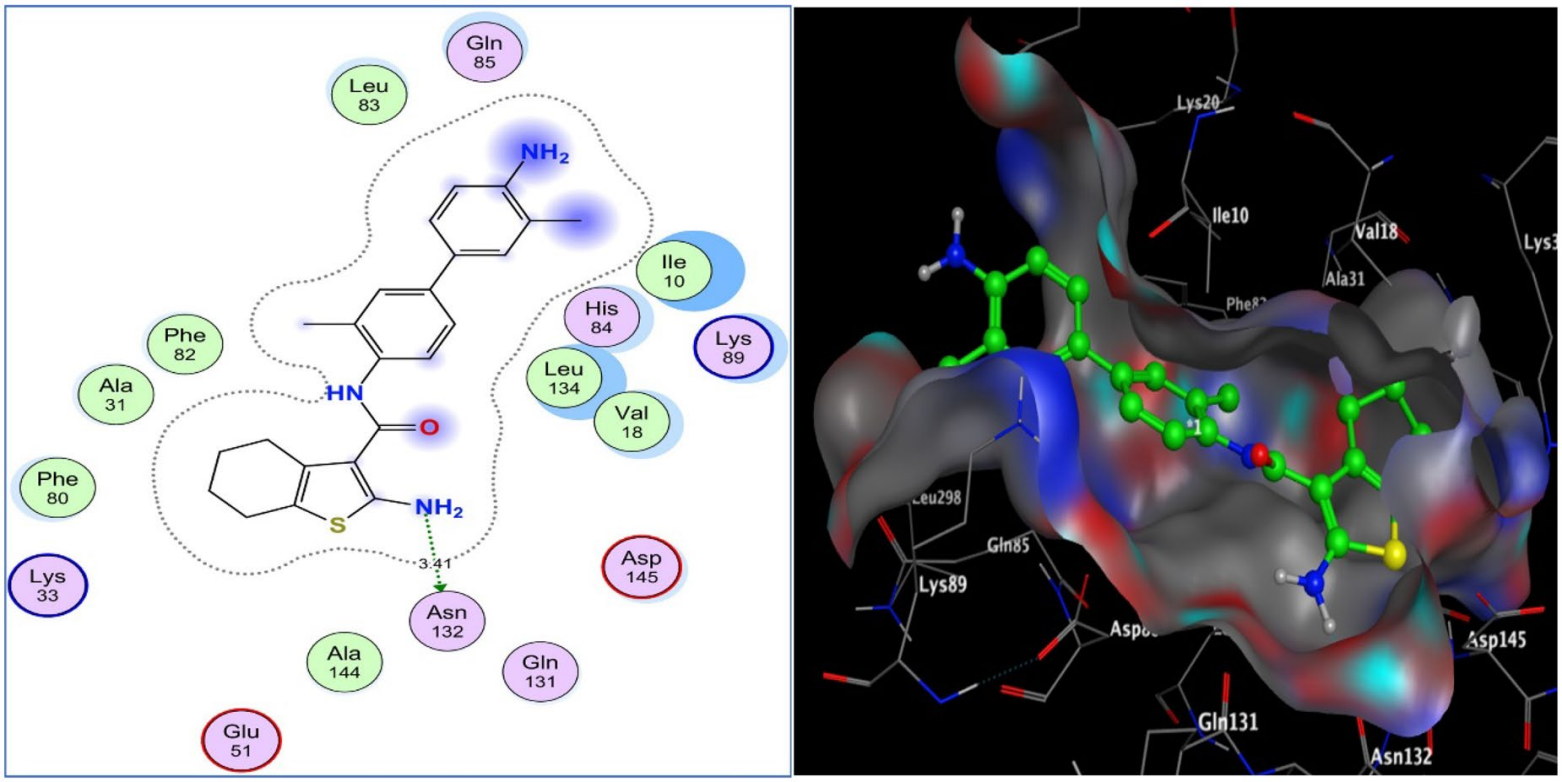
Two- and three-dimensional views of the weak tetrahydrobenzo[*b*]thiophene carboxamide **6** within CDK-2/cyclin A2 active site (PDB code: 3DDQ).

As depicted in Figures 12 and 13, with energy scores of −10.62, −8.19, and −11.18 kcal/mol, respectively, the screened tetrahydrobenzo[*b*]thiophene carboxamides **5**, **6**, and **11** were appropriately implemented in CDK-2/cyclin A2. Sulphur of thiophene in both **5** and **11** shared weak hydrogen bonds with the **Asp127** sidechain (distances: 3.82 and 4.04 Å, respectively). Nitrogens of the amino group at C-2 of tetrahydrobenzo[*b*]thiophene and the carboxamide in **5** donated two H-bonds with the sidechains of **Asn132** and **Asp145** (distances: 3.67 and 2.82 Å, respectively). Additionally, the **Leu83** backbone in the hinge region of CDK-2 displayed hydrogen bondin inhibitors in cancer therapy: an g with the nitrogens of the amino group at p-4 of the biphenyl moiety in **5** and the carboxamide fragment in the remaining part of the **11** molecule (distances: 2.46 and 2.57 Å, respectively), resembling roscovitine. Analogue **11** showed a further H-bond acceptor between the His84 backbone and the carboxamide oxygen (distance: 3.1 Å). On the other hand, the weak inhibitory analogue **6** revealed only one H-bond between the amino nitrogen at C-2 of tetrahydrobenzo[*b*]thiophene and the sidechain of **Asn132** (distance: 3.41 Å).

Among the investigated tetrahydrobenzo[*b*]thiophene-3-carboxamide analogues, **5** and **11** demonstrated superior inhibitory potency, attributed to the ability to form multiple stabilising hydrogen bonds with key CDK-2 residues, including **Asp127**, **Leu83**, and **His84** in **11** and additional hydrogen bonds with **Asn132** and **Asp145** in **5**. These interactions collectively strengthened their accommodation within the ATP-binding pocket, exceeding the binding efficiency of the reference drug roscovitine.

## Conclusions

Regarding the advancement of potential CDK-2 inhibitors, two sets of 4,5,6,7-tetrahydrobenzo[*b*]thiophene carboxamides were synthesised using conventional and eco-friendly microwave-aided procedures. These analogues were assessed for their antitumor effects against hepatic HepG-2 and breast MCF-7 and MDA-MB-231 carcinomas, in which dimethoxy **5** and dimethyl-bearing analogues **6** and **11** demonstrated significant cytotoxicity and selectivity against the examined cancer cells. Consequently, they were chosen for further assays to determine their mechanism. The findings suggest that these compounds may exert cytotoxicity by inhibiting CDK-2. Compound **11**, containing dimethylbiphenylbis(tetrahydrobenzo[*b*]thiophene-3-carboxamide), displayed the highest CDK-2 inhibition with an IC_50_ of 0.096 µM that exceeded roscovitine by nearly threefold. Besides, it arrested the MDA-MB-231 cell cycle at the G0/G1 phase by apoptotic stimulation. Molecular modelling showed strong binding of the bioactive analogues to the active pocket of CDK-2 receptor, suggesting their potential as lead inhibitors.

## Supplementary Material

Supplementary_materials_Clean.docx

## Data Availability

The data that confirm the findings of this article are available in the supplementary material.
