# Supplementary material for: Design, synthesis, anticancer activity, and mechanistic investigation of 4,5,6,7-tetrahydrobenzo[b]thiophene carboxamides as CDK-2 inhibitors: in vitro and in silico DFT and molecular docking study
Source: J Enzyme Inhib Med Chem. 2026 May 29;41(1):2656826. doi: 10.1080/14756366.2026.2656826 (PMC13224697; doi:10.1080/14756366.2026.2656826)
Supplement: Supplementary_materials_Clean.docx [file IENZ_A_2656826_SM0892.docx]

**Design, synthesis, anticancer activity, and mechanistic investigation of benzo[*b*]thiophene carboxamides as CDK-2 inhibitors: *In vitro* and *in silico* DFT and molecular docking study**

**Supporting information**

List of contents

[1 Spectral Data of New Compounds 4](#_Toc167921757)

[1.1 Spectral data of compound 2 4](#_Toc167921758)

[1.1.1 IR Spectrum of 2 4](#_Toc167921759)

[1.1.2 ^1^H NMR Spectrum of 2 5](#_Toc167921760)

[1.1.3 ^13^C NMR Spectrum of 2 7](#_Toc167921761)

[1.1.4 Mass Spectrum of 2 8](#_Toc167921762)

[1.2. Spectral data of compound 3 9](#_Toc167921763)

[1.2.1. IR Spectrum of 3 9](#_Toc167921764)

[1.2.2. ^1^H NMR spectrum of 3 10](#_Toc167921765)

[1.2.3. ^13^C NMR spectrum of 3 12](#_Toc167921766)

[1.2.4. Mass spectrum of 3 13](#_Toc167921767)

[1.3. Spectral data of compound 4 14](#_Toc167921768)

[1.3.1. IR spectrum of 4 14](#_Toc167921769)

[1.3.2. ^1^H NMR spectrum of 4 15](#_Toc167921770)

[1.3.3. ^13^C NMR spectrum of 4 17](#_Toc167921772)

[1.3.4. Mass Spectrum of 4 18](#_Toc167921773)

[1.4. Spectral data of compound 5 19](#_Toc167921774)

[1.4.1. IR spectrum of 5 19](#_Toc167921775)

[1.4.2. ^1^H NMR spectrum of 5 20](#_Toc167921776)

[1.4.3. ^13^C NMR spectrum of 5 22](#_Toc167921778)

[1.4.4. Mass Spectrum of 5 23](#_Toc167921779)

[1.5. Spectral data of compound 6 24](#_Toc167921780)

[1.5.1. IR spectrum of 6 24](#_Toc167921781)

1.5.2. ^1^H NMR spectrum of 6 ………………………………………………………………………………….25

[1.5.3. ^13^C NMR spectrum of 6 27](#_Toc167921784)

[1.5.4. Mass Spectrum of 6 28](#_Toc167921785)

[1.6. Spectral data of compound 7 29](#_Toc167921786)

[1.6.1. IR spectrum of 7 29](#_Toc167921787)

[1.6.2. ^1^H NMR spectrum of 7 30](#_Toc167921788)

[1.6.3. ^13^C NMR spectrum of 7 32](#_Toc167921790)

[1.6.4. Mass Spectrum of 7 33](#_Toc167921791)

[1.7. Spectral data of compound 8 34](#_Toc167921792)

[1.7.1. ^1^H NMR spectrum of 8 34](#_Toc167921794)

[1.7.2. ^13^C NMR spectrum of 8 36](#_Toc167921796)

[1.7.3. Mass Spectrum of 8 37](#_Toc167921797)

[1.8. Spectral data of compound 9 38](#_Toc167921798)

[1.8.1. IR spectrum of 9 38](#_Toc167921799)

[1.8.2. ^1^H NMR spectrum of 9 39](#_Toc167921800)

[1.8.3. ^13^C NMR spectrum of 9 41](#_Toc167921802)

[1.8.4. Mass Spectrum of 9 42](#_Toc167921803)

[1.9. Spectral data of compound 10 43](#_Toc167921804)

[1.9.1. IR spectrum of 10 43](#_Toc167921805)

[1.9.2. ^1^H NMR spectrum of 10 44](#_Toc167921806)

[1.9.3. ^13^C NMR spectrum of 10 45](#_Toc167921808)

[1.9.4. Mass Spectrum of 10 46](#_Toc167921809)

[1.10. Spectral data of compound 11 47](#_Toc167921810)

[1.10.1. IR spectrum of 11 47](#_Toc167921811)

[1.10.2. ^1^H NMR spectrum of 11 48](#_Toc167921812)

[1.10.3. ^13^C NMR spectrum of 11 50](#_Toc167921814)

[1.10.4. Mass Spectrum of 11 51](#_Toc167921815)

[2 Specifications of instruments used for characterization of new compounds 52](#_Toc167921827)

[3 Curves of the new compounds against cancer cell lines in terms of cell viability 53](#_Toc167921828)

4. [Curves of active compounds against normal cell line in terms of cells viability 55](#_Toc167921829)

[5. CDK-2 inhibition raw data: 56](#_Toc167921830)

6. Molecular docking study…………………………………………………………………………………………..58

7. HPLC analysis for compounds 5, 6, and 11………………………………………………………………..59

8. CDK-2 inhibition assay…………………….……………………………………………………………………….70

9. Annexin-V-FITC apoptosis assay ……………………………………………………………………………….70

10. Cell cycle analysis……………………………………………………………………………………………………71

**Table S1**. Statistical fitting metrics and precision analysis for the cytotoxic activity of the synthesized compounds…………………………………………………………………………………………..….72

# Spectral Data of New Compounds

## Spectral data of compound 2

### IR Spectrum of 2


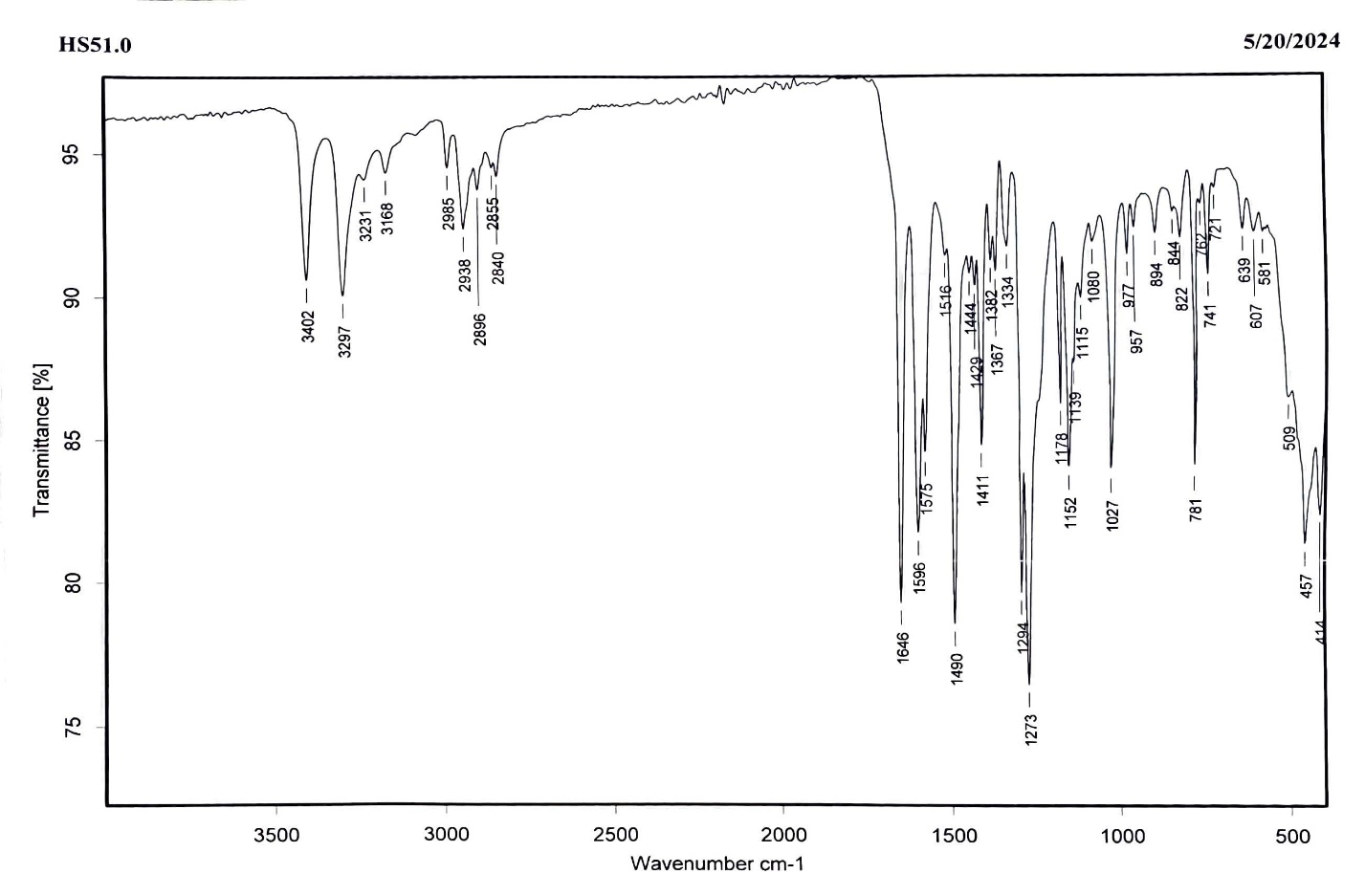

### ^1^H NMR Spectrum of 2

###
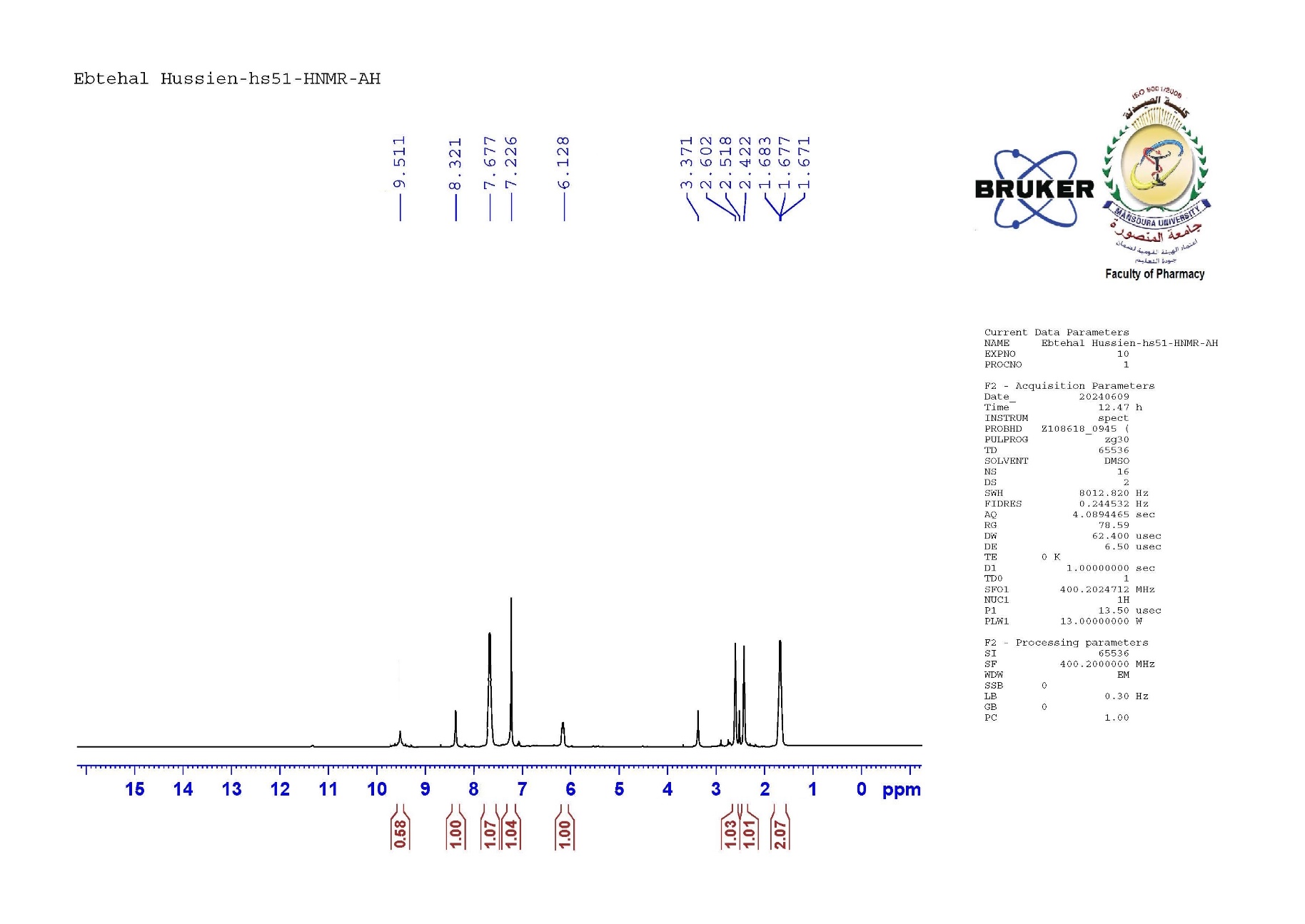

**^1^H NMR Spectrum of 2 (D_2_O)**


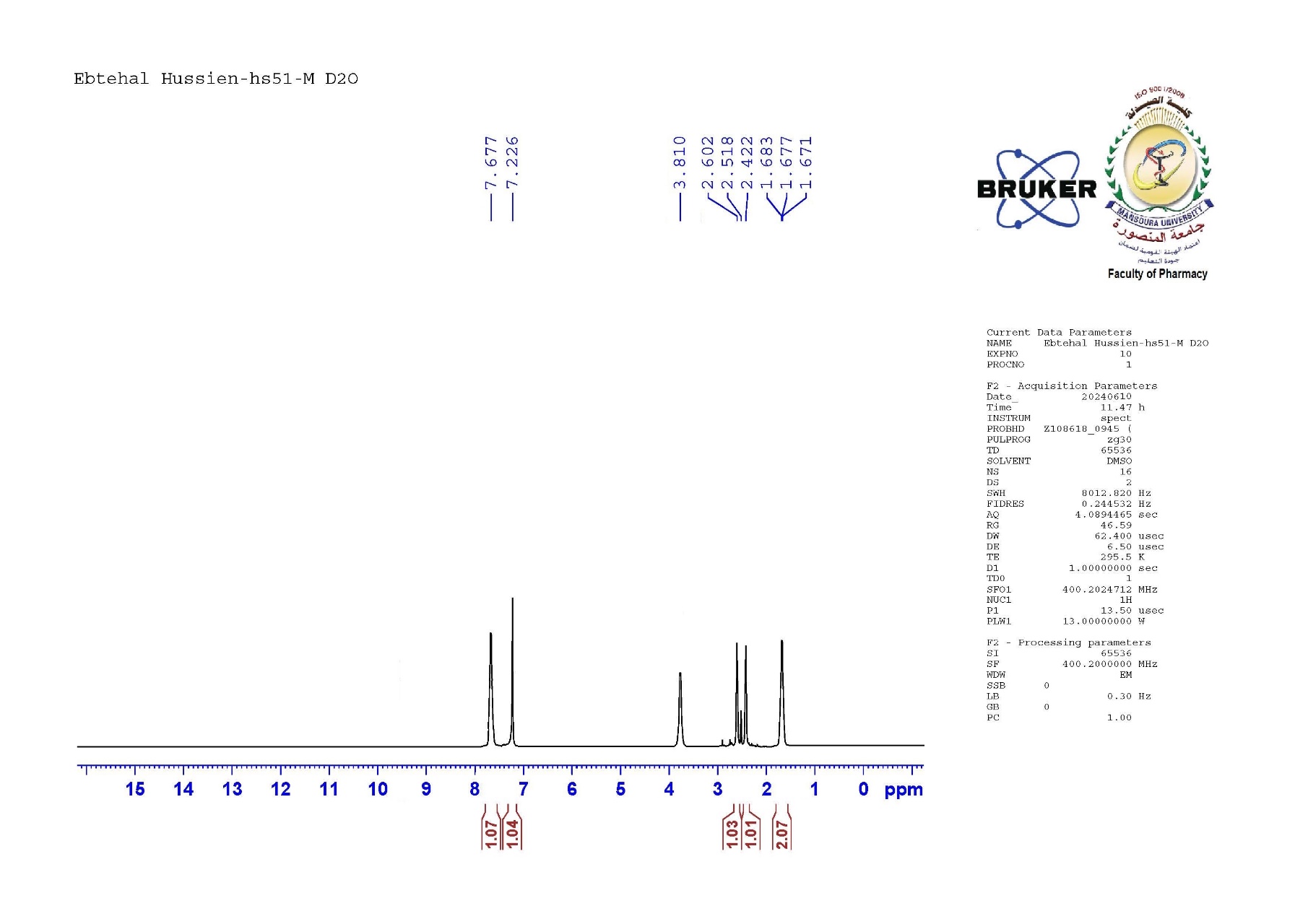

### ^13^C NMR Spectrum of 2


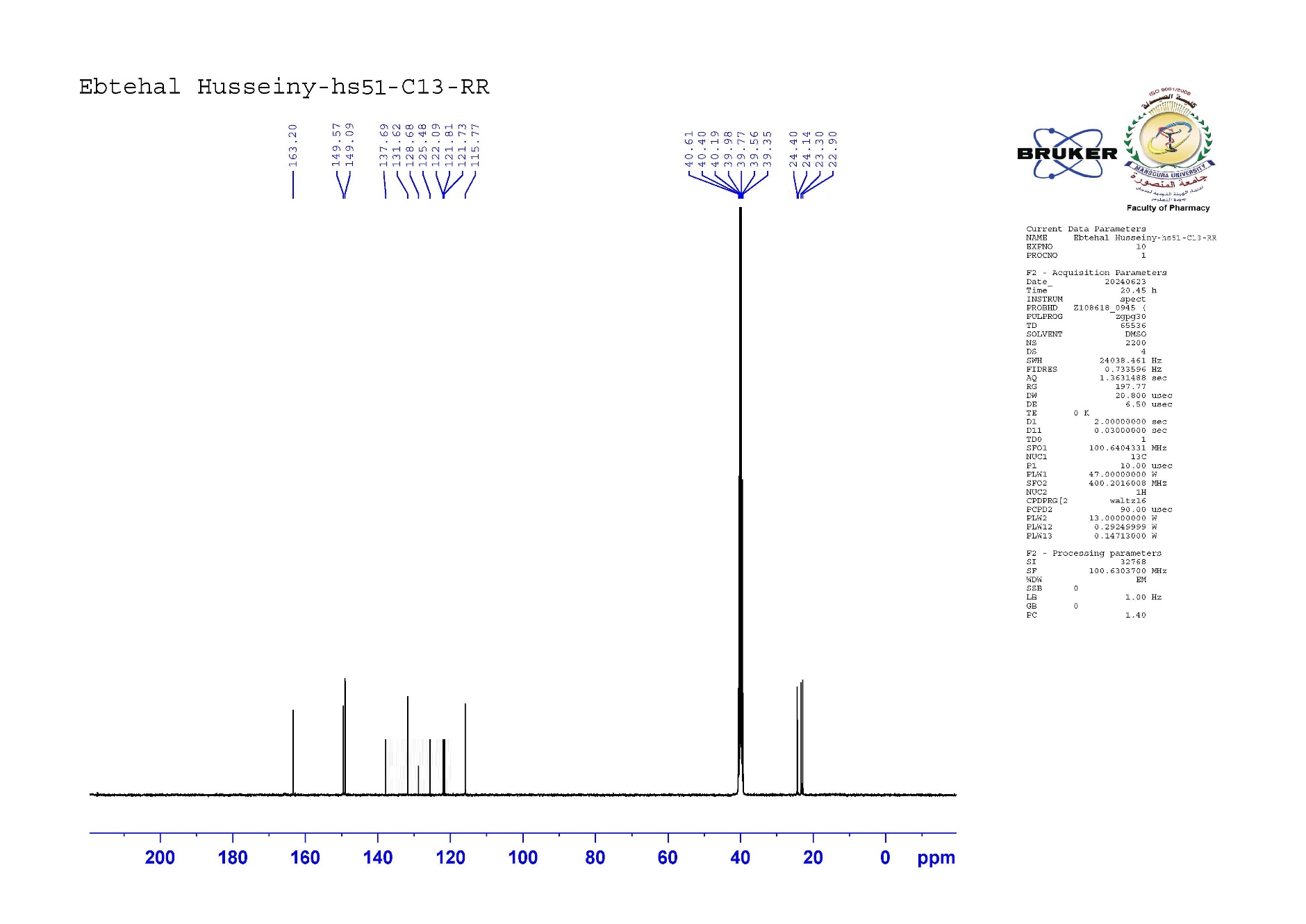

### Mass Spectrum of 2


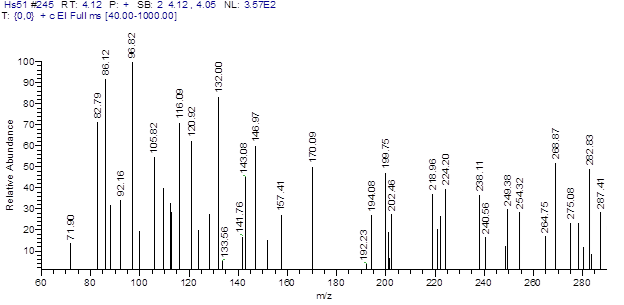

## Spectral data of compound 3

## IR Spectrum of 3


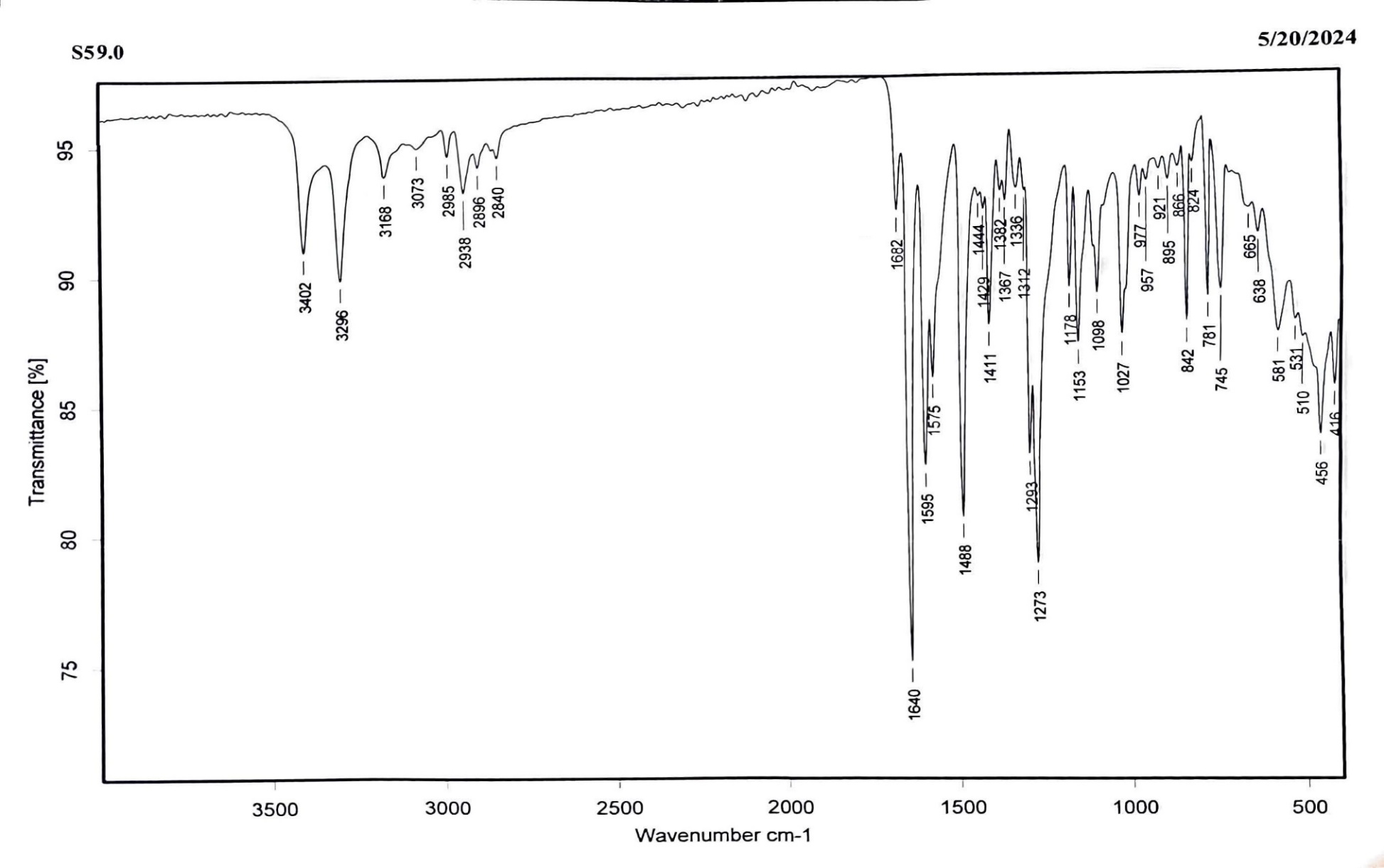

# ^1^H NMR spectrum of 3
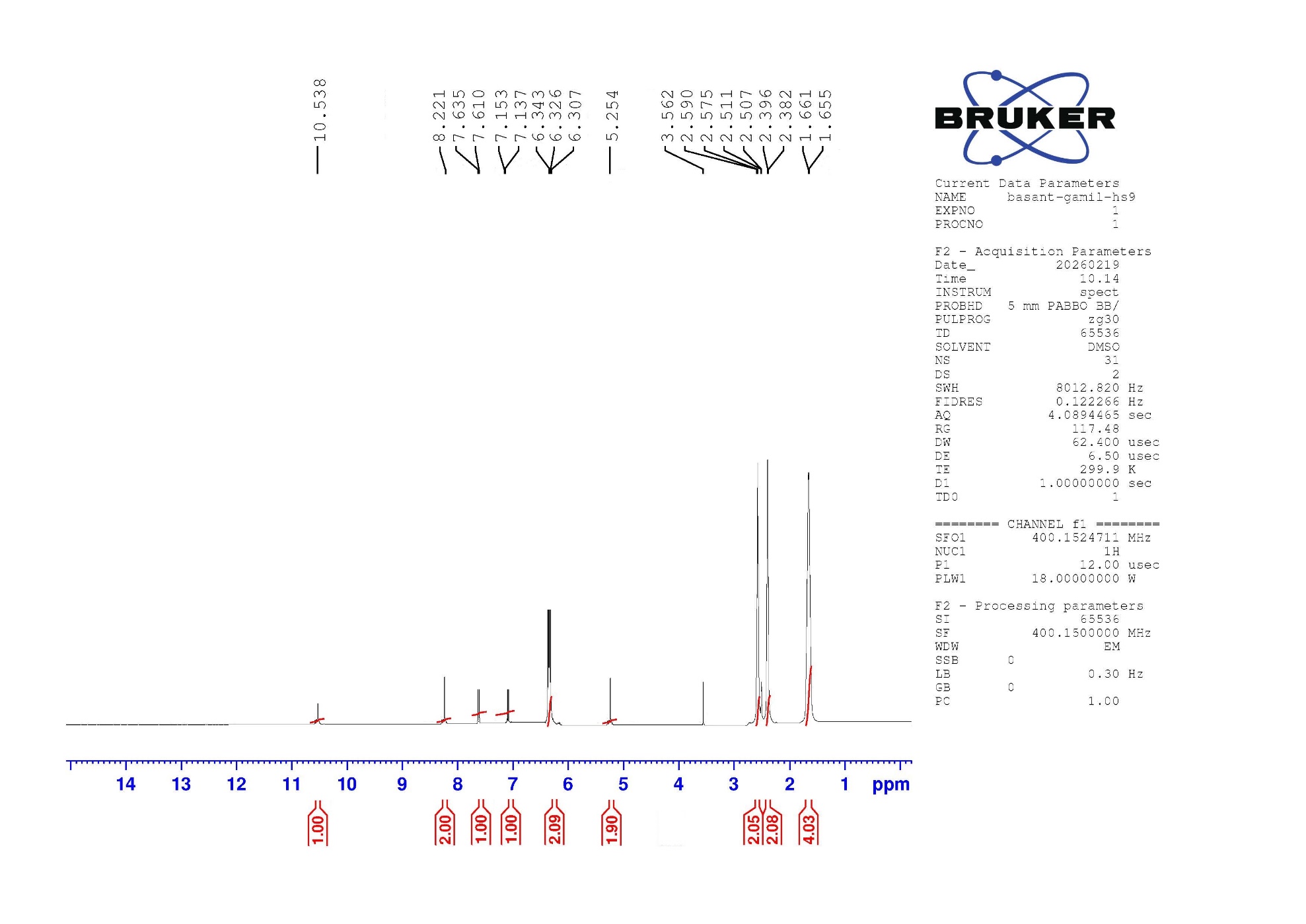

# ^13^C NMR spectrum of 3


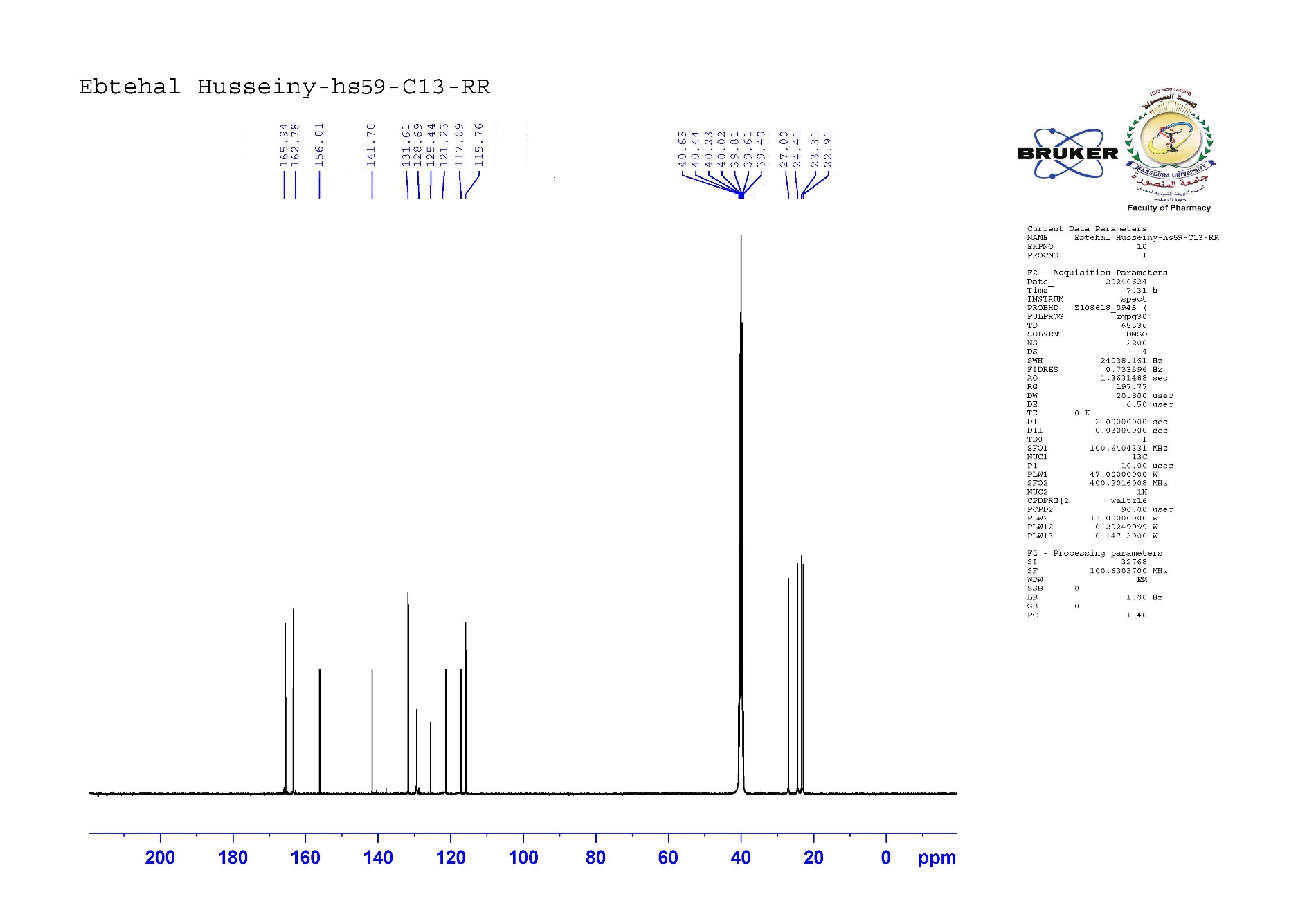

# Mass spectrum of 3


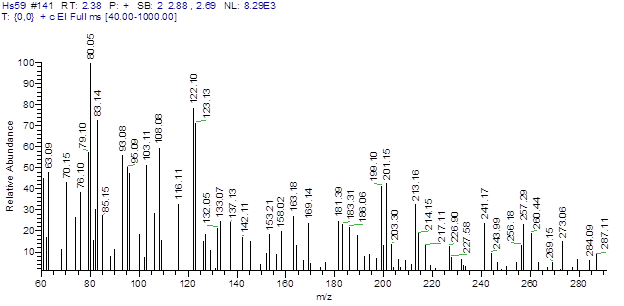


# Spectral data of compound 4

# IR spectrum of 4


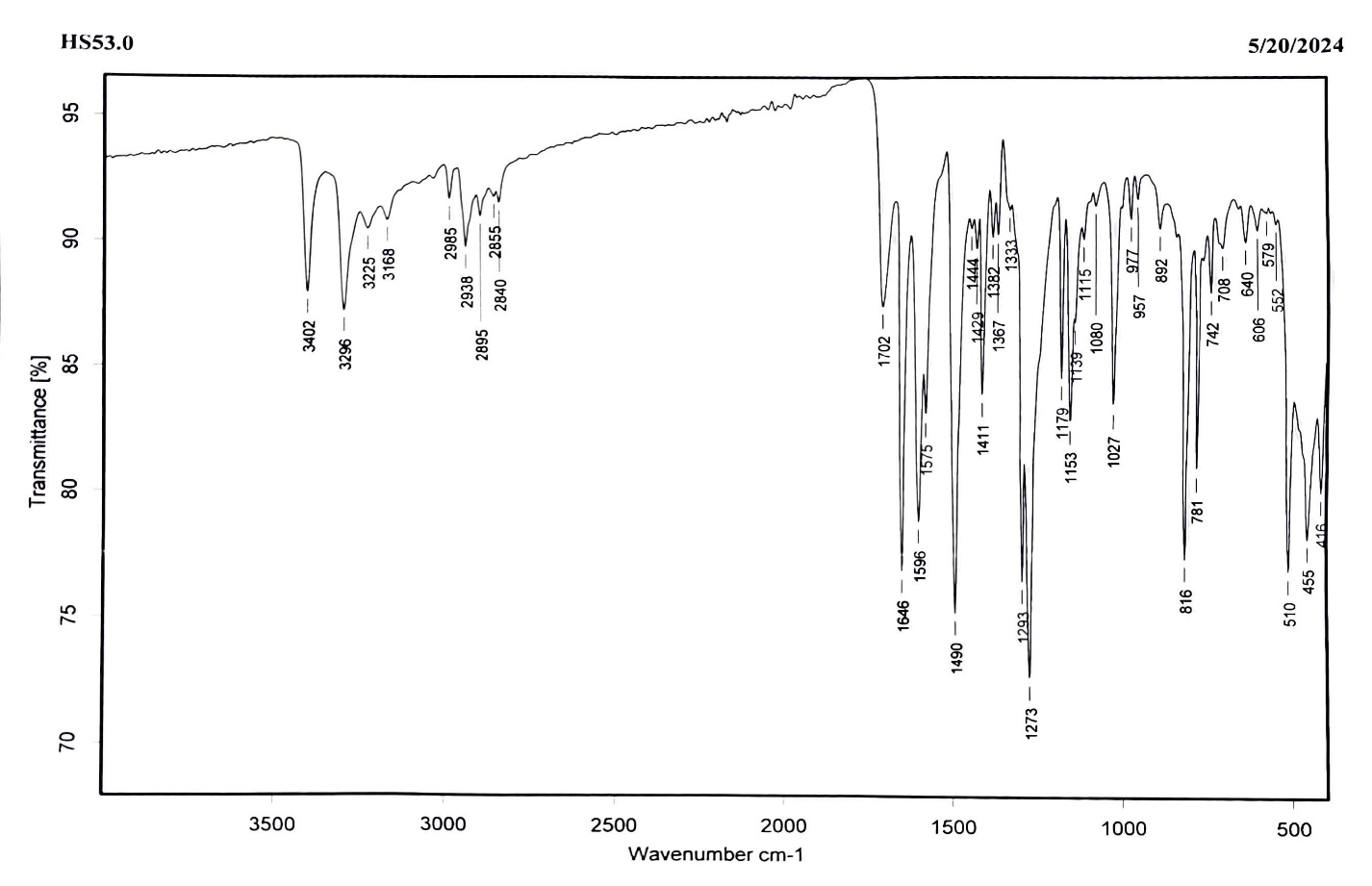

# ^1^H NMR spectrum of 4


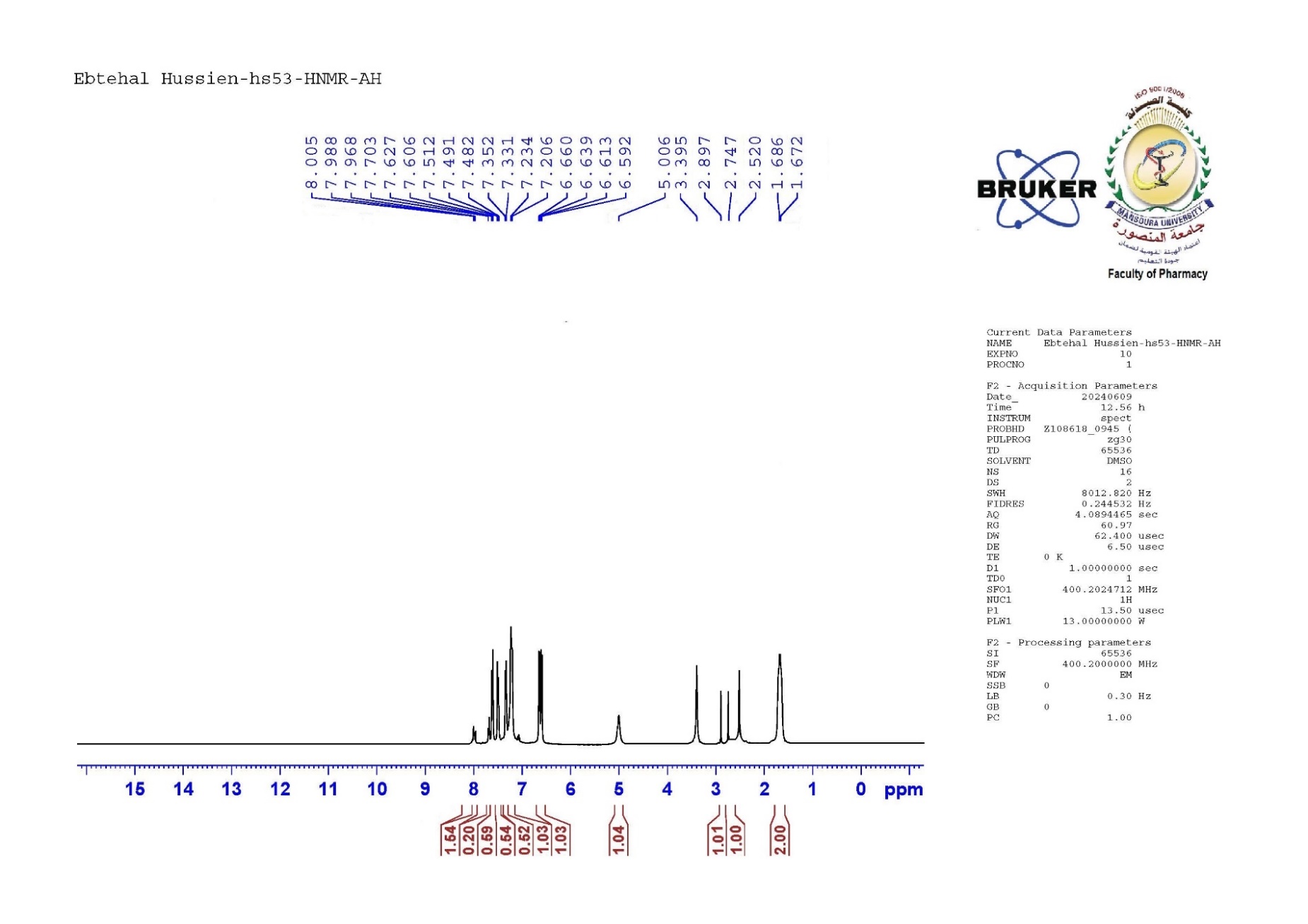

# ^1^H NMR spectrum of 4 (D_2_O)


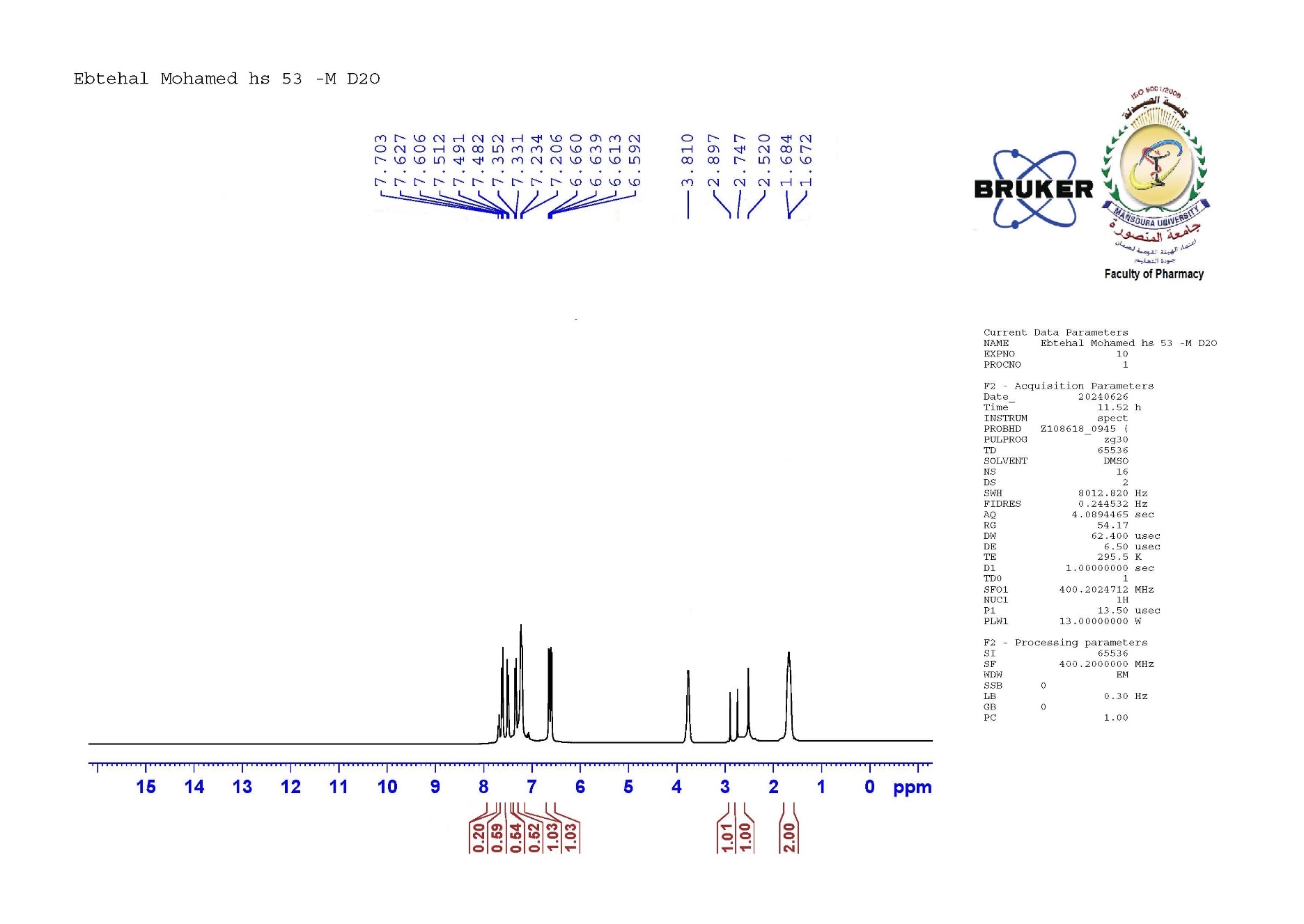

# ^13^C NMR spectrum of 4


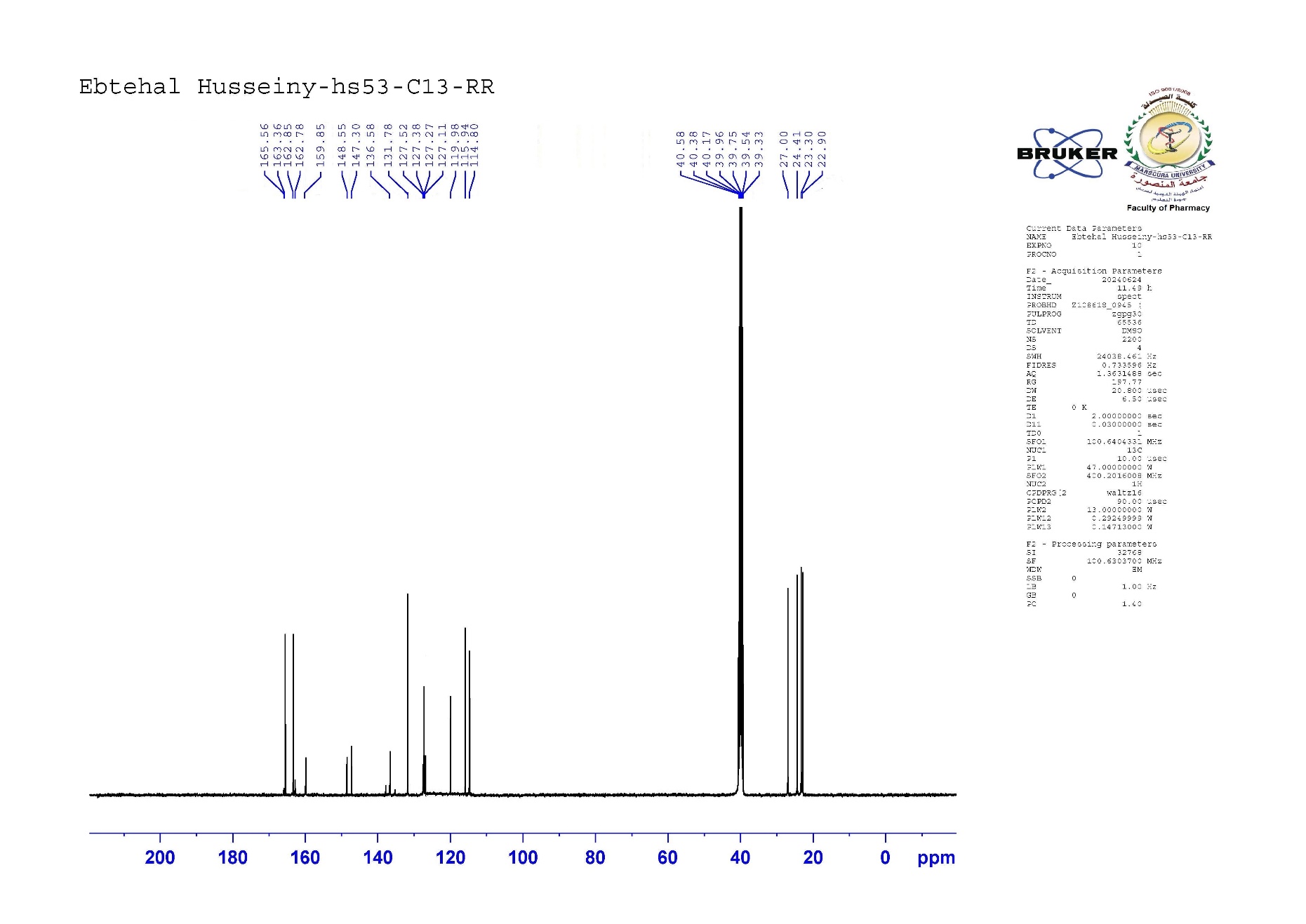

# Mass Spectrum of 4

#
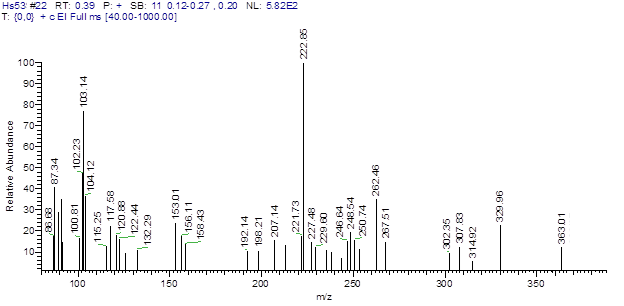

# Spectral data of compound 5

# IR spectrum of 5


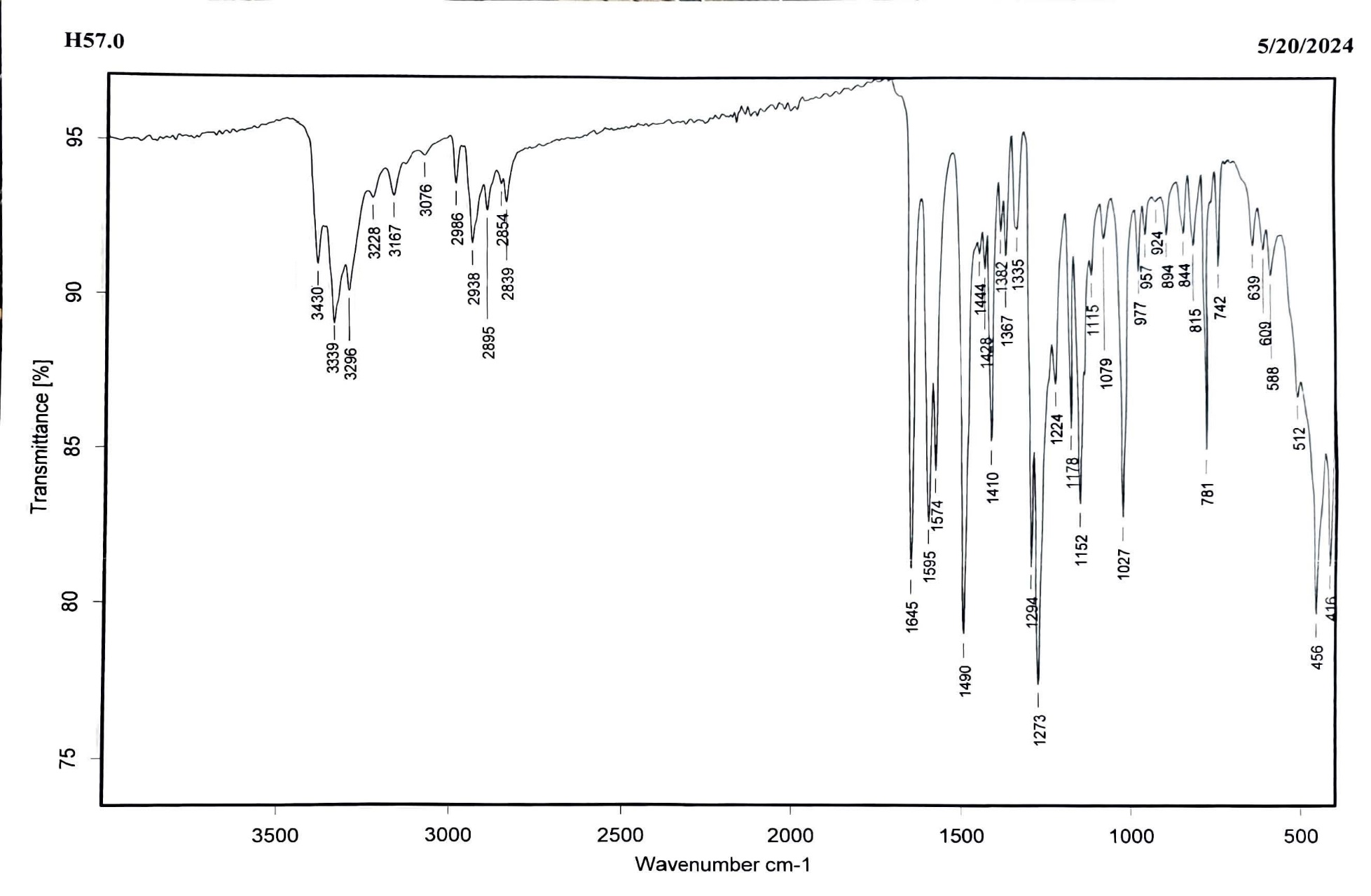

# ^1^H NMR spectrum of compound 5

#
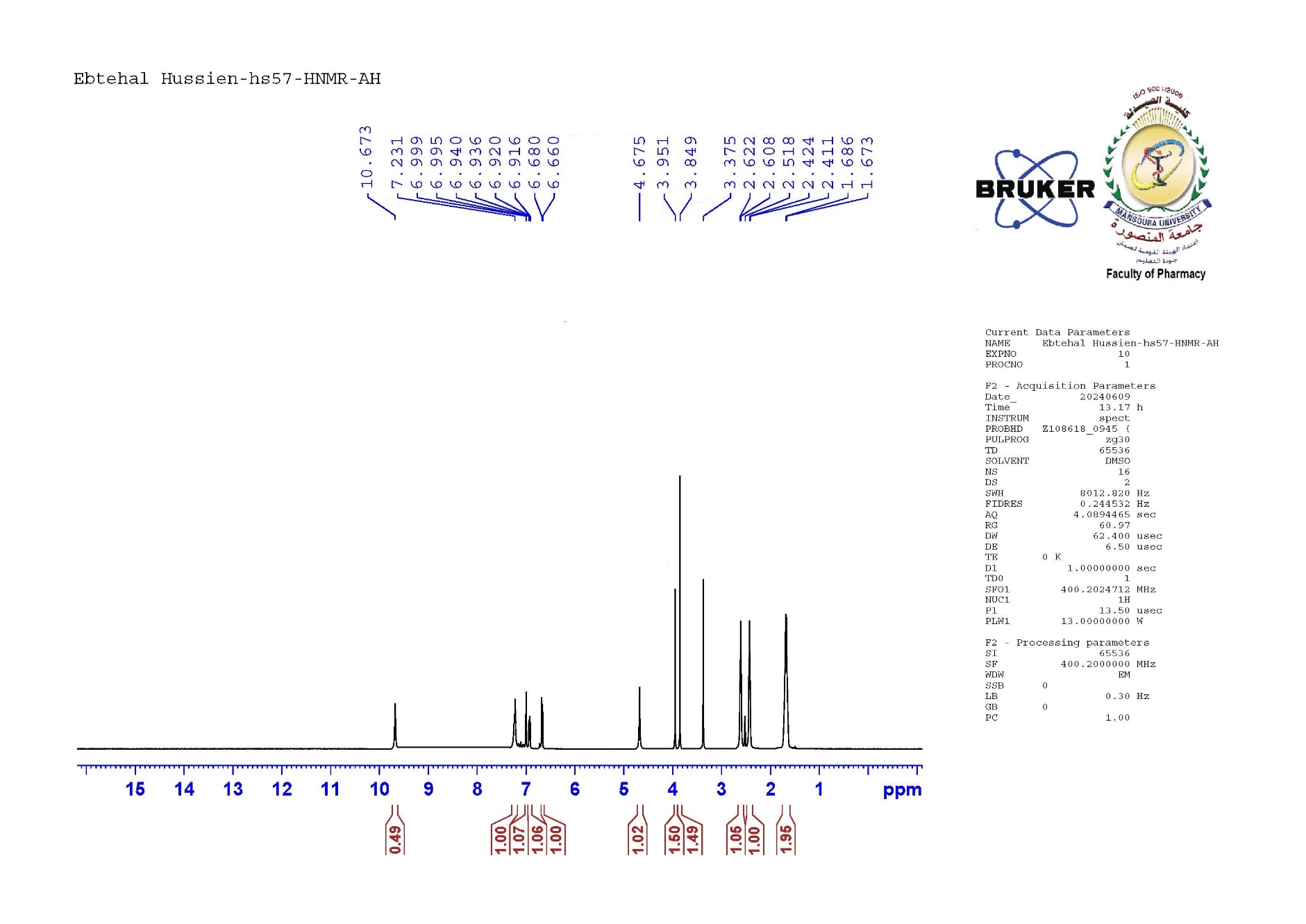

# ^1^H NMR spectrum of 5 (D_2_O)


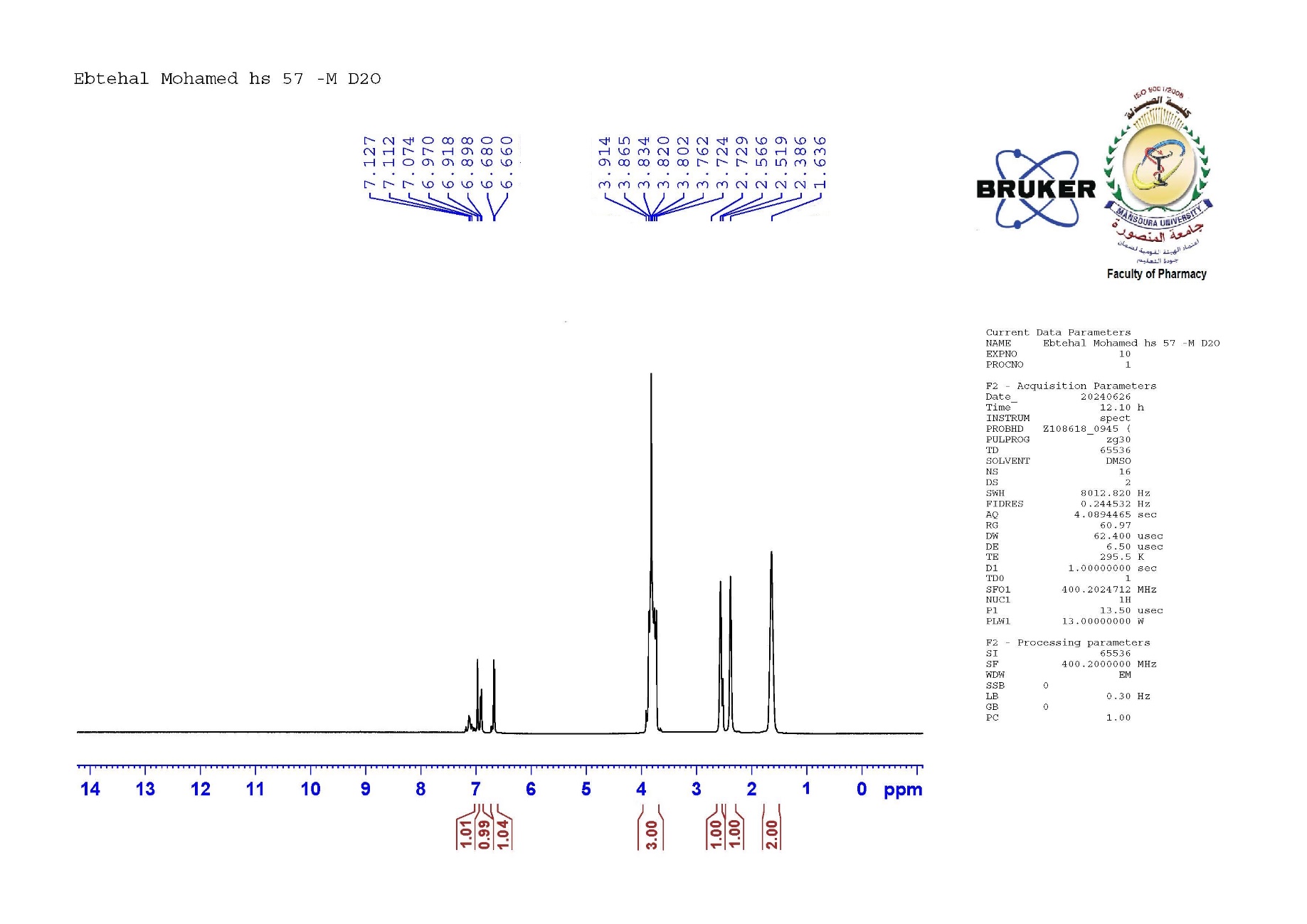

# ^13^C NMR spectrum of 5


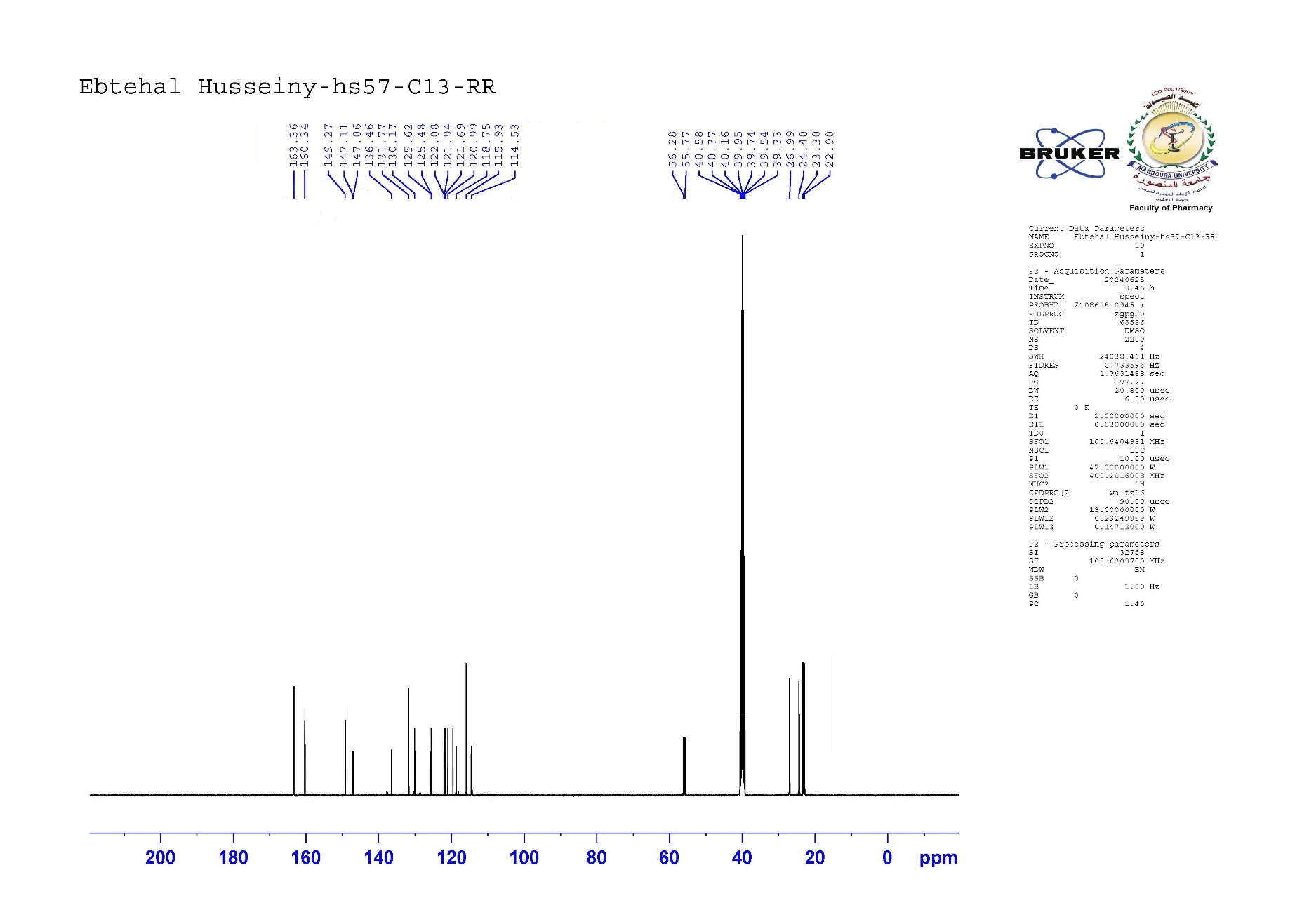

# Mass Spectrum of 5


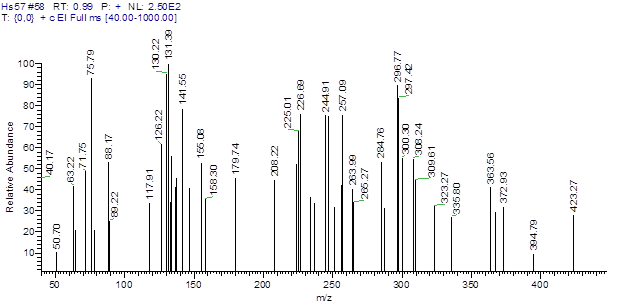

# Spectral data of compound 6

# IR spectrum of 6


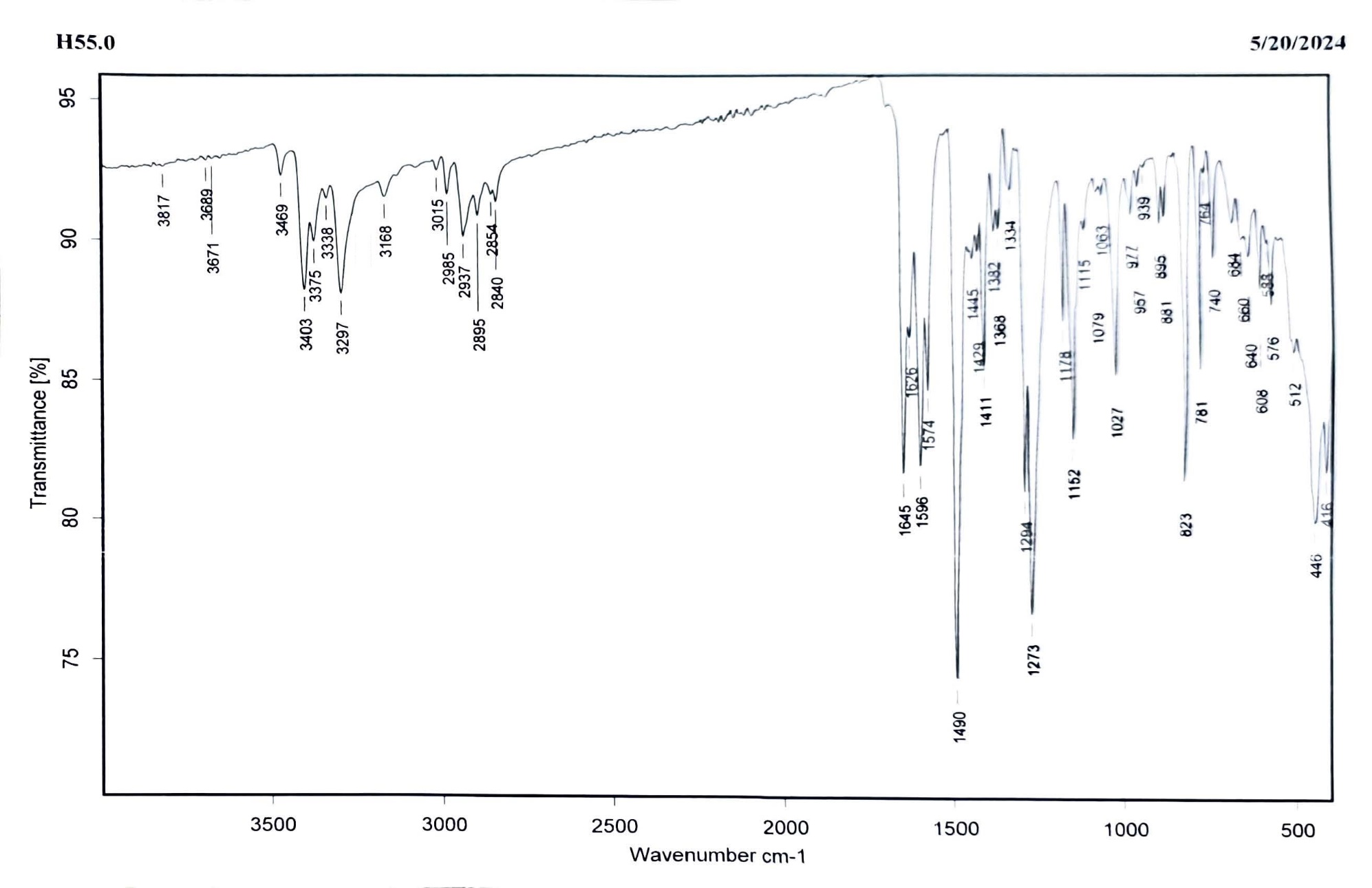

# ^1^H NMR spectrum of 6

#
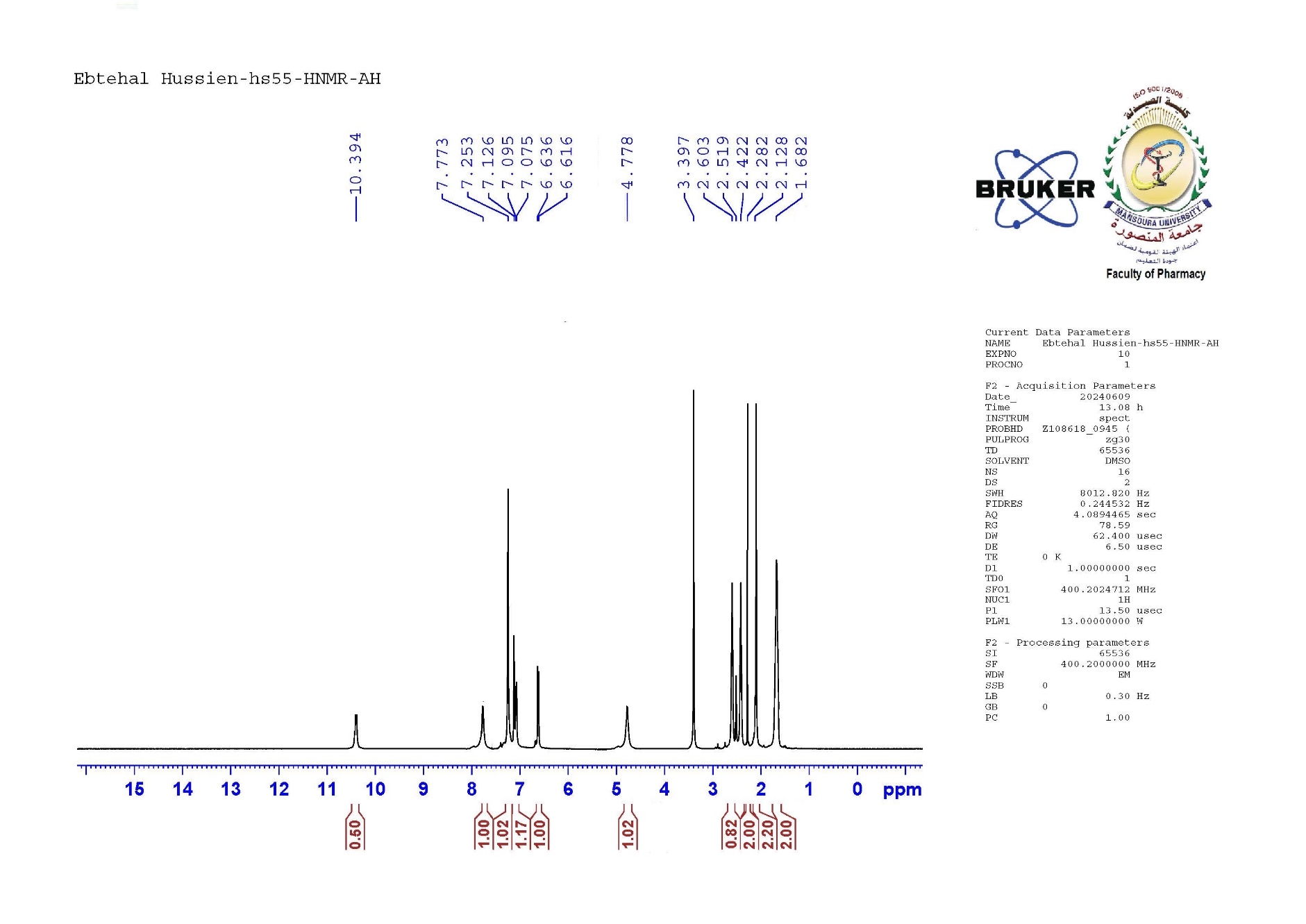

# ^1^H NMR spectrum of 6 (D_2_O)

#
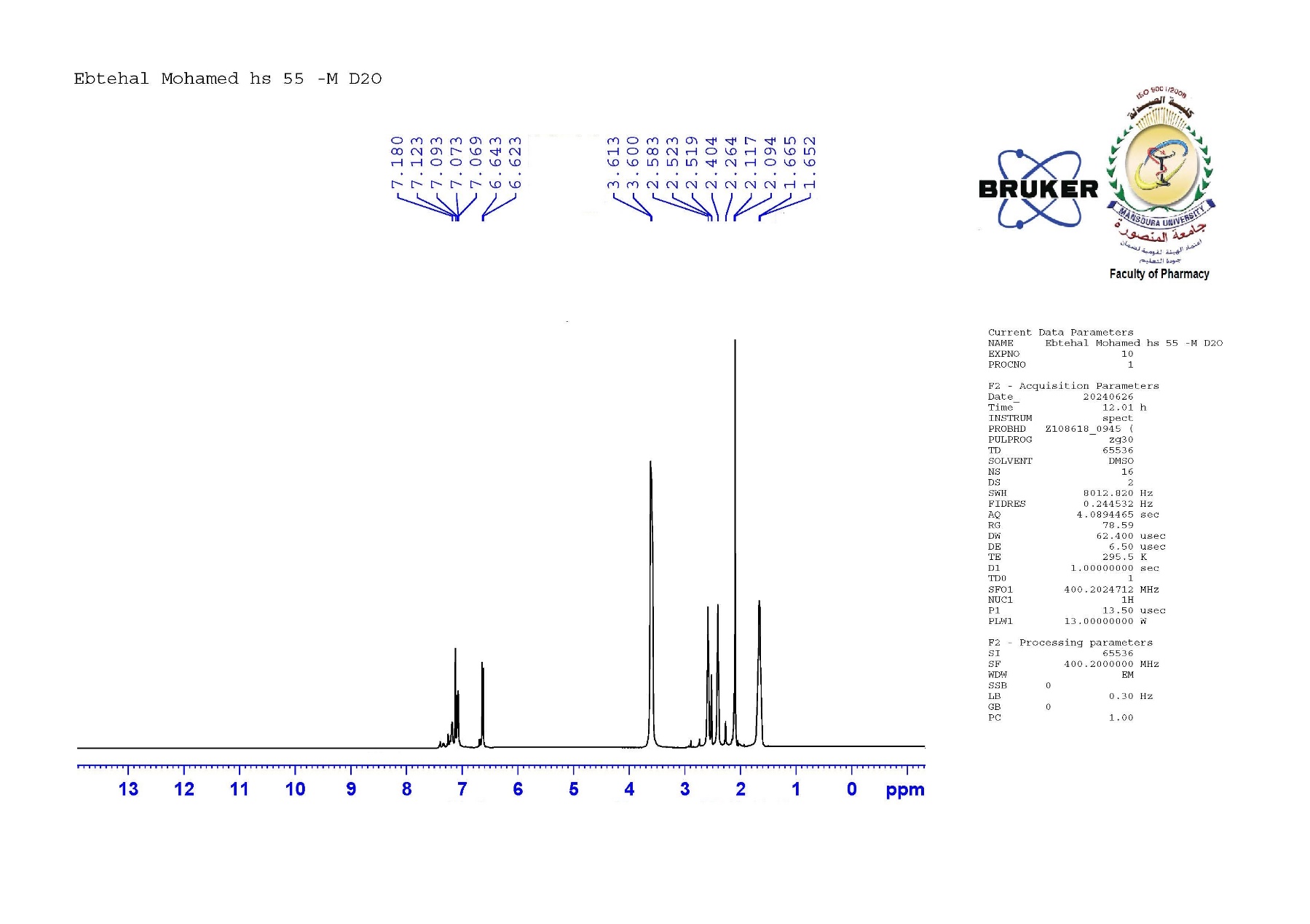

# ^13^C NMR spectrum of 6

#
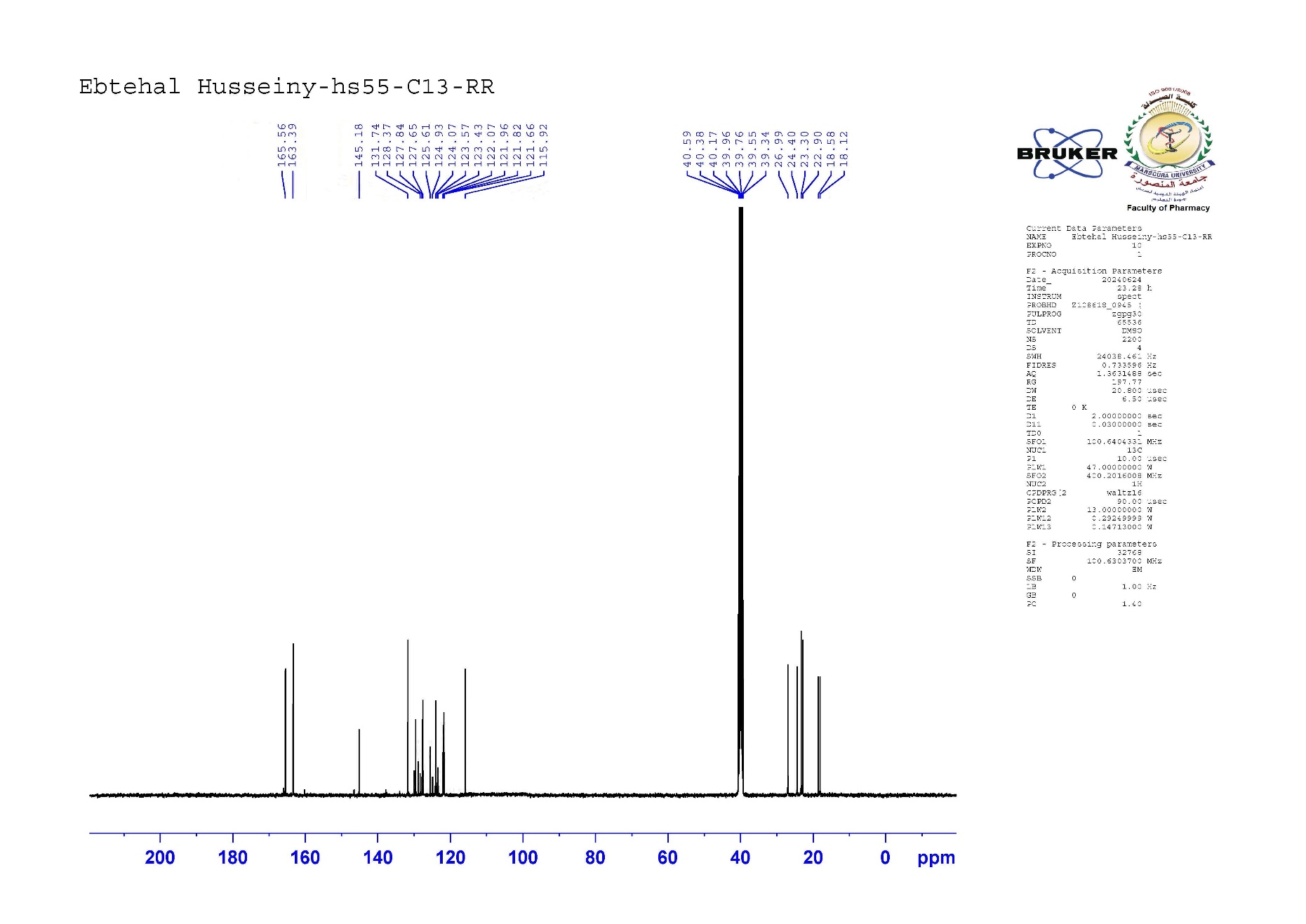

# Mass Spectrum of 6


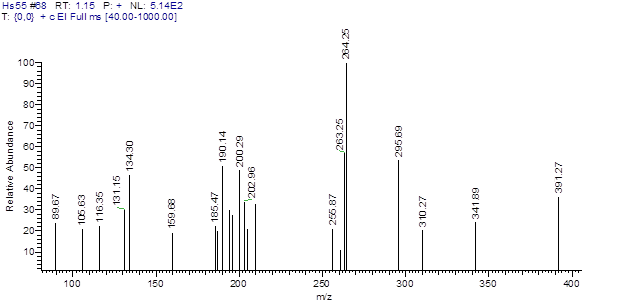

# Spectral data of compound 7

# IR spectrum of 7
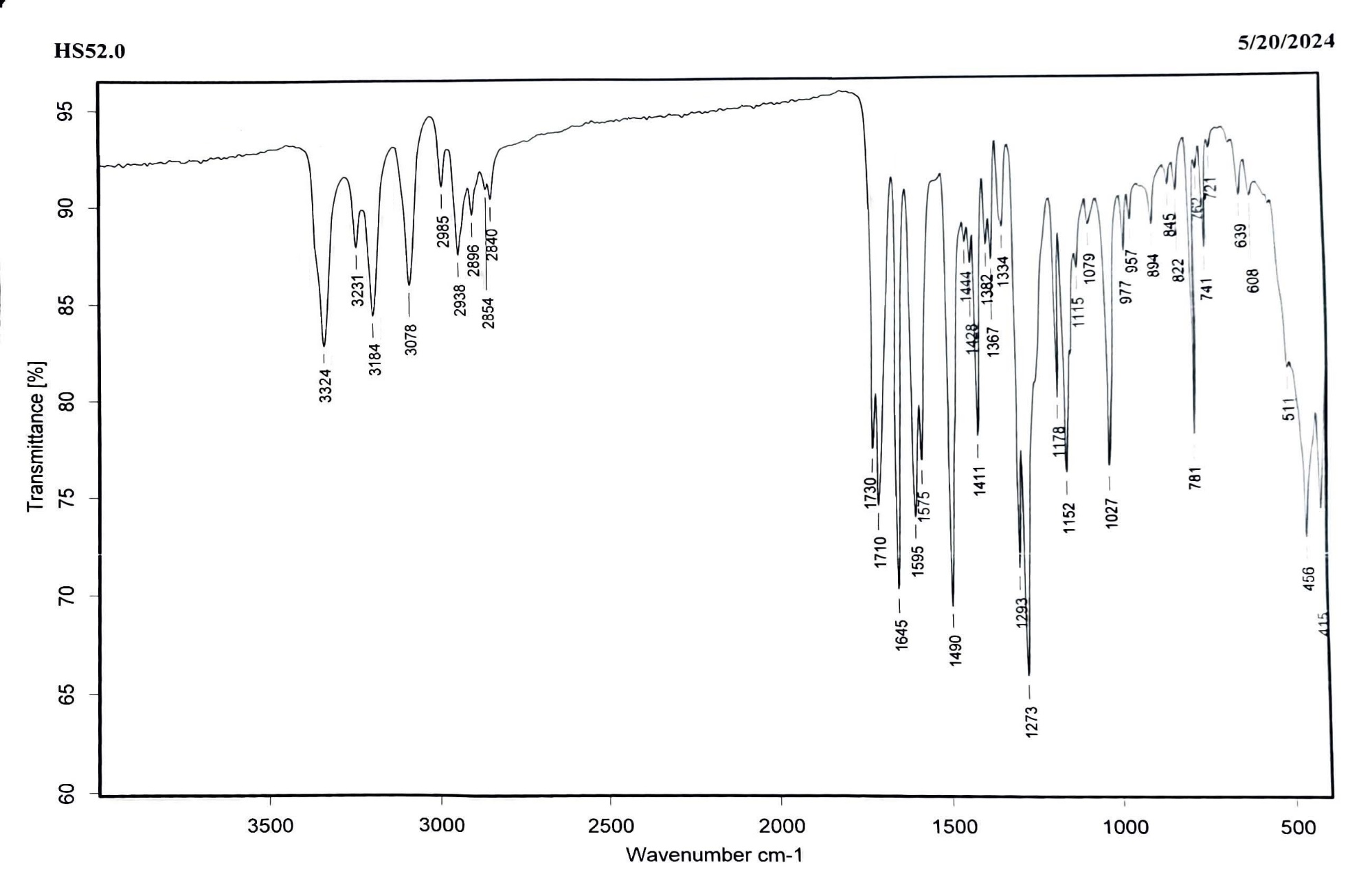

# ^1^H NMR spectrum of 7


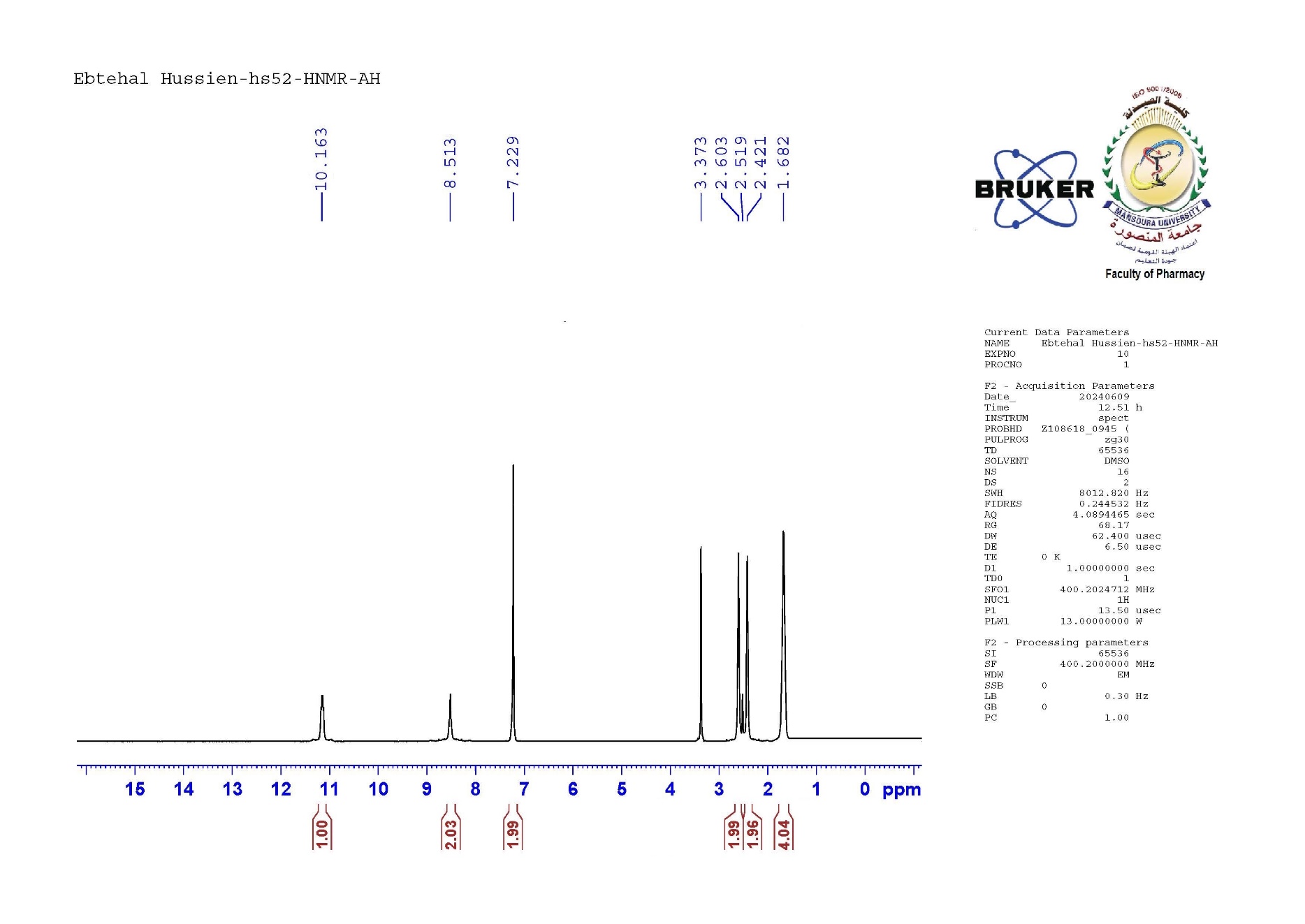

# ^1^H NMR spectrum of 7 (D_2_O)


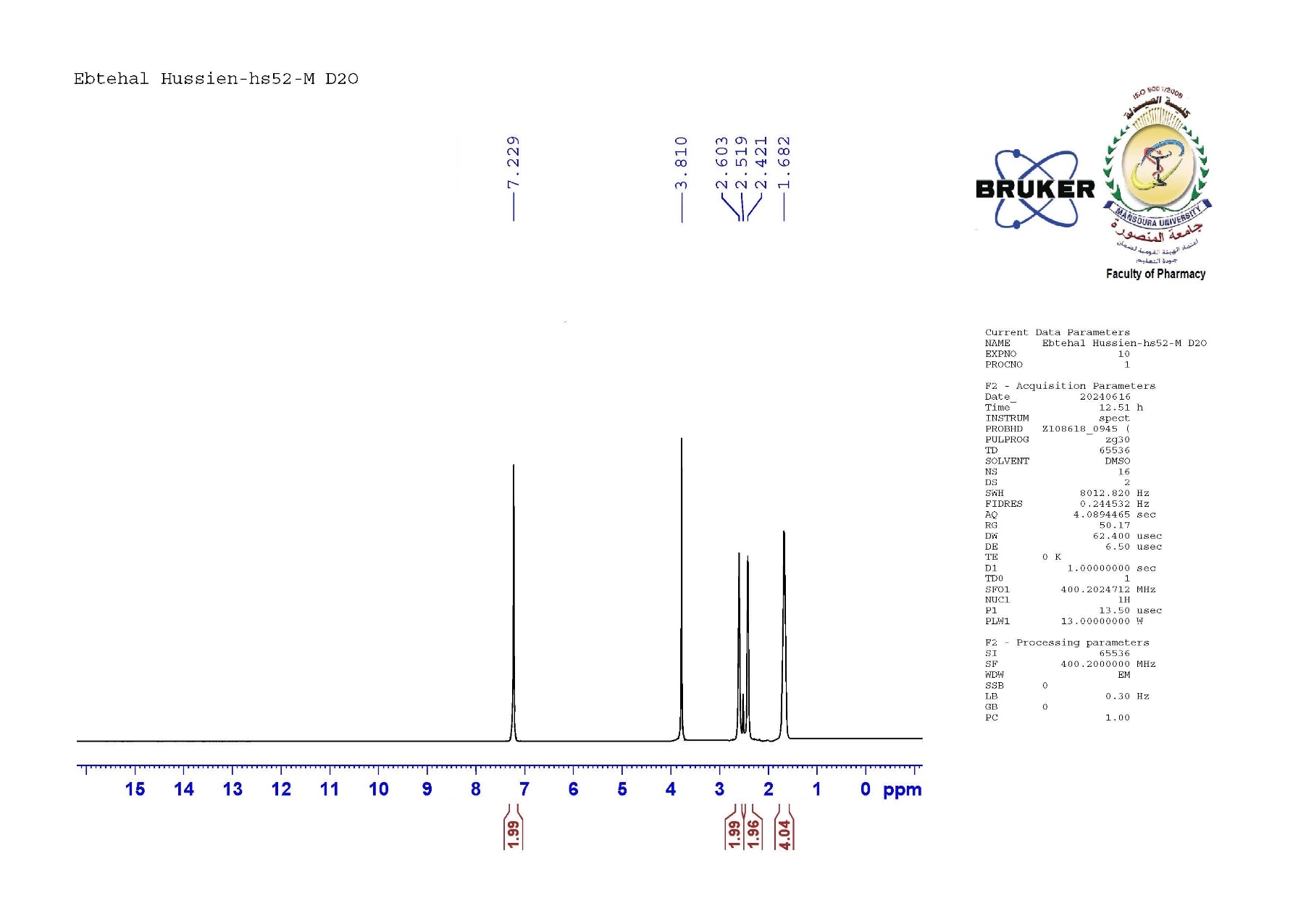

# ^13^C NMR spectrum of 7


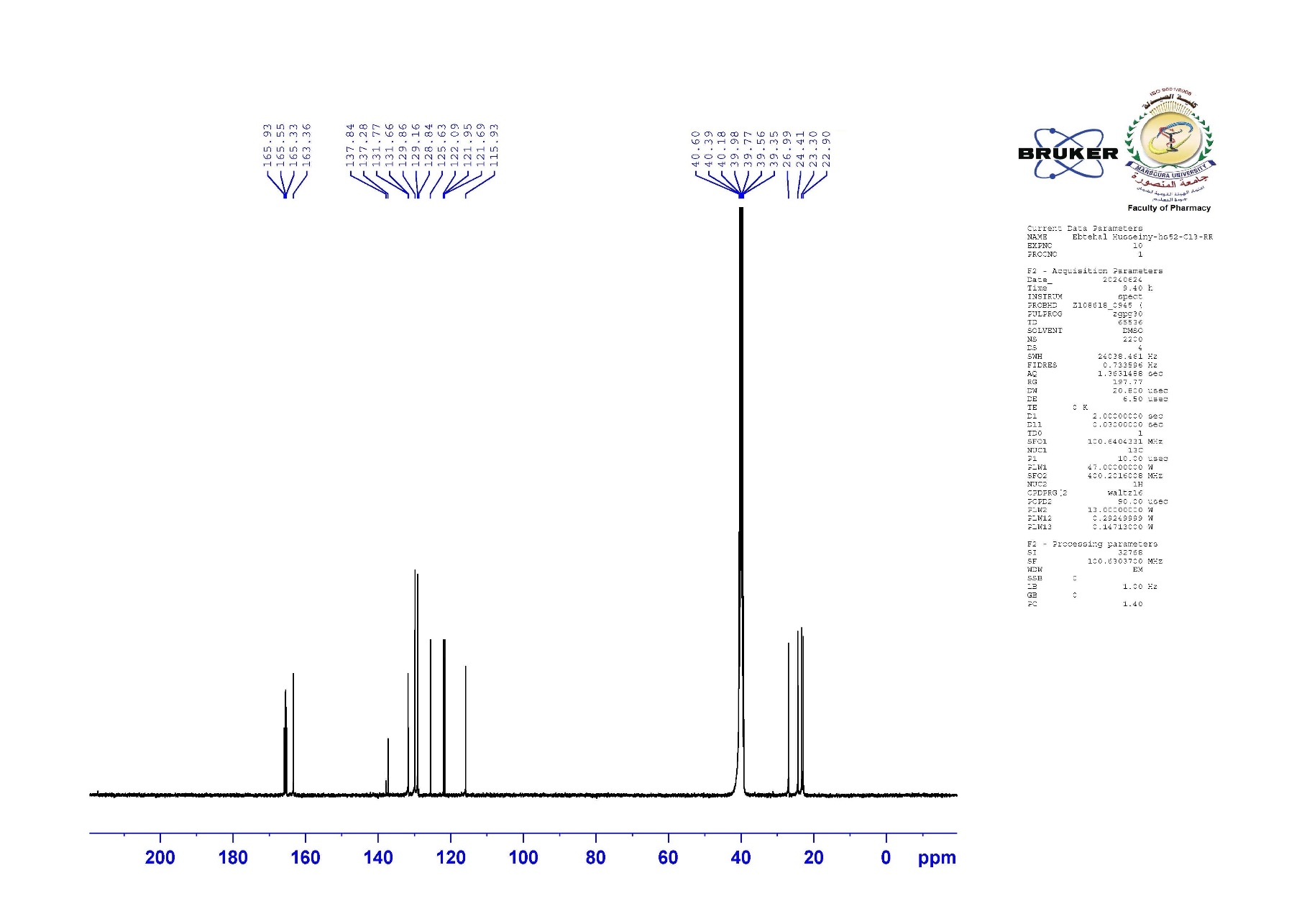

# Mass Spectrum of 7


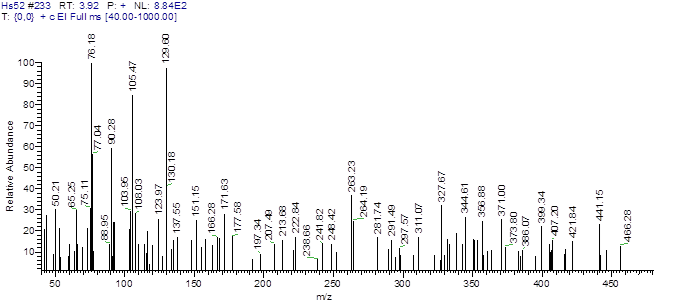

# Spectral data of compound 8

# ^1^H NMR spectrum of 8


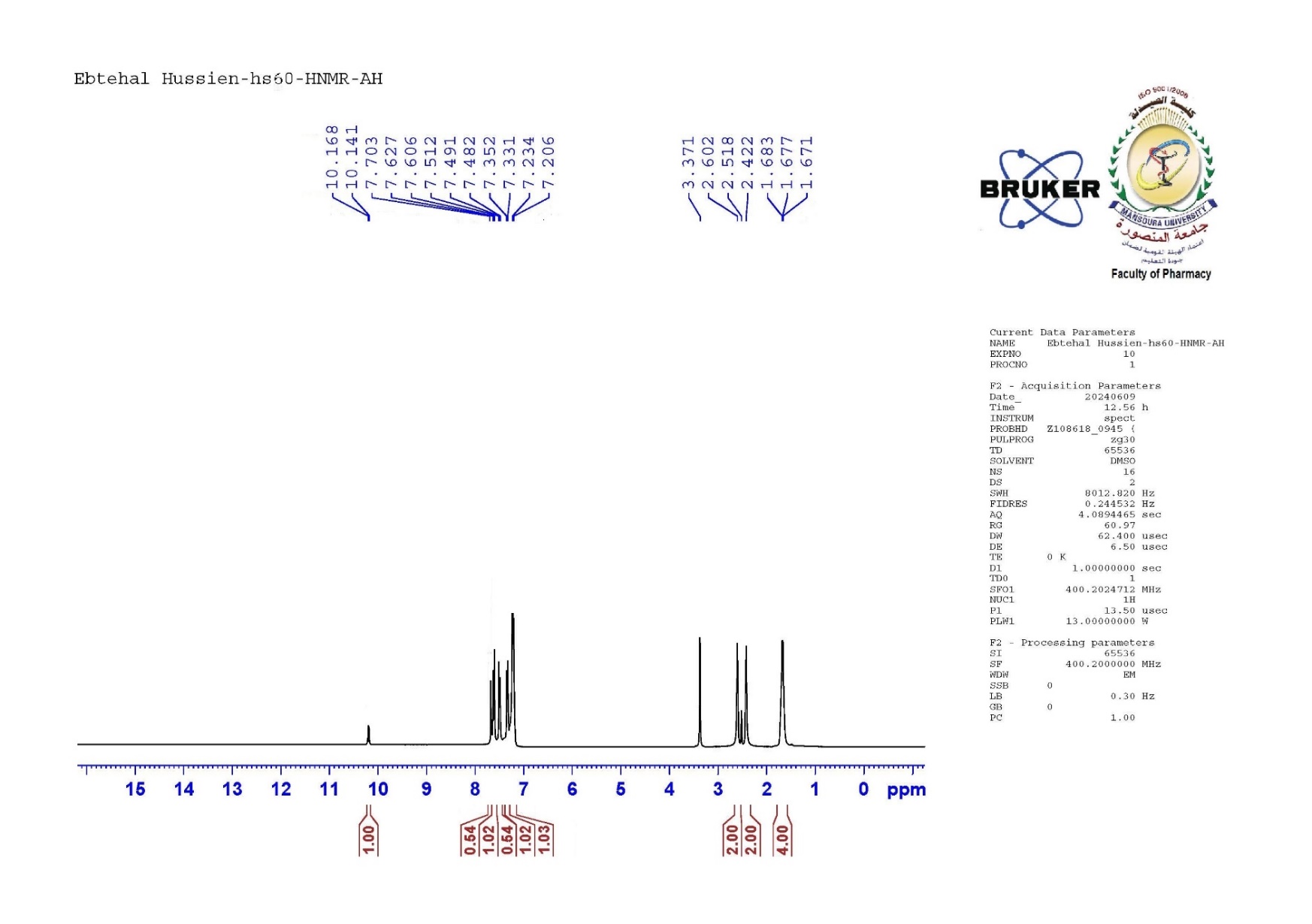

# ^1^H NMR spectrum of 8 (D_2_O)


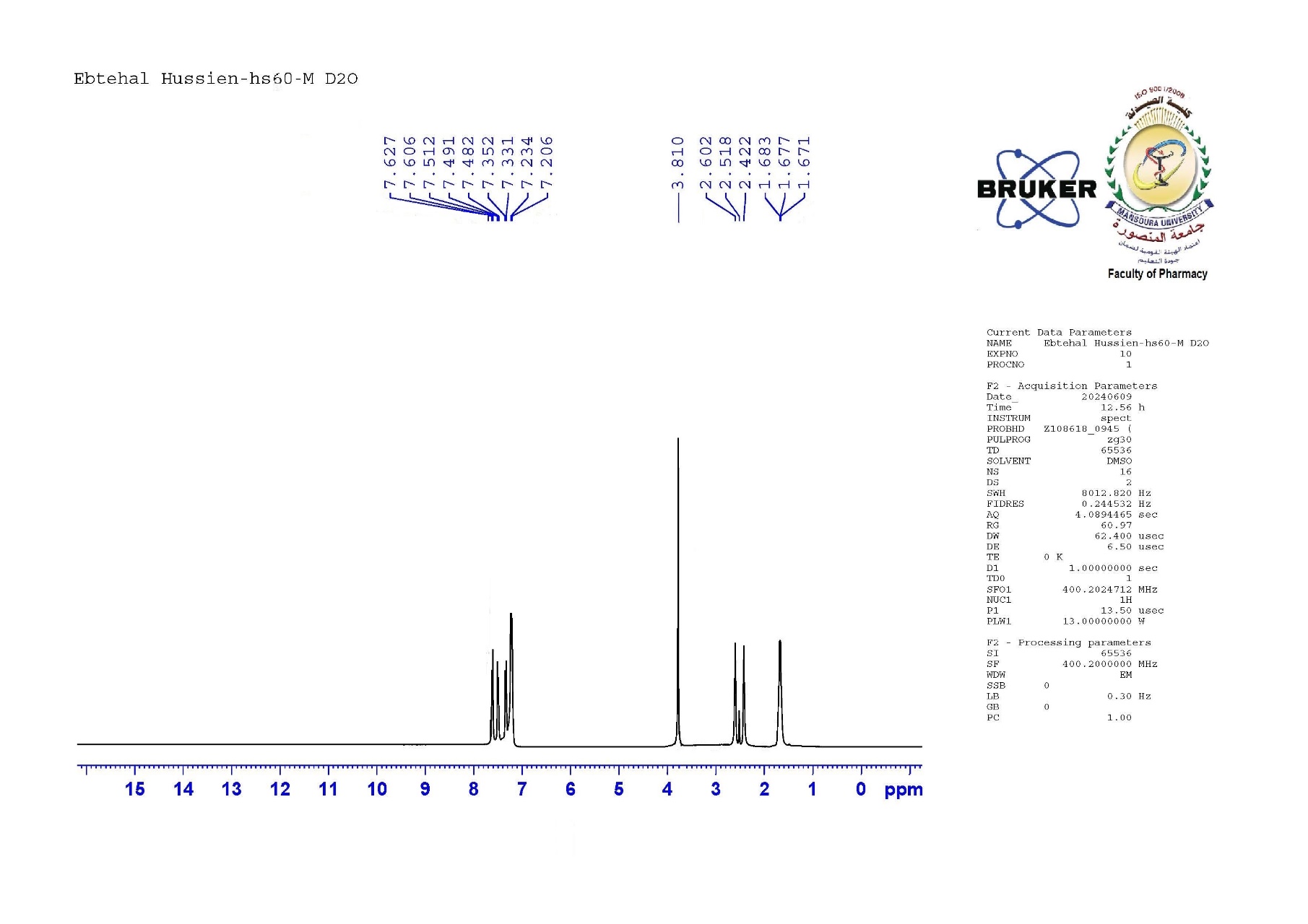

# ^13^C NMR spectrum of 8


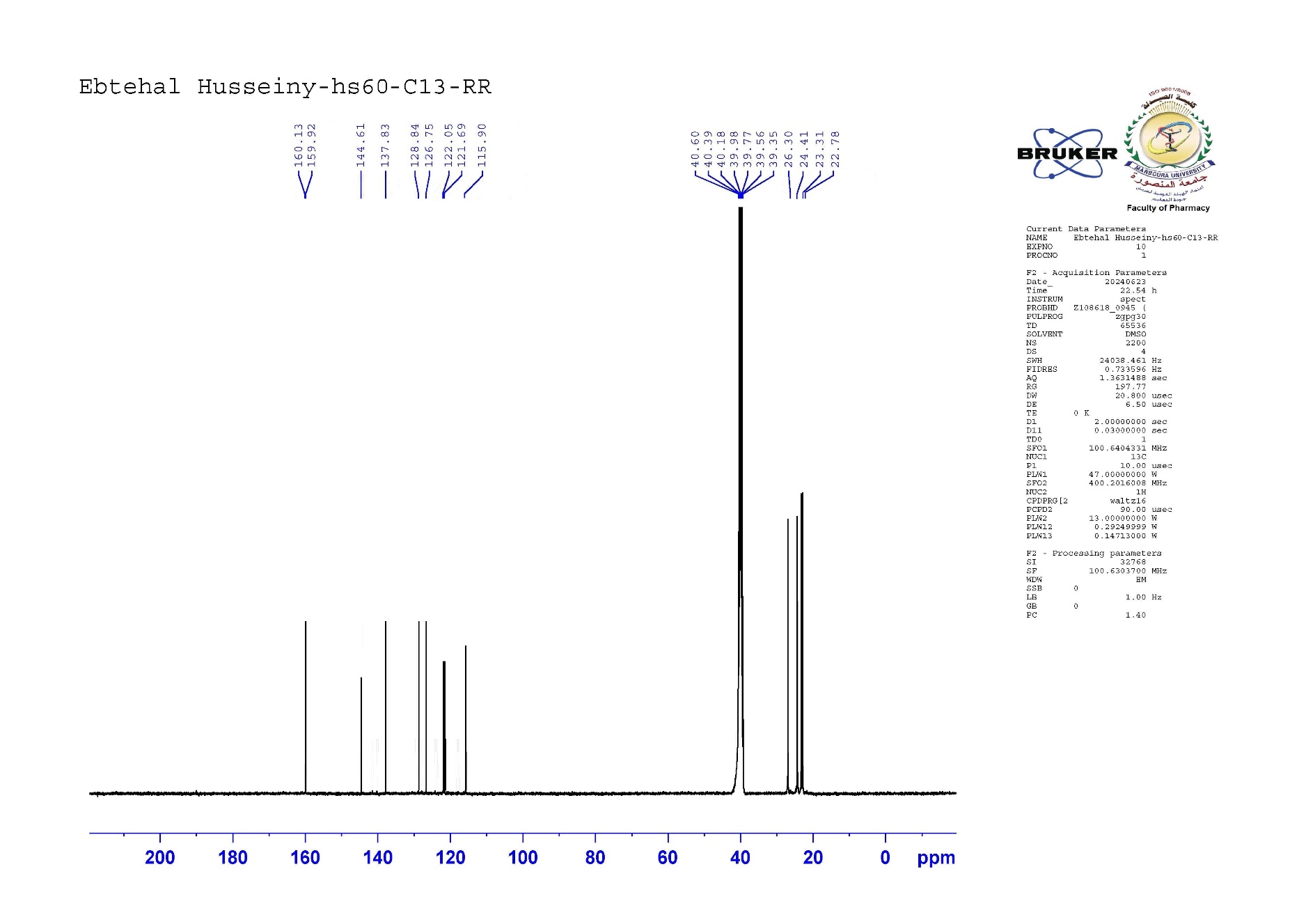

# Mass Spectrum of 8

#
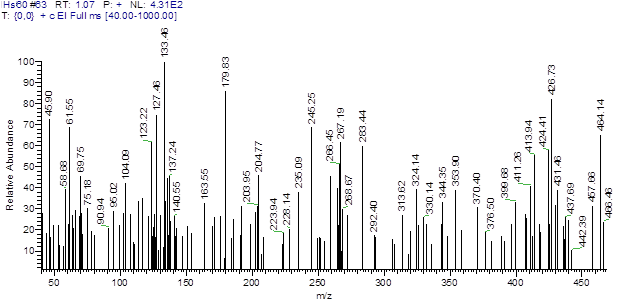


# Spectral data of compound 9

# IR spectrum of 9

#
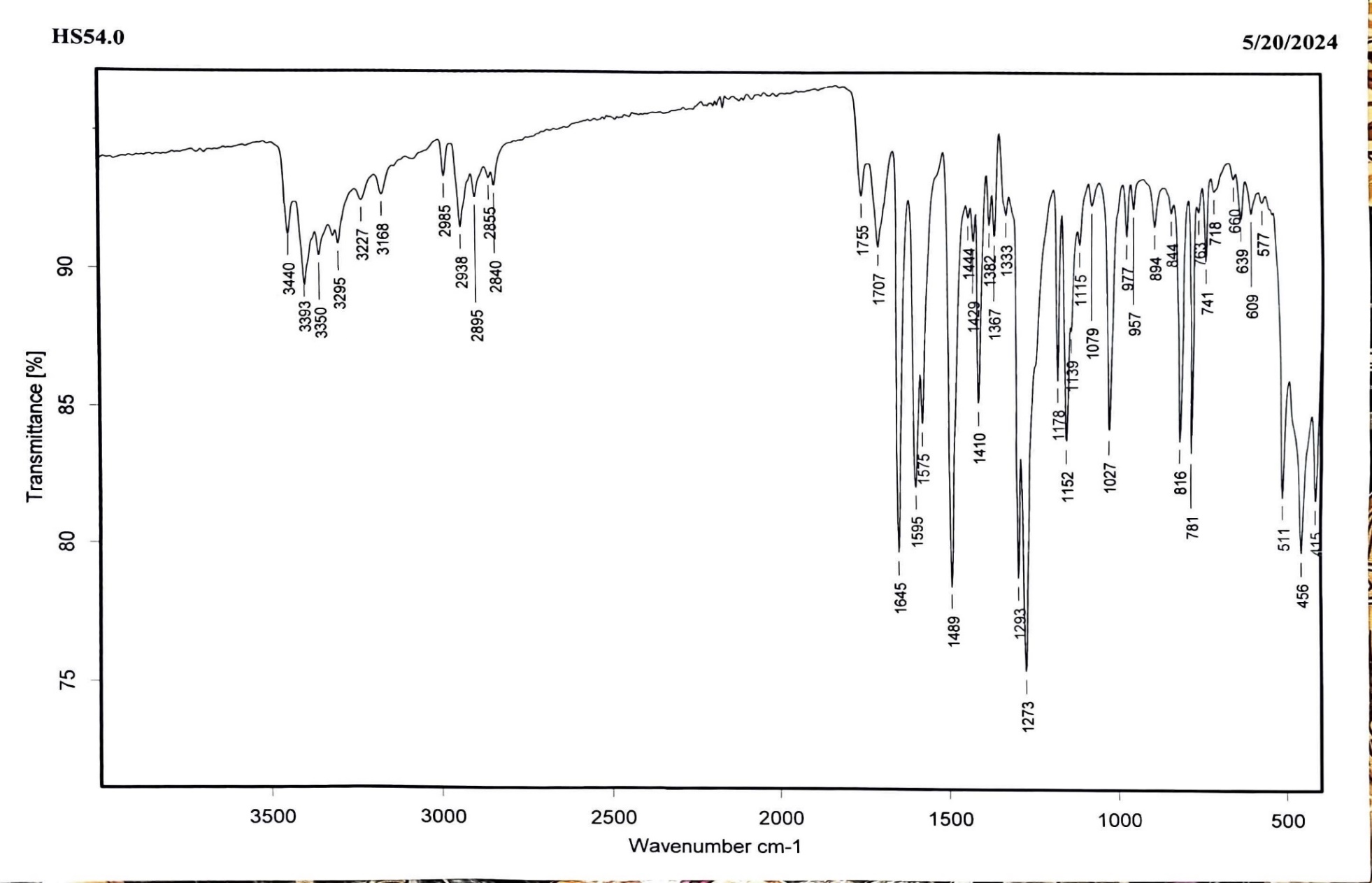

# ^1^H NMR spectrum of 9


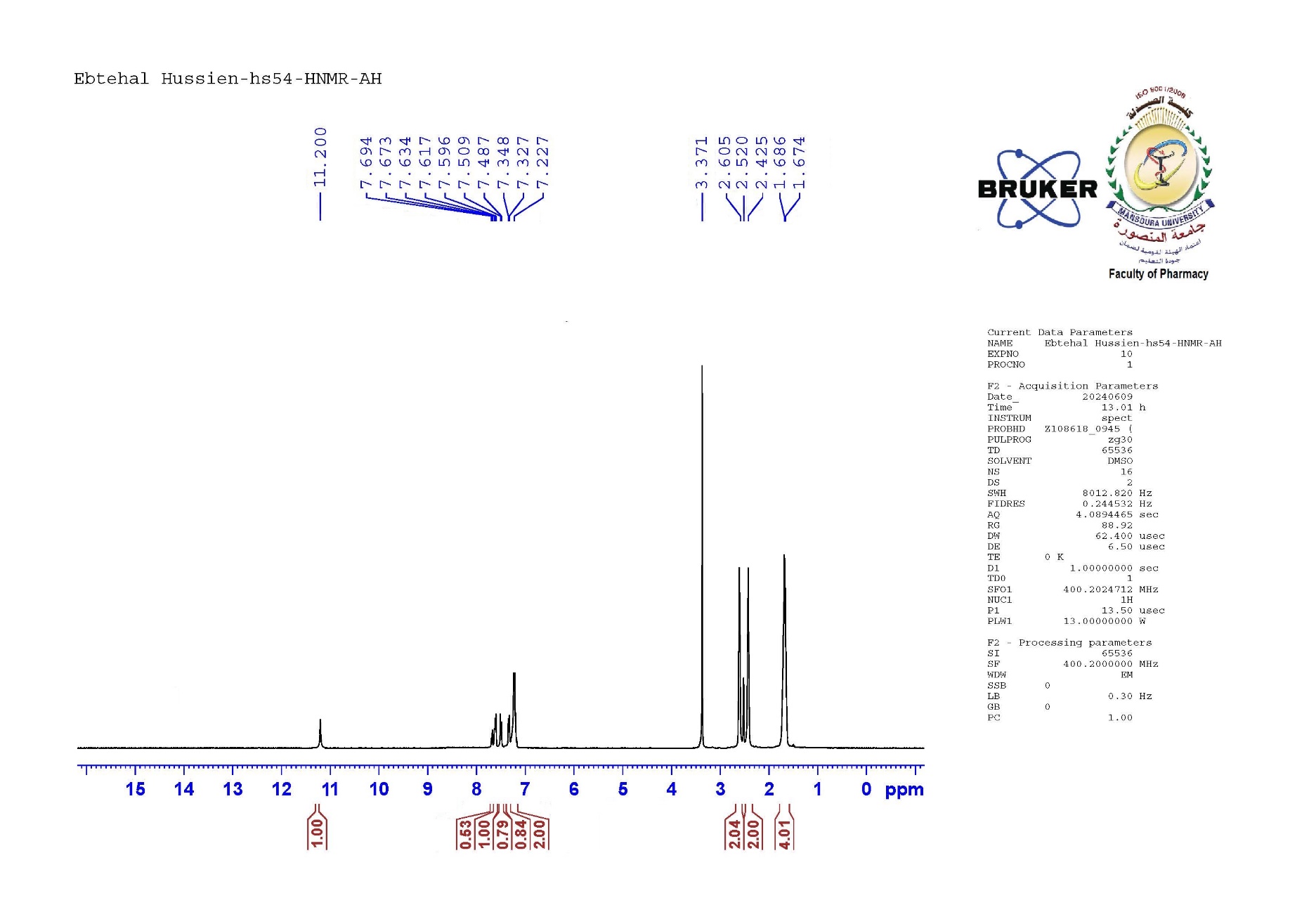

# ^1^H NMR spectrum of 9 (D_2_O)
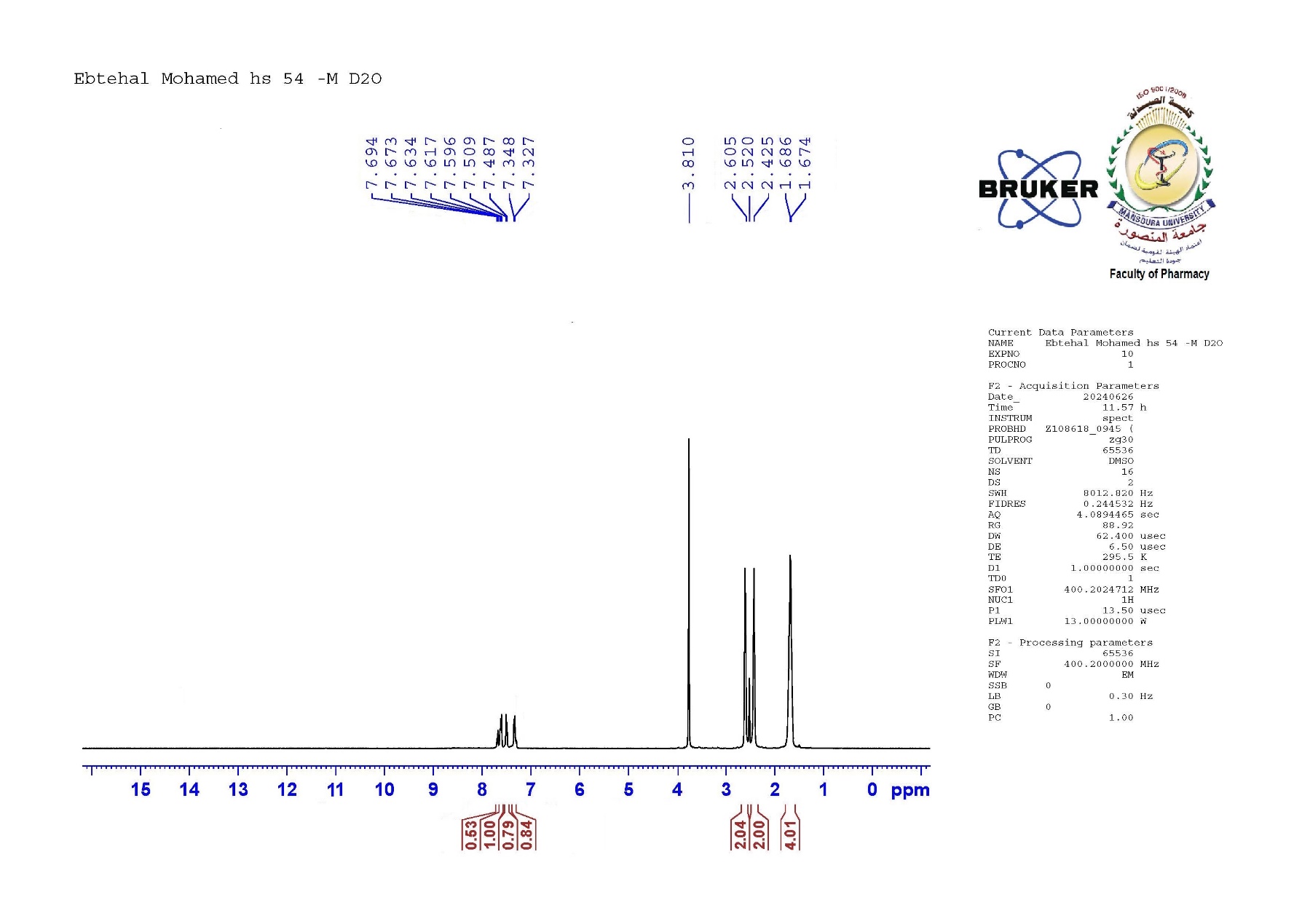

# ^13^C NMR spectrum of 9

#
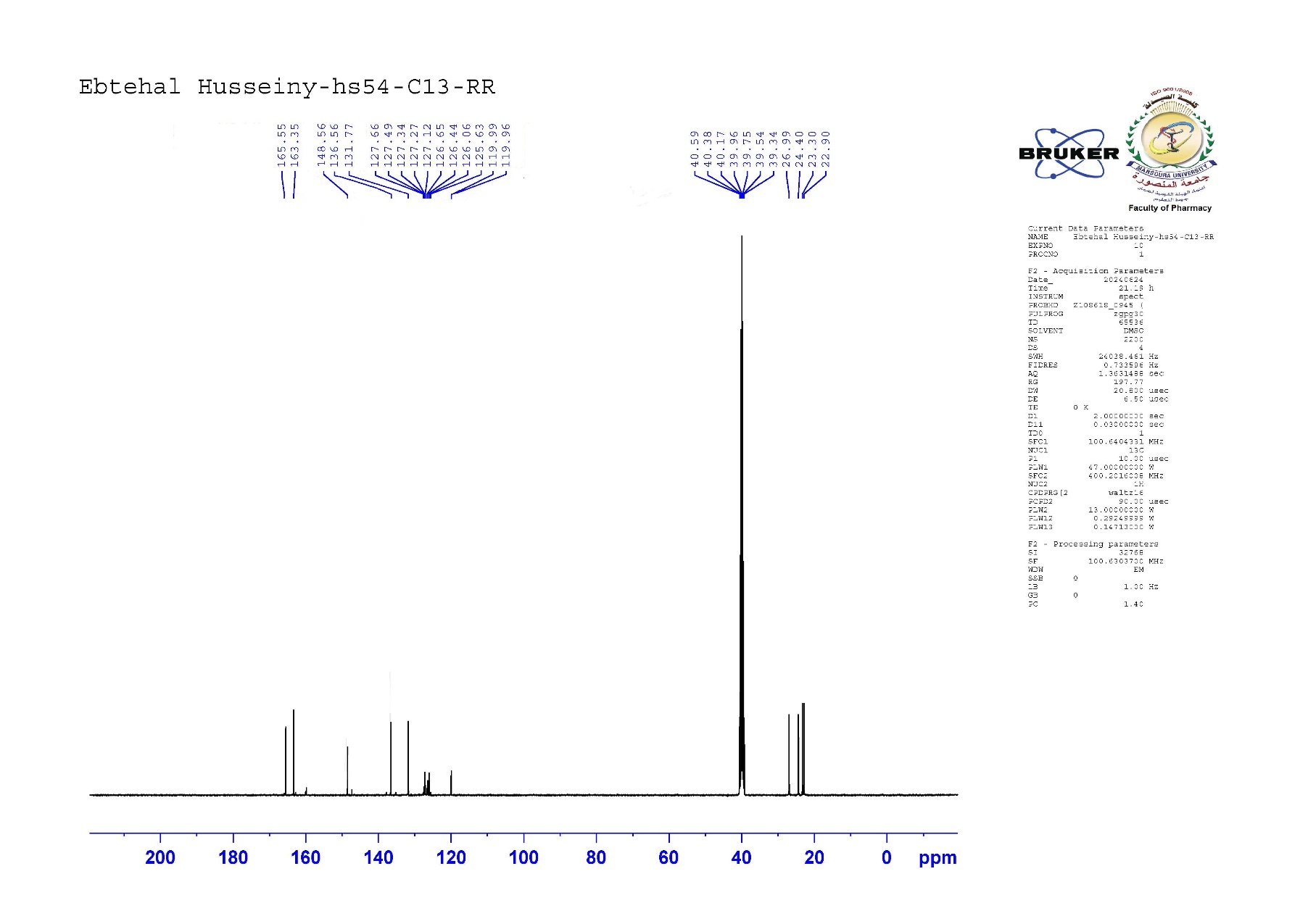

# Mass Spectrum of 9

#
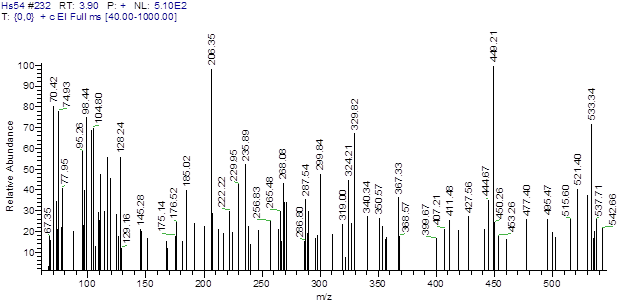


# Spectral data of compound 10

# IR spectrum of 10
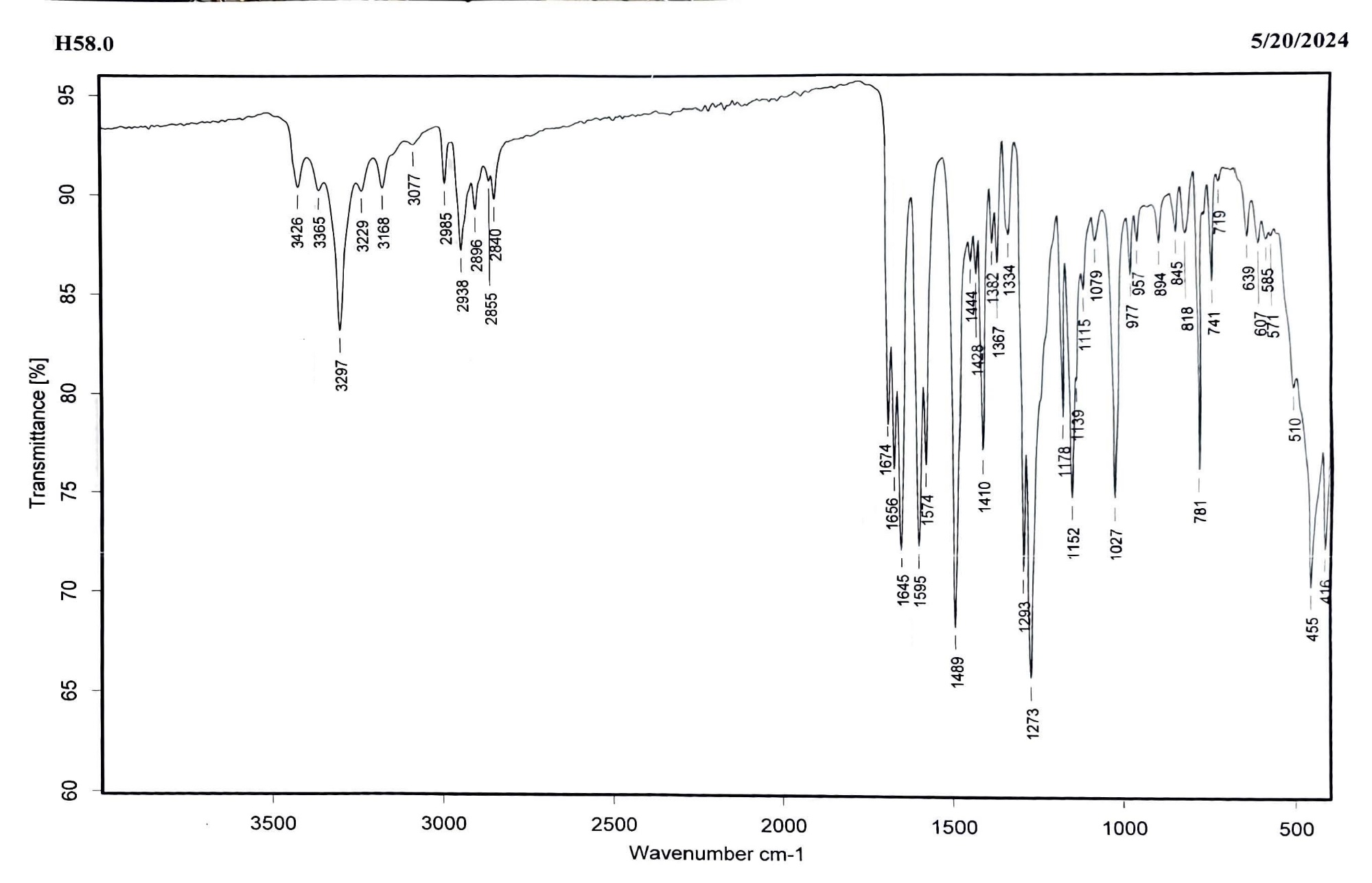

# ^1^H NMR spectrum of 10
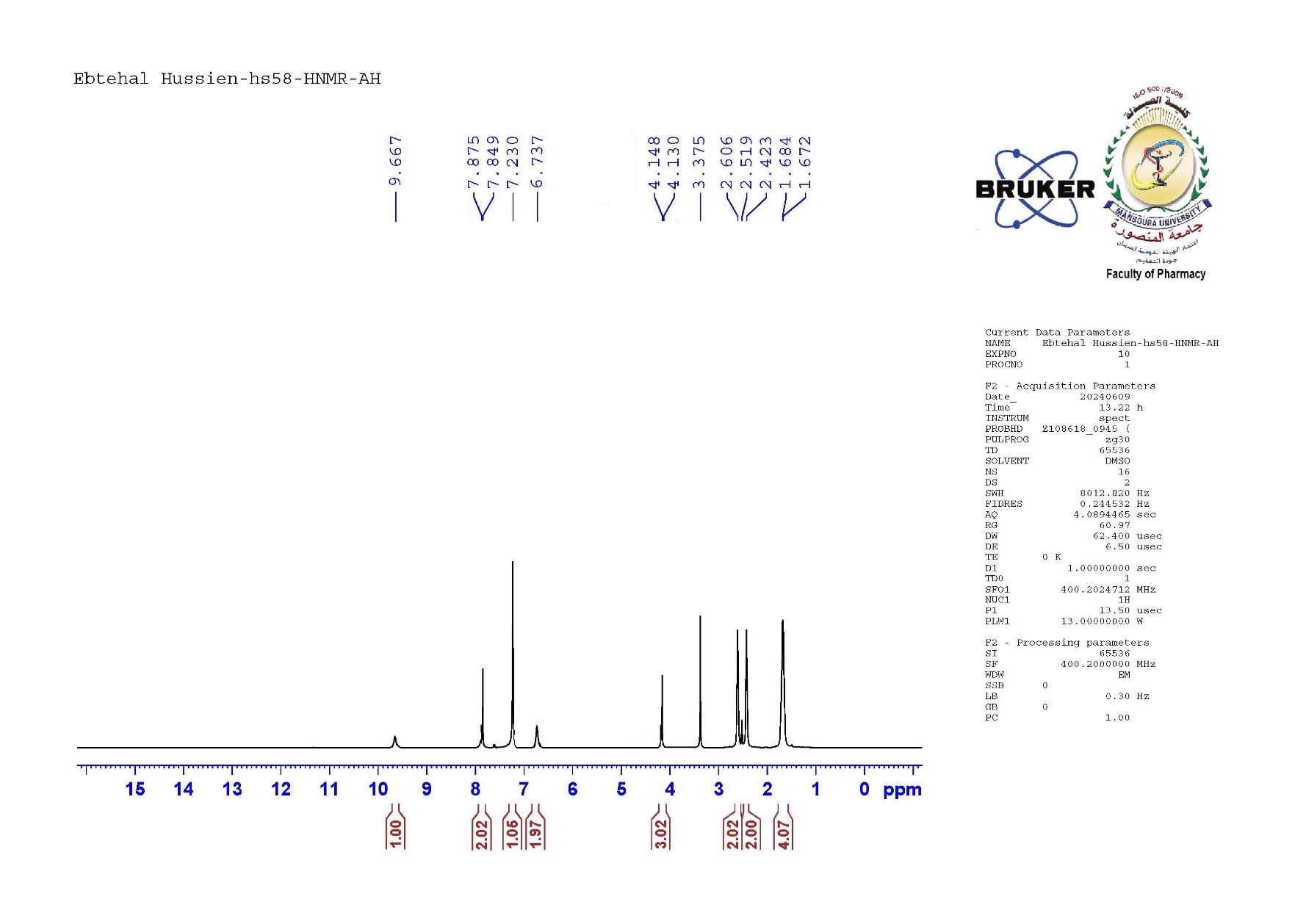

# ^13^C NMR spectrum of 10
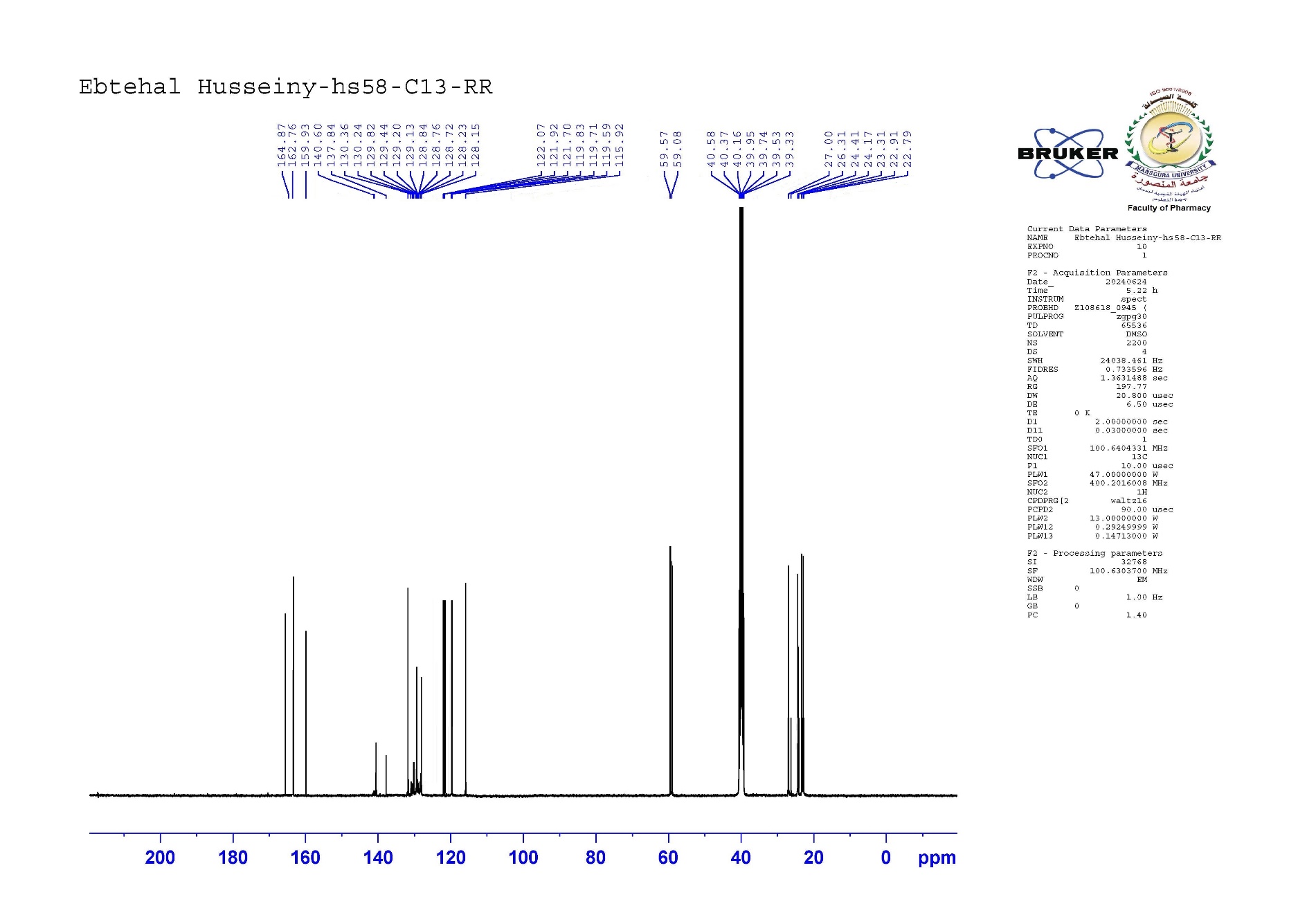

# Mass Spectrum of 10

#
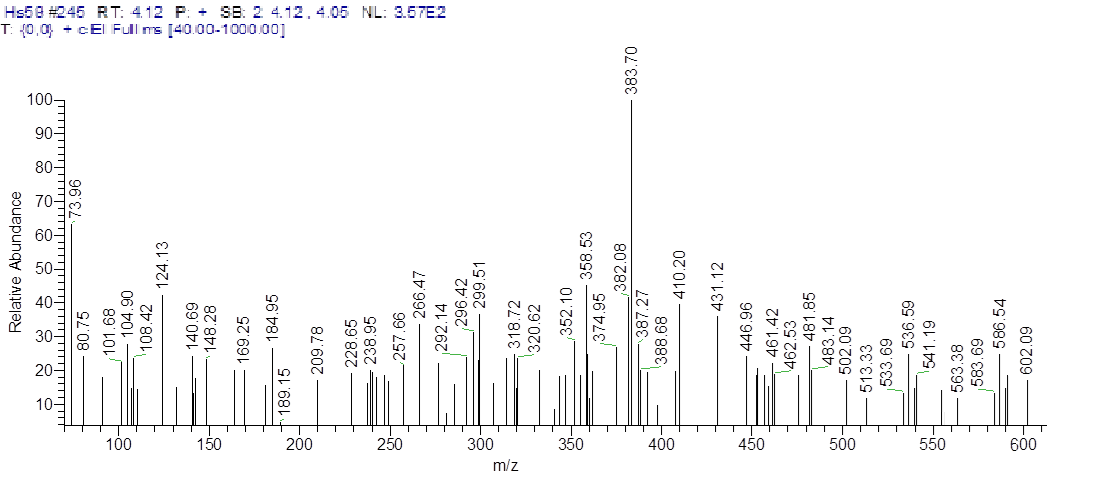


# Spectral data of compound 11

# IR spectrum of 11
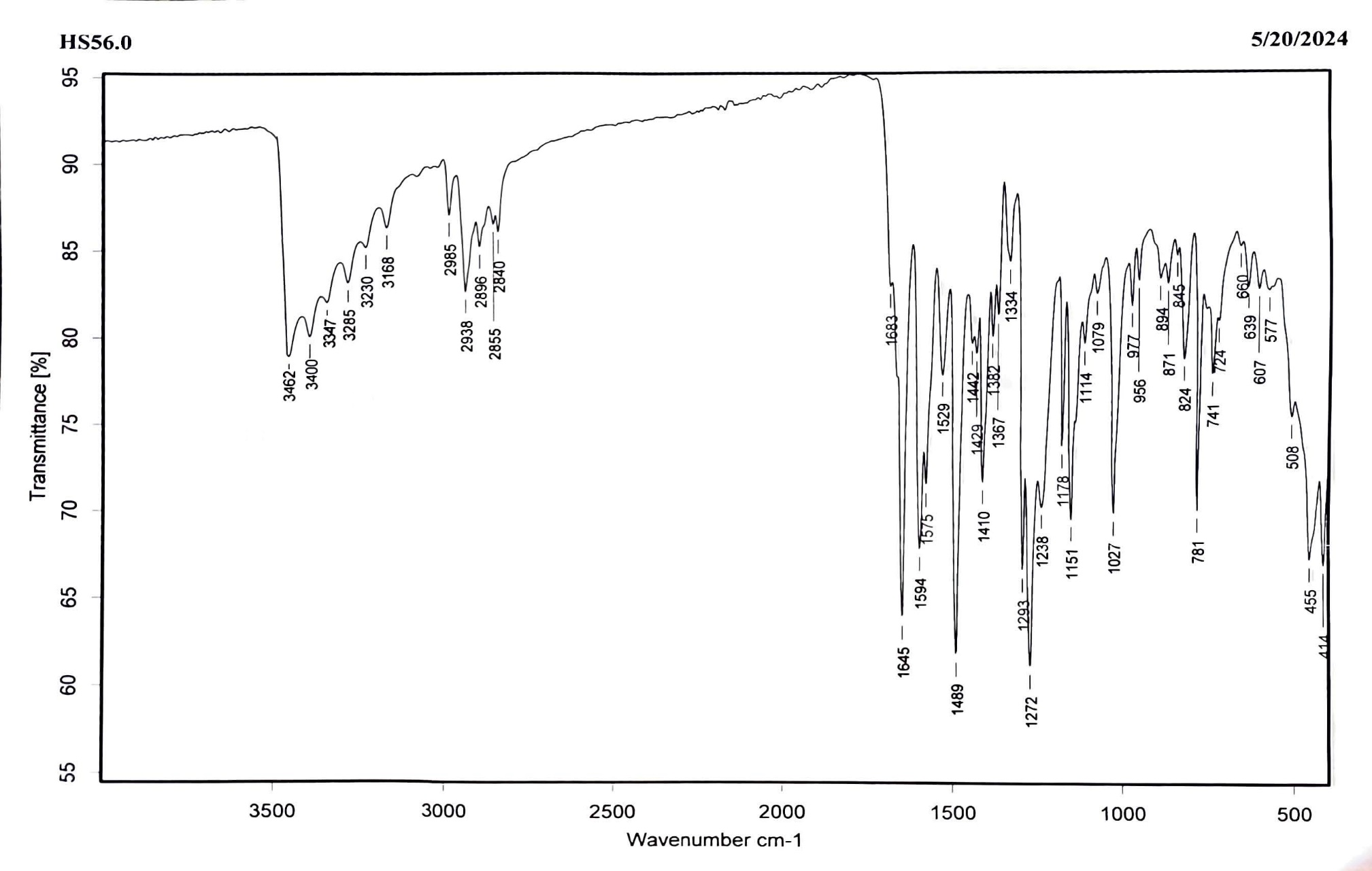

# ^1^H NMR spectrum of 11
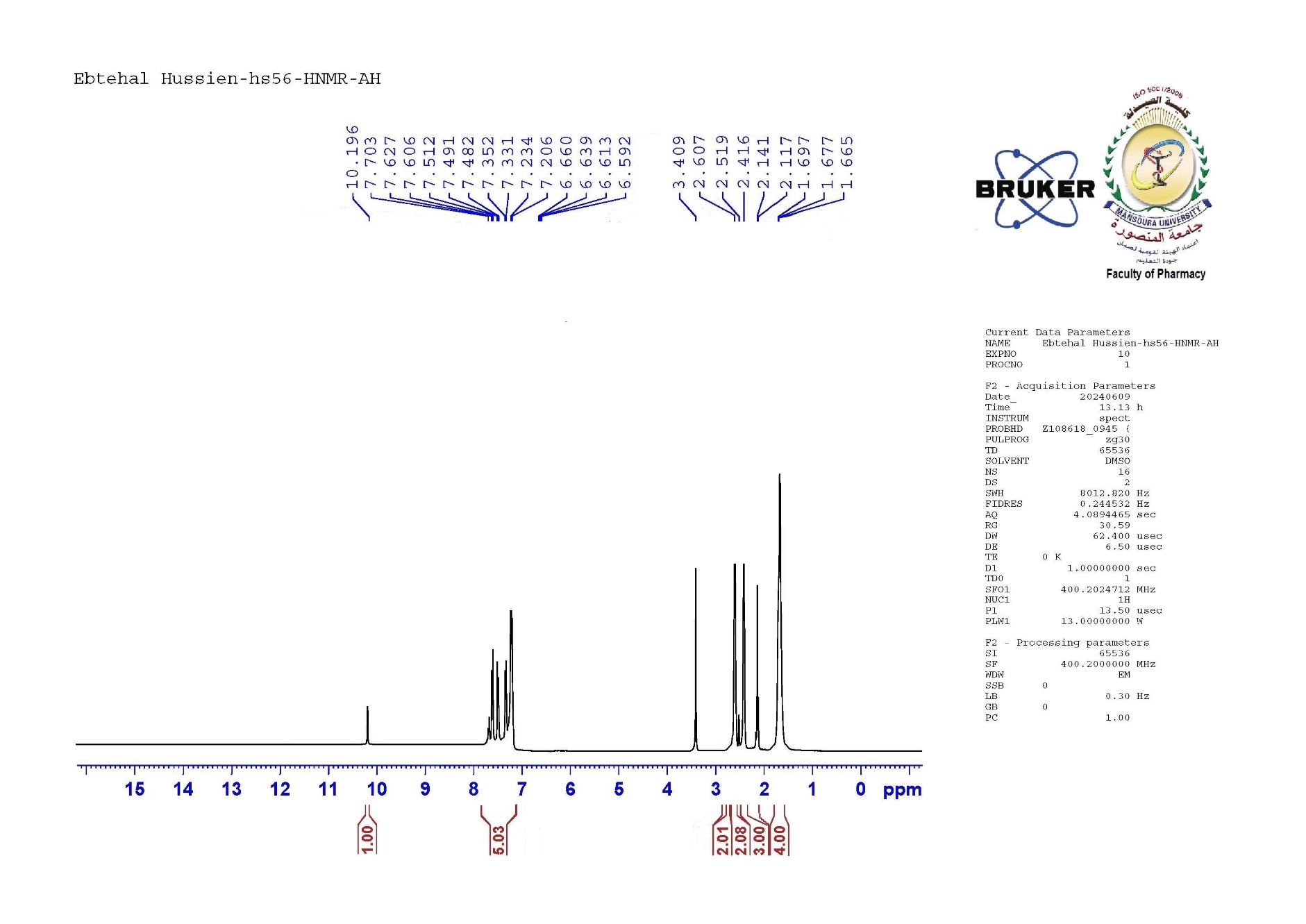

# ^1^H NMR spectrum of 11 (D_2_O)
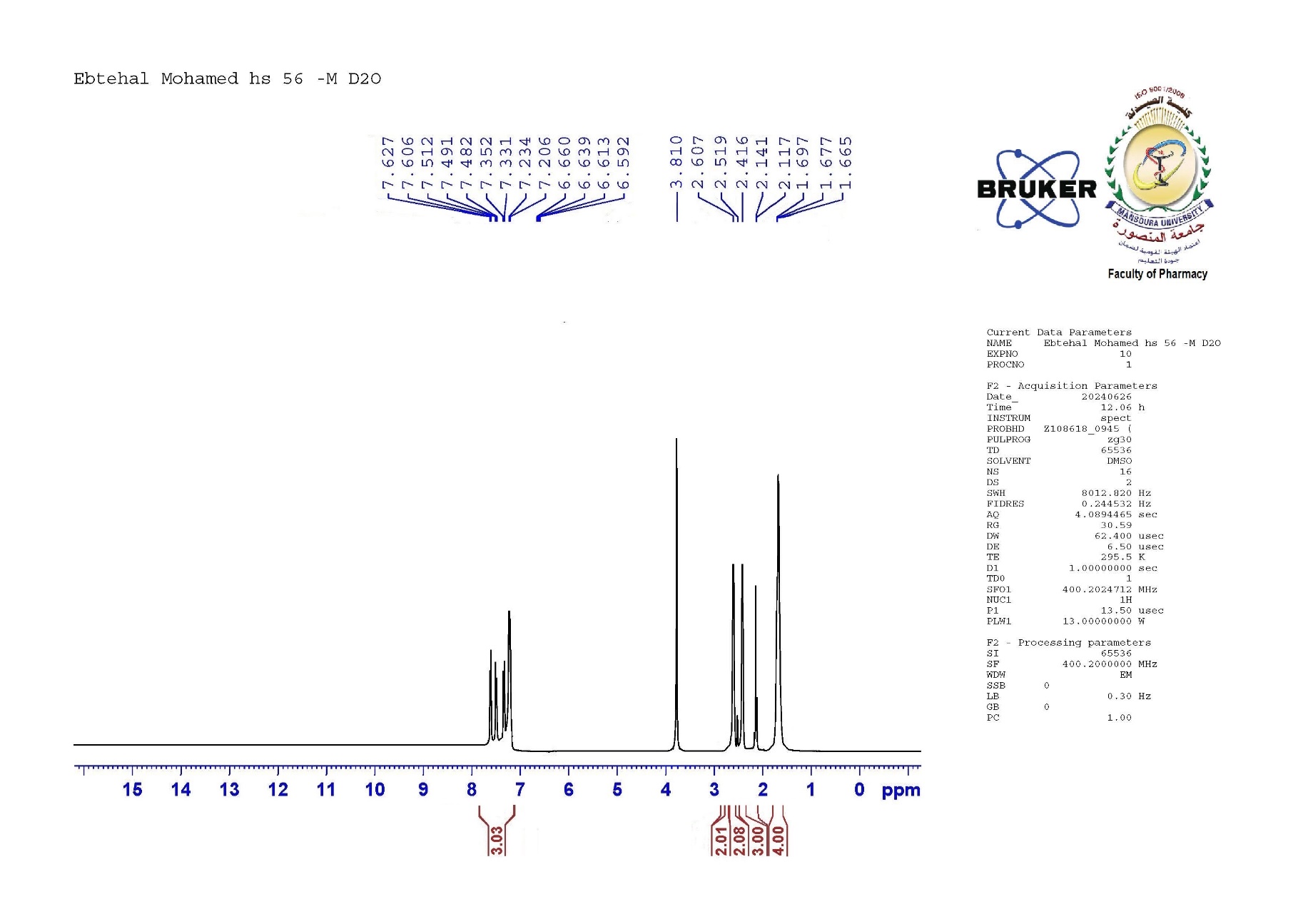

# ^13^C NMR spectrum of 11
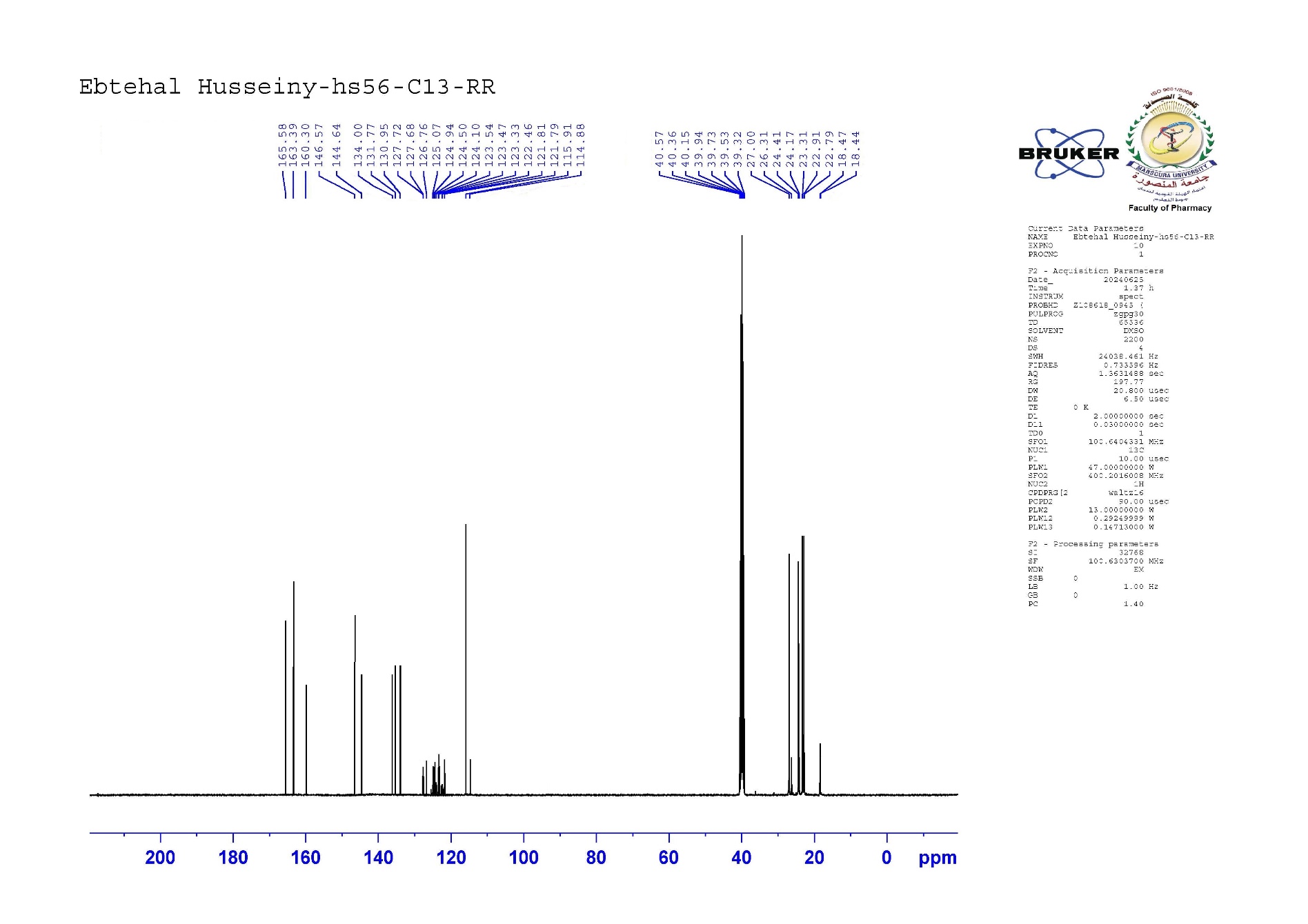

# Mass Spectrum of 11

#
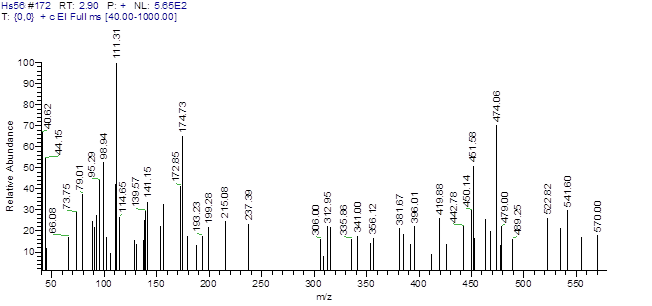


#
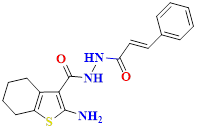


# Specifications of instruments used for characterization of new compounds

Commercially available starting materials, reagents, and solvents were used as supplied without further purification. All melting points are uncorrected and determined by the Stuart "SMP3" electrical digital melting point apparatus. Microwave reactor Anton Paar 'monowave 300' was used for microwave irradiation reactions using borosilicate glass vials of 10 mL. The infrared (IR) spectra of the "PerkinElmer-293" spectrophotometer (cm^-1^) were measured using KBr disks. ^1^H-NMR and ^13^C-NMR spectra were measured on Varian Mercury "400 MHz" spectrometer in "DMSO-d_6_" as a solvent using tetramethyl silane as an internal standard. Chemical shift (δ) is measured in ppm and coupling constants (J) in Hz. The mass spectra were recorded on a Shimadzu Gas chromatography "GC-2010" instrument mass spectrometer (70 eV) with an electron ionization technique. Elemental micro-analyses were performed on the PerkinElmer CHN-2400 analyzer, and the micro-analyses were within ±0.4% compared to the theoretical values.

# Curves of the new compounds against cancer cell lines in terms of cell viability

| 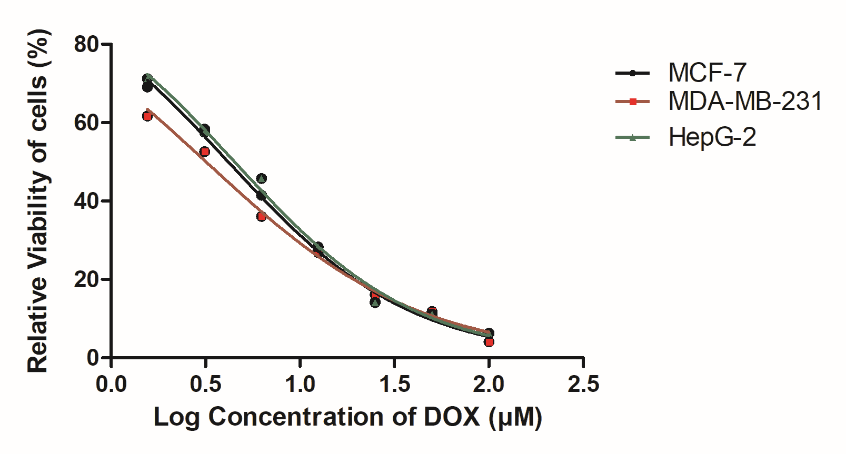 | 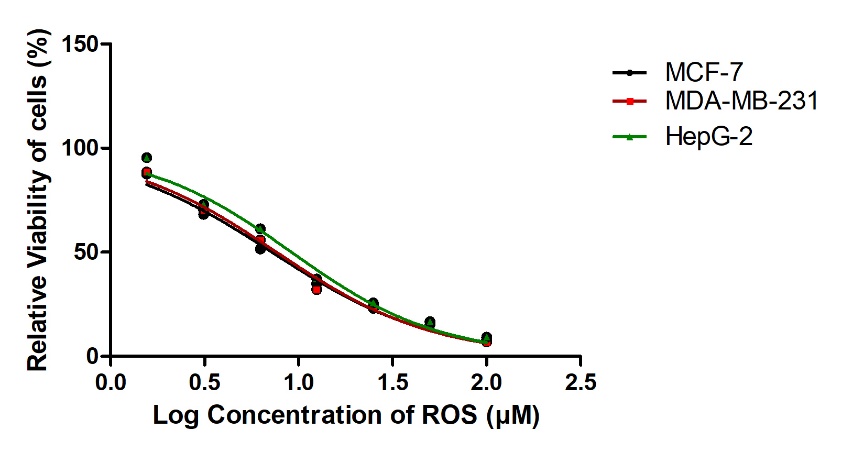 |
| --- | --- |
| 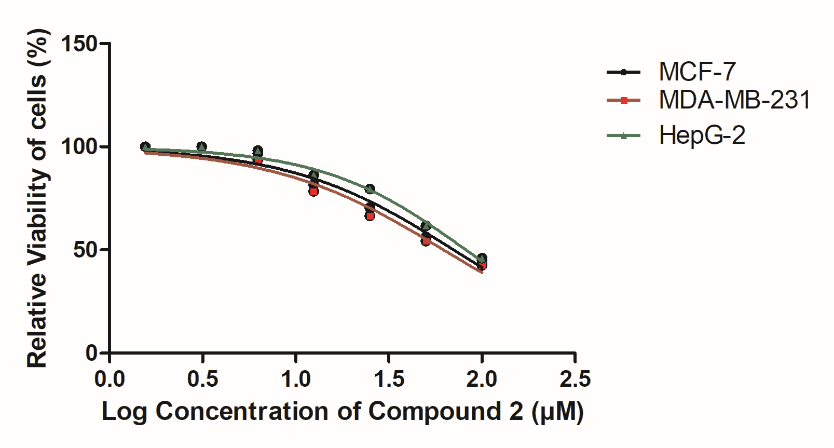 | 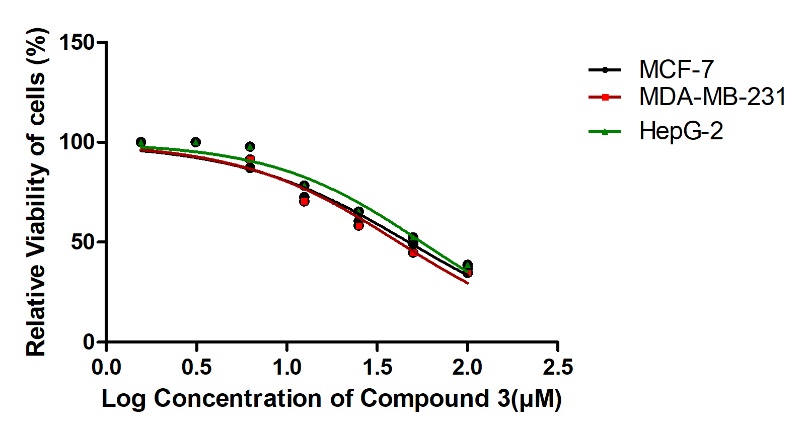 |
| 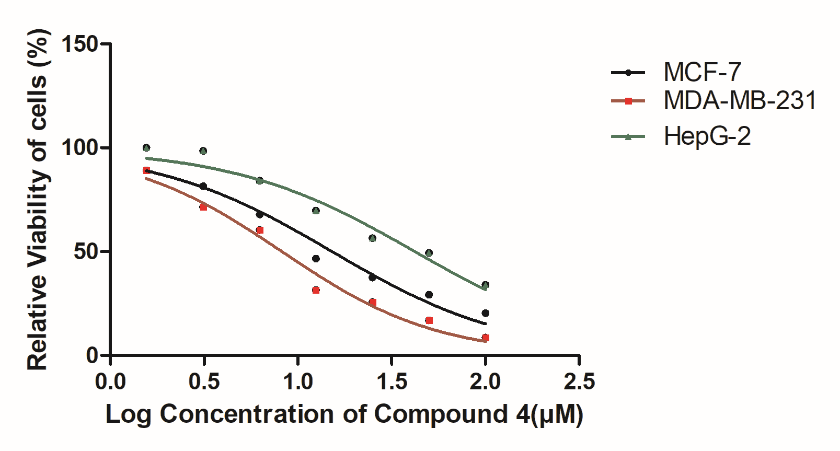 | 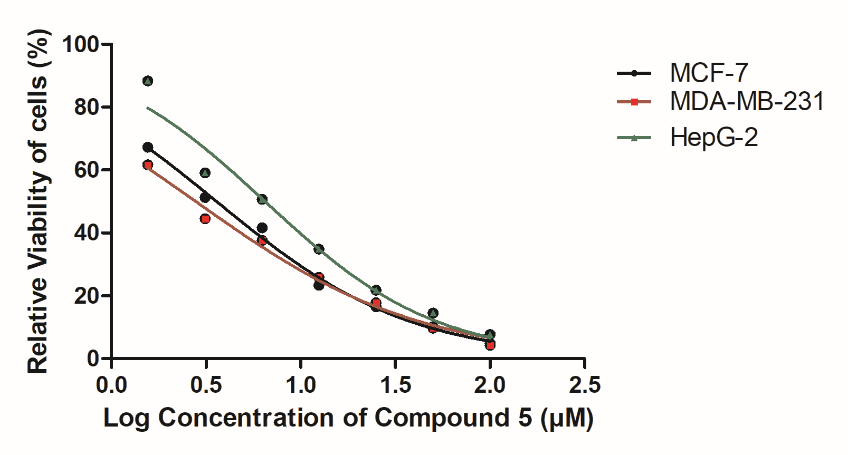 |
| 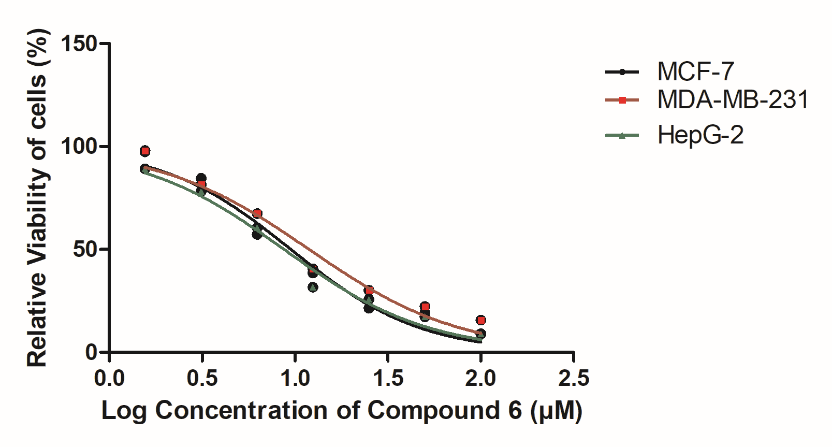 | 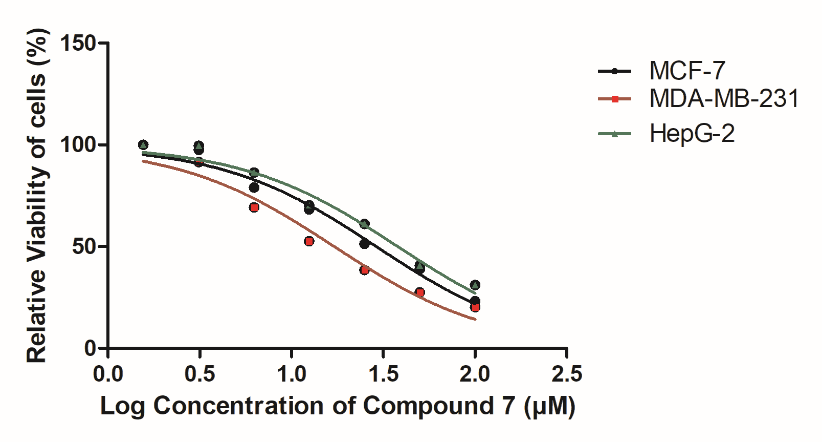 |
| 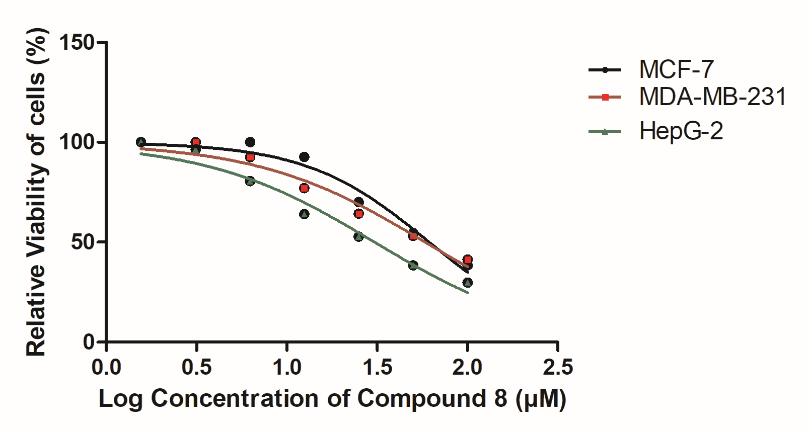 | 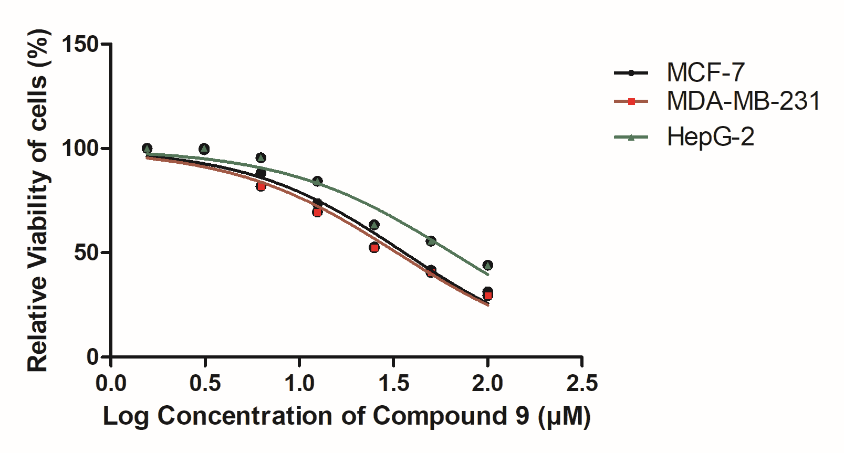 |
| 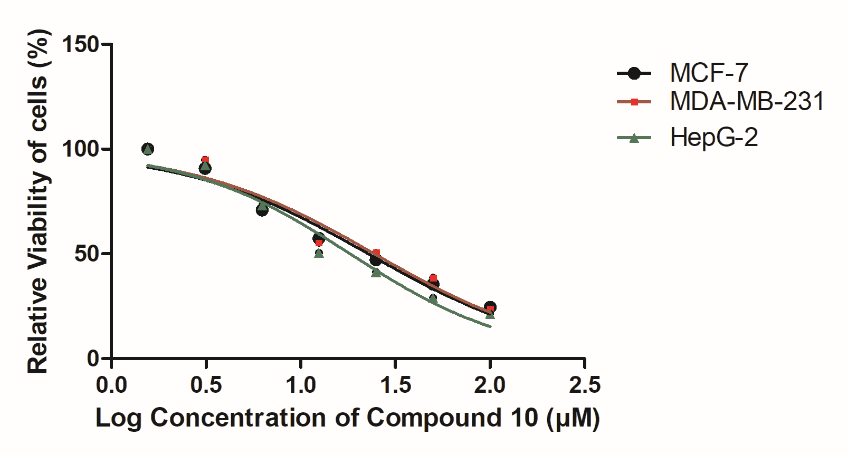 | 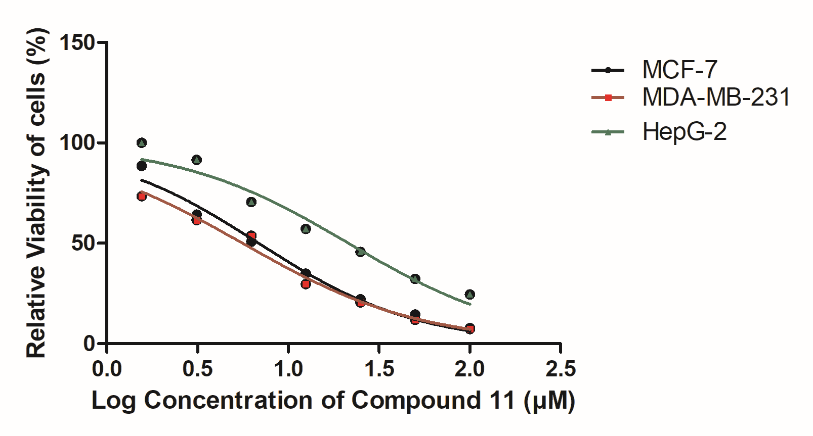 |

**4. Curves of the most active compounds against normal cell line in terms of cells viability**

| 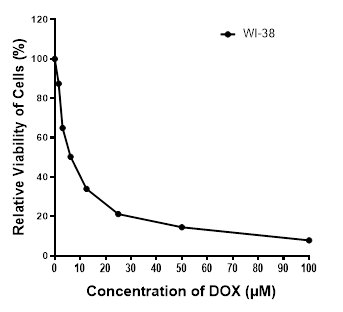 | 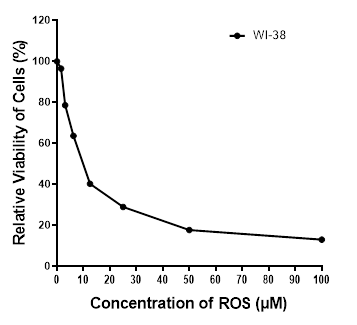 |
| --- | --- |
| 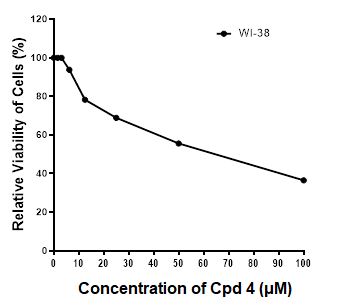 | 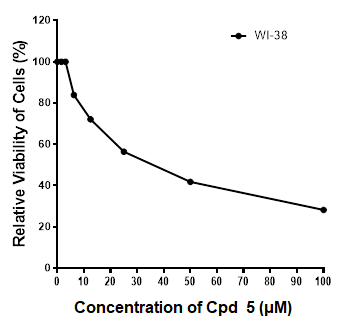 |
| 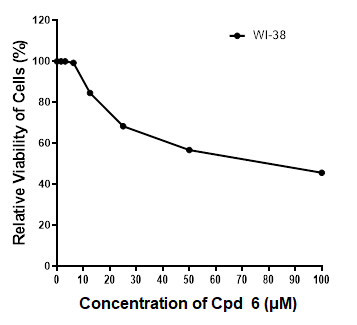 | 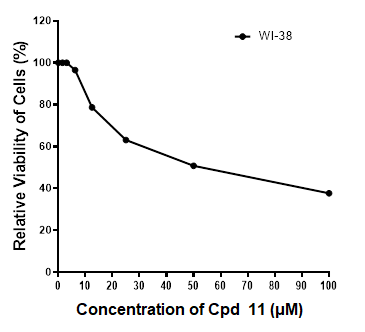 |

# 5. CDK-2 inhibition assay:

| **CDK2** | | | | | | | | | | |  |  |
| --- | --- | --- | --- | --- | --- | --- | --- | --- | --- | --- | --- | --- |
| code | IC50 | conc | log | %inh | T2 | T1 | ∆T | RFU2 | RFU1 | ∆RFU | slope | K.Activity |
| **5** |  | 100 | 2 | 93.8 | 30 | 0 | 30 | 2927 | 0 | 2927 | 1565 | 7.4812 |
| 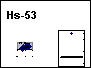 |  | 10 | 1 | 80.2 | 30 | 0 | 30 | 9284 | 0 | 9284 | 1565 | 23.729 |
|  |  | 1 | 0 | 62.4 | 30 | 0 | 30 | 17637 | 0 | 17637 | 1565 | 45.079 |
|  |  | 0.1 | -1 | 45.3 | 30 | 0 | 30 | 25665 | 0 | 25665 | 1565 | 65.597 |
|  |  | 0.01 | -2 | 22.4 | 30 | 0 | 30 | 36424 | 0 | 36424 | 1565 | 93.096 |
| EC |  |  |  | 0 | 30 | 0 | 30 | 46938 | 0 | 46938 | 1565 | 120 |
|  |  |  |  |  |  |  |  |  |  |  |  |  |
| code | IC50 | conc | log | %inh | T2 | T1 | ∆T | RFU2 | RFU1 | ∆RFU | slope | K.Activity |
| **6** |  | 100 | 2 | 92.8 | 30 | 0 | 30 | 3376 | 0 | 3376 | 1565 | 8.6288 |
| 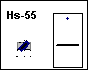 |  | 10 | 1 | 74.8 | 30 | 0 | 30 | 11853 | 0 | 11853 | 1565 | 30.295 |
|  |  | 1 | 0 | 41.7 | 30 | 0 | 30 | 27382 | 0 | 27382 | 1565 | 69.986 |
|  |  | 0.1 | -1 | 31.7 | 30 | 0 | 30 | 32066 | 0 | 32066 | 1565 | 81.958 |
|  |  | 0.01 | -2 | 15.9 | 30 | 0 | 30 | 39472 | 0 | 39472 | 1565 | 100.89 |
| EC |  |  |  | 0 | 30 | 0 | 30 | 46938 | 0 | 46938 | 1565 | 120 |
|  |  |  |  |  |  |  |  |  |  |  |  |  |
| code | IC50 | conc | log | %inh | T2 | T1 | ∆T | RFU2 | RFU1 | ∆RFU | slope | K.Activity |
| **11** |  | 100 | 2 | 95.4 | 30 | 0 | 30 | 2163 | 0 | 2163 | 1565 | 5.5284 |
| 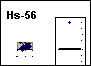 |  | 10 | 1 | 82.7 | 30 | 0 | 30 | 8144 | 0 | 8144 | 1565 | 20.815 |
|  |  | 1 | 0 | 70.6 | 30 | 0 | 30 | 13826 | 0 | 13826 | 1565 | 35.338 |
|  |  | 0.1 | -1 | 51.5 | 30 | 0 | 30 | 22791 | 0 | 22791 | 1565 | 58.252 |
|  |  | 0.01 | -2 | 31.2 | 30 | 0 | 30 | 32286 | 0 | 32286 | 1565 | 82.52 |
| EC |  |  |  | 0 | 30 | 0 | 30 | 46938 | 0 | 46938 | 1565 | 120 |
|  |  |  |  |  |  |  |  |  |  |  |  |  |
| Roscovitine |  | 100 | 2 | 93.9 | 30 | 0 | 30 | 2886 | 0 | 2886 | 1565 | 7.3764 |
| 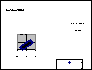 |  | 10 | 1 | 79.7 | 30 | 0 | 30 | 9512 | 0 | 9512 | 1565 | 24.312 |
|  |  | 1 | 0 | 64.2 | 30 | 0 | 30 | 16793 | 0 | 16793 | 1565 | 42.921 |
|  |  | 0.1 | -1 | 38.3 | 30 | 0 | 30 | 28961 | 0 | 28961 | 1565 | 74.022 |
|  |  | 0.01 | -2 | 23.6 | 30 | 0 | 30 | 35882 | 0 | 35882 | 1565 | 91.711 |
| EC |  |  |  | 0 | 30 | 0 | 30 | 46938 | 0 | 46938 | 1565 | 120 |
|  |  |  |  |  |  |  |  |  |  |  |  |  |

#
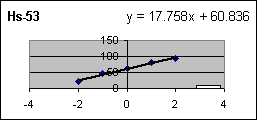

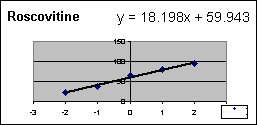


**5**

#

#
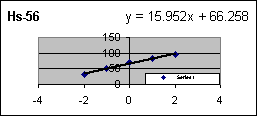

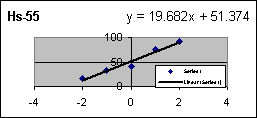


**11**

**6**

# 6. Molecular docking study:

The 2D structures of tetrahydrobenzo[*b*]thiophene carboxamides **5**, **6**, and **11** were drawn through Chem. Draw. The protonated 3D was employed using standard bond lengths and angles, using the Molecular Operating Environment (MOE-Dock) software version 2024.0601. Then, the geometry optimization and energy minimization were applied to get the Conf Search module in MOE, followed by saving of the moe file for upcoming docking process. The co-crystallized structure of CDK-2/cyclin A2 with its natural ligand, roscovitine (PDB code: 3DDQ) from the protein data bank. All minimizations were performed using MOE until an RMSD gradient of 0.05 kcal∙mol^−1^Å^−1^ with MMFF94x force field and the partial charges were automatically calculated. Preparation of the enzyme’s structure was done for molecular docking using the Protonate 3D protocol with the default options in MOE. London dG scoring function and Triangle Matcher placement method were used in the docking protocol. Initially, the validation of the docking process was established by docking the native ligand, followed by docking the derivatives **5**, **6**, and **11** within the ATP-binding sites after eliminating the co-crystallized ligand.

**7. HPLC analysis for compounds 5, 6, and 11:**

HPLC analysis was performed using a Shimadzu HPLC system controlled by LabSolutions software equipped with a UV/PDA detector. The synthesized tetrahydrobenzo[*b*]thiophene purity was determined using high-performance liquid chromatography (HPLC). Samples were initially dissolved in DMSO and subsequently diluted HPLC-grade methanol and filtered through a 0.45 µm membrane filter before injection. Analyses were carried out on an HPLC system equipped with a UV detector. Analyses Separation was achieved using a C18 reversed-phase column (250 × 4.6 mm, 5 µm particle size). The mobile phase consisted of acetonitrile and water (7:3, v/v) delivered in isocratic mode at a flow rate of 1.0 mL/min. The detection wavelength was set at 254 nm, and the injection volume was 20 µL. The column temperature was maintained at 25 °C.


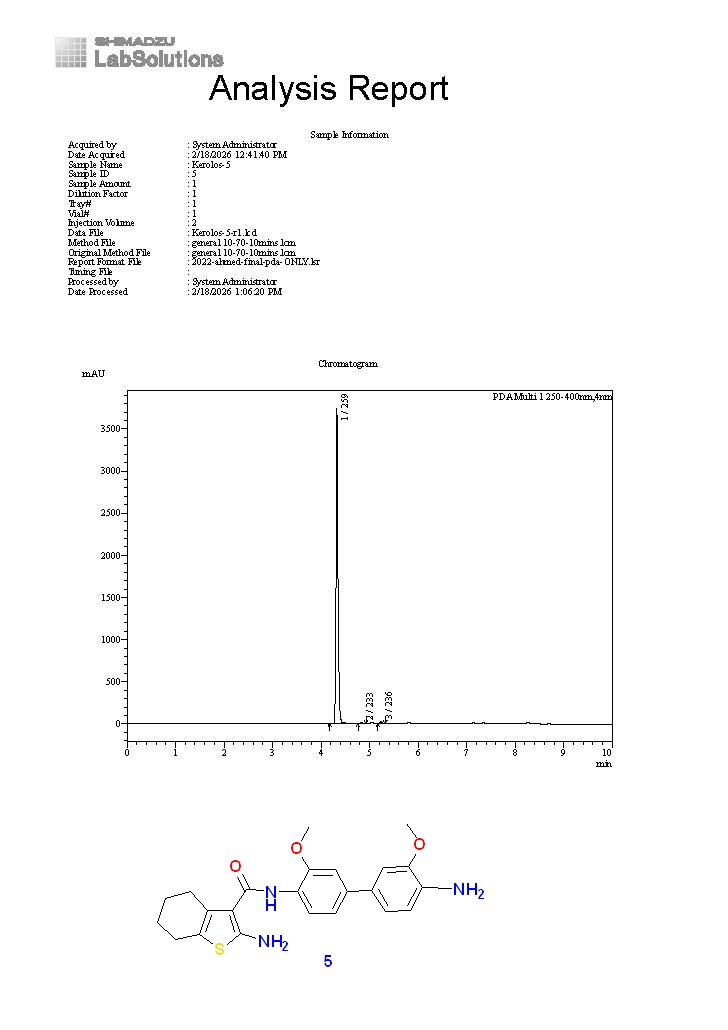


#
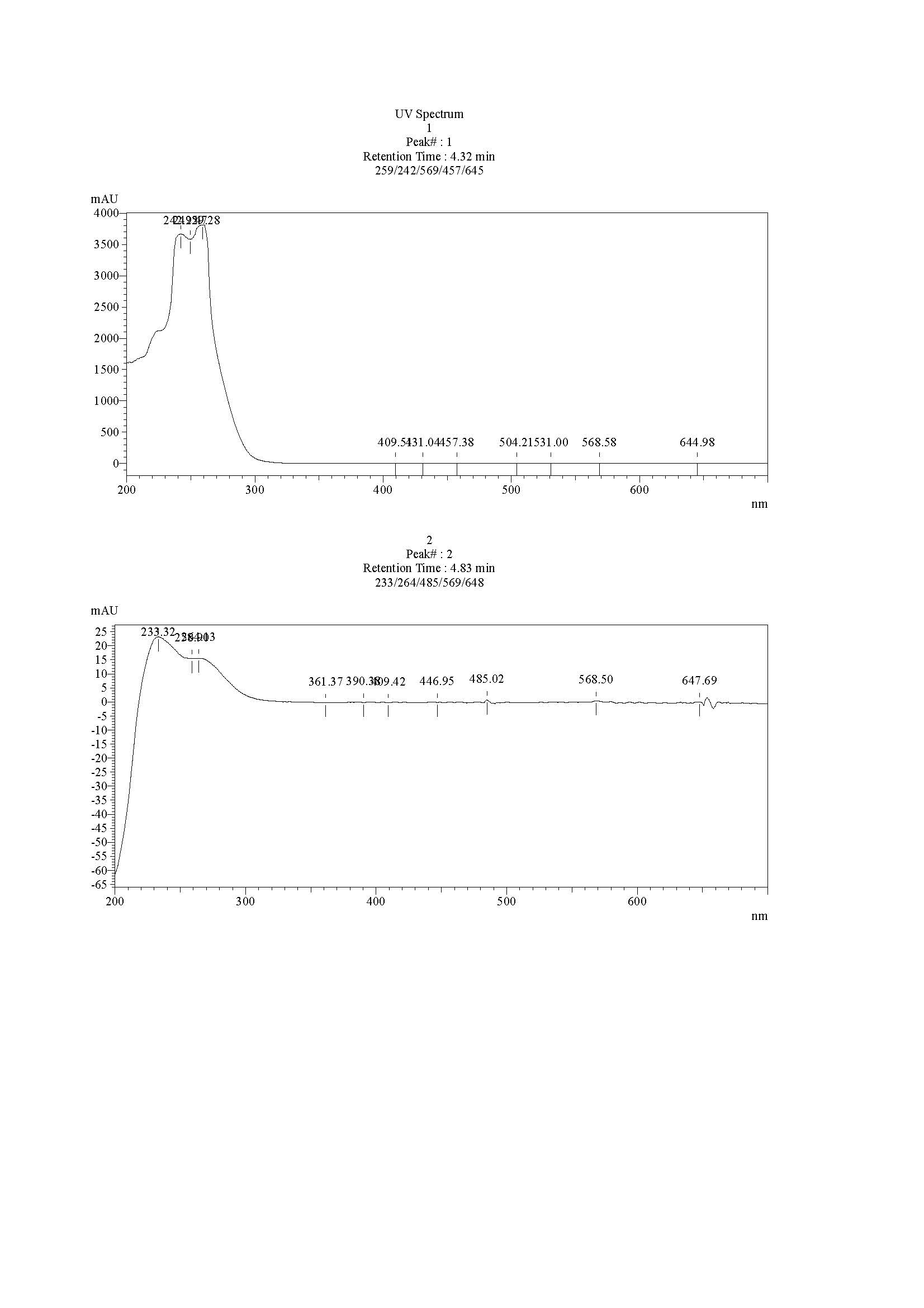


#
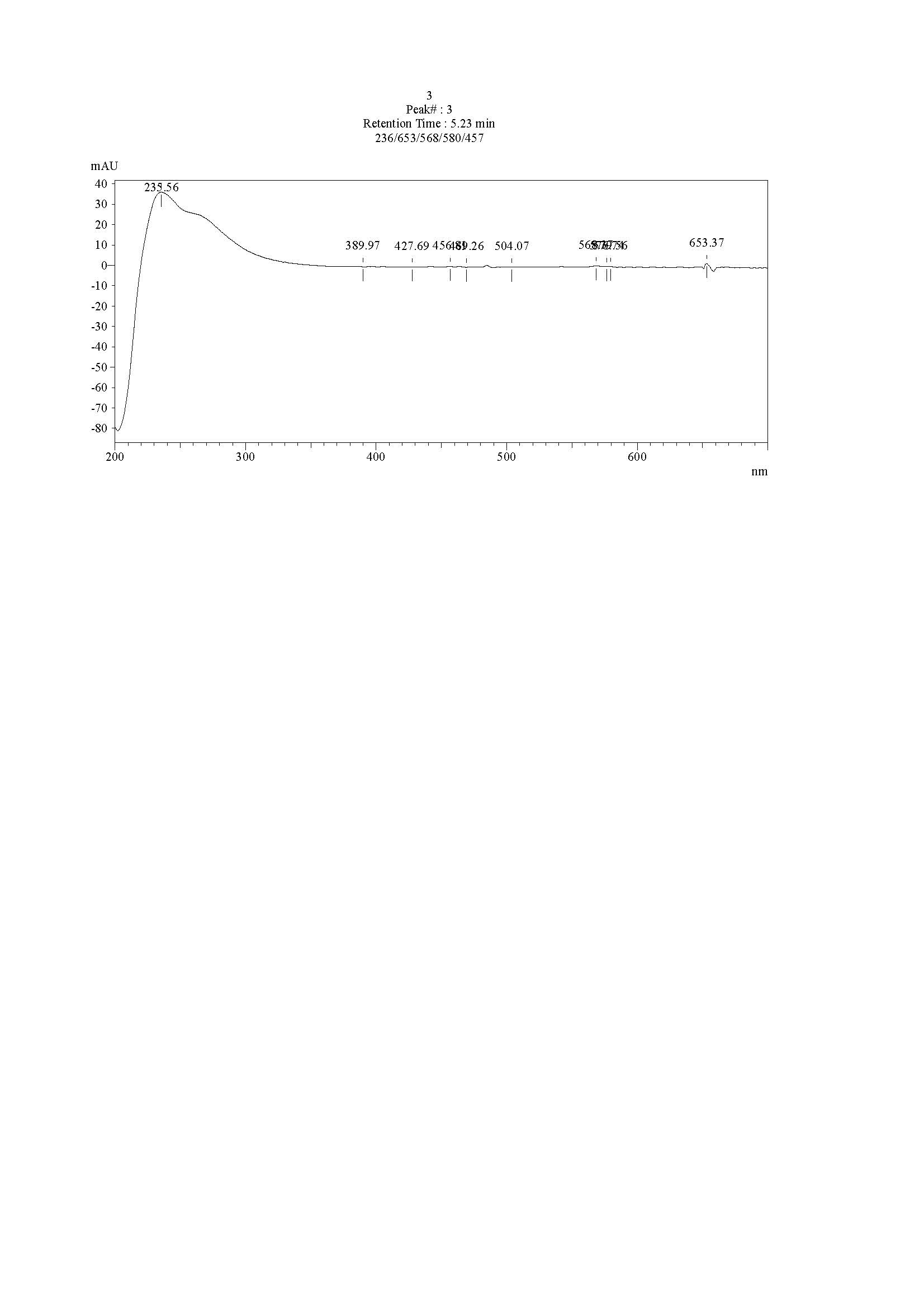


#
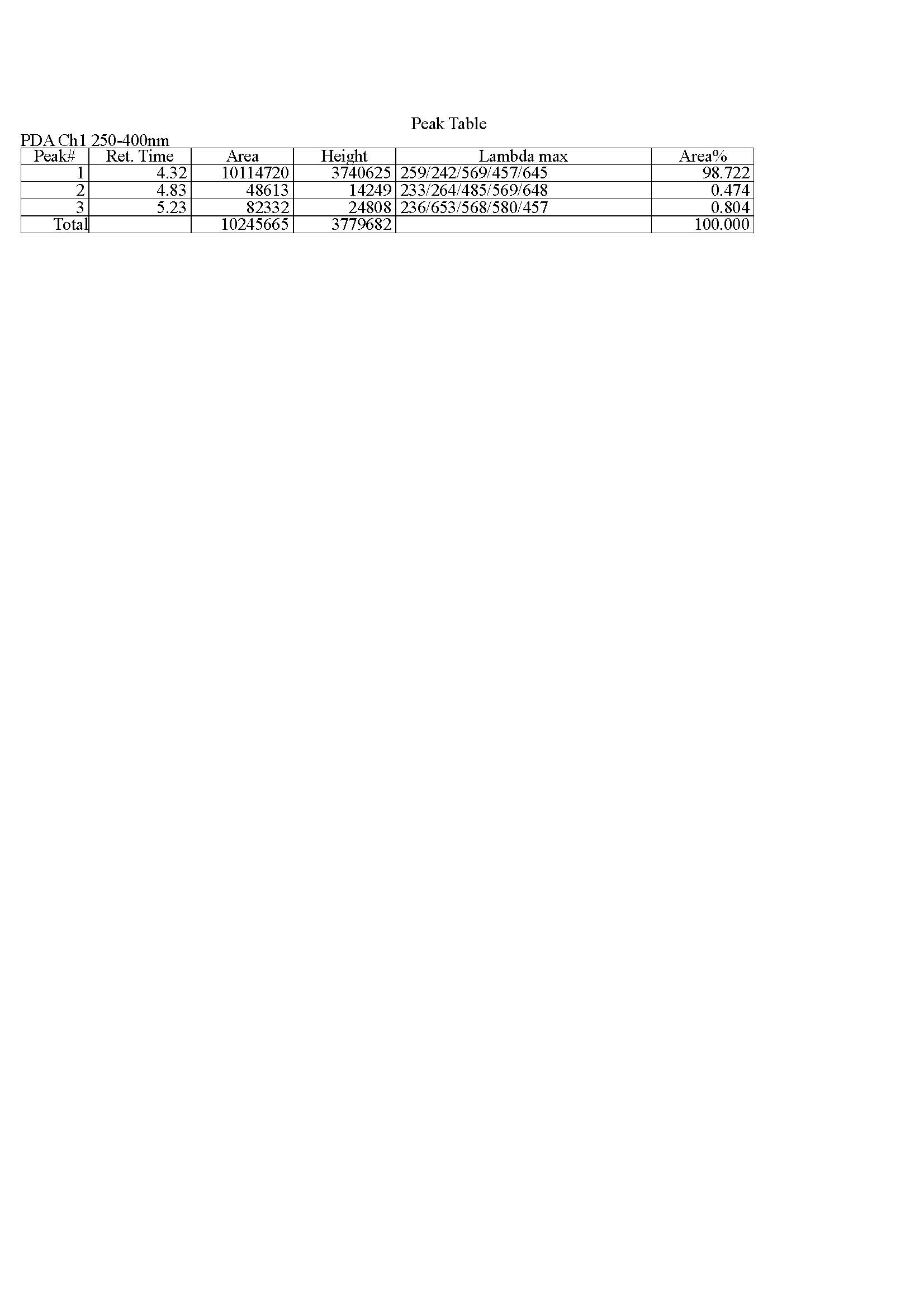


#
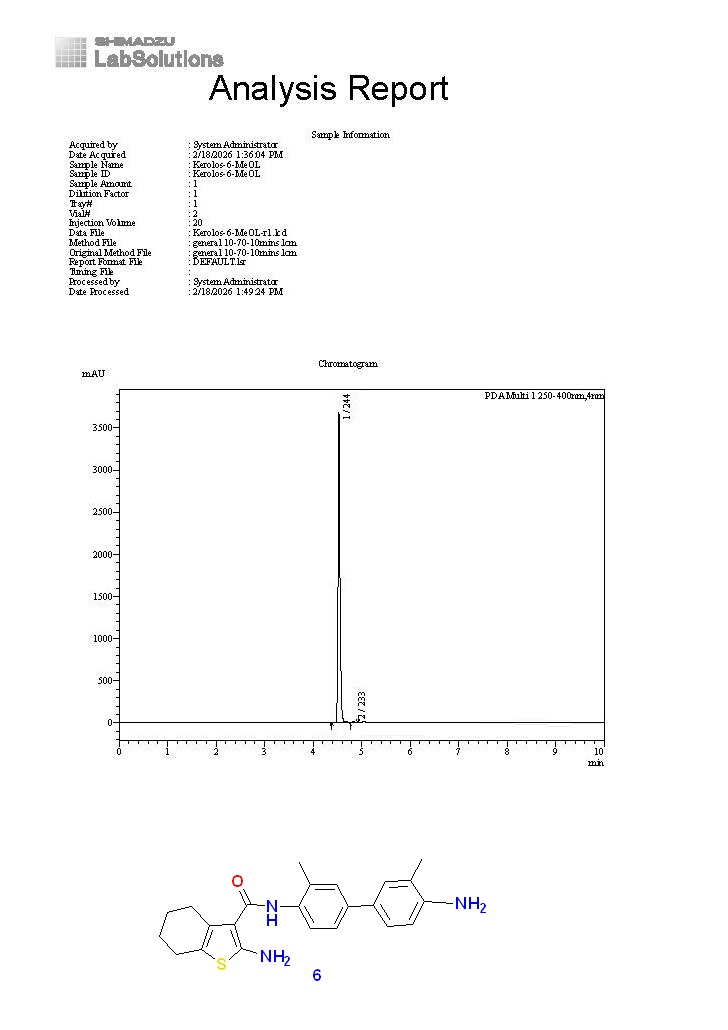


#
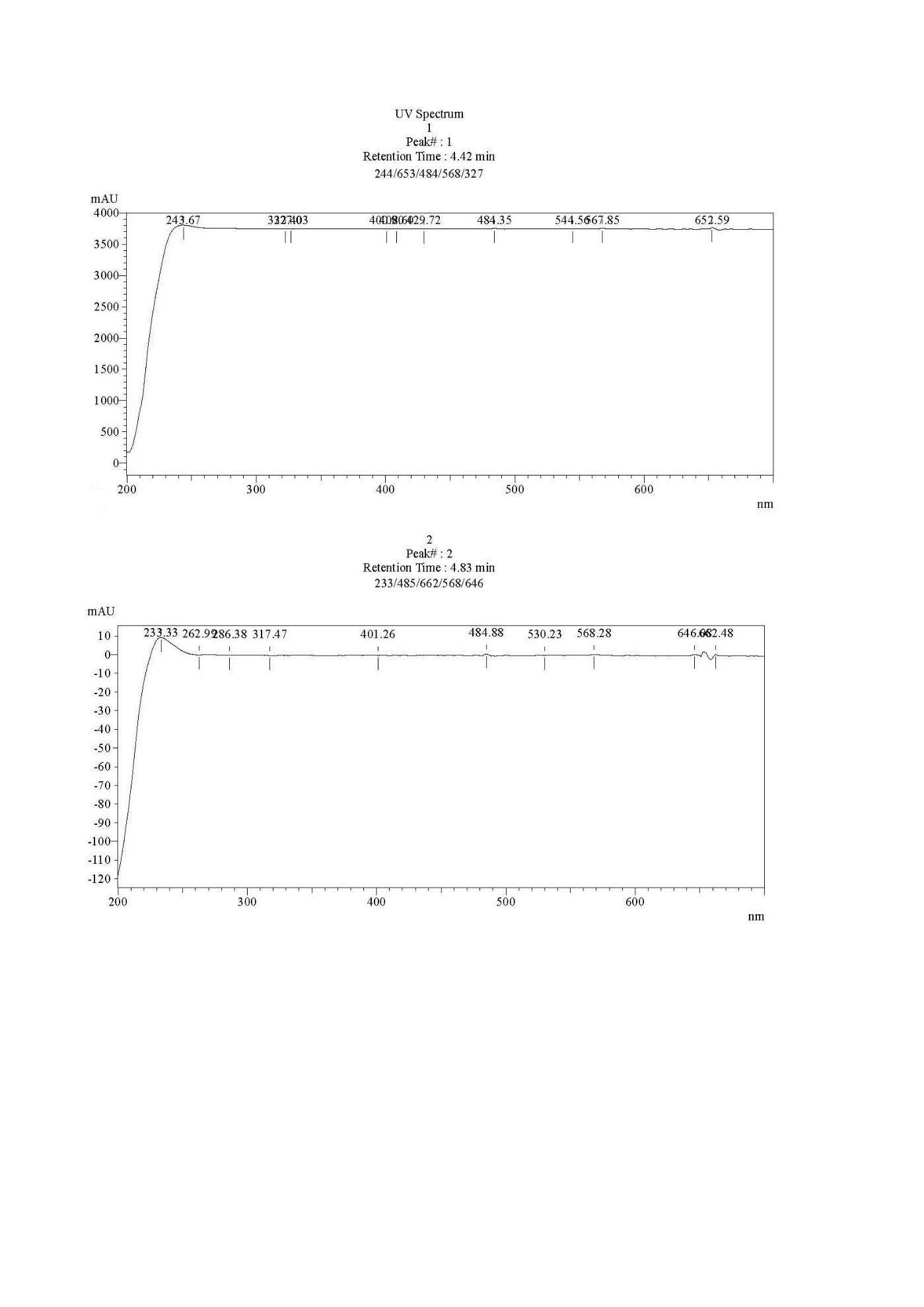


#
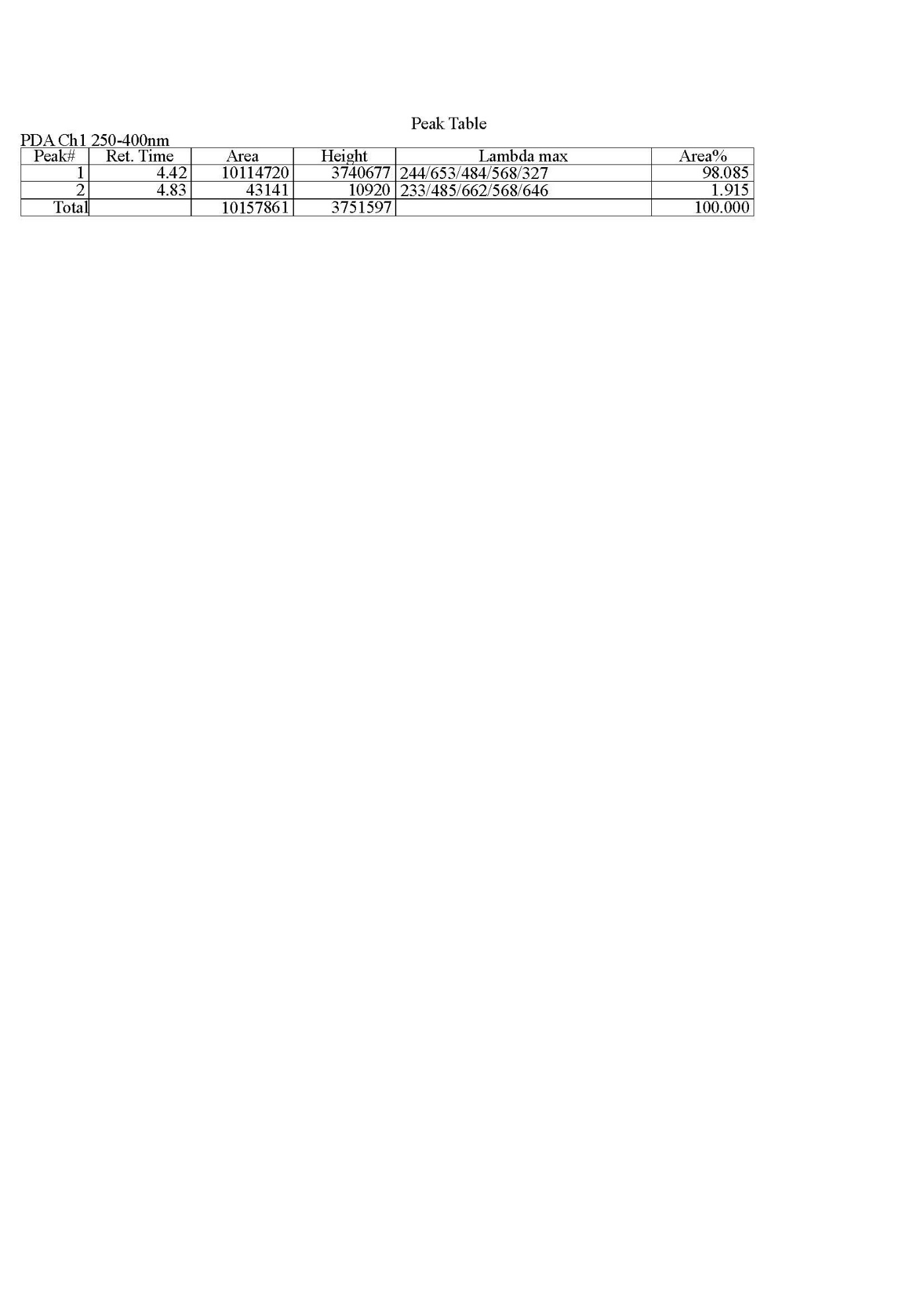


#
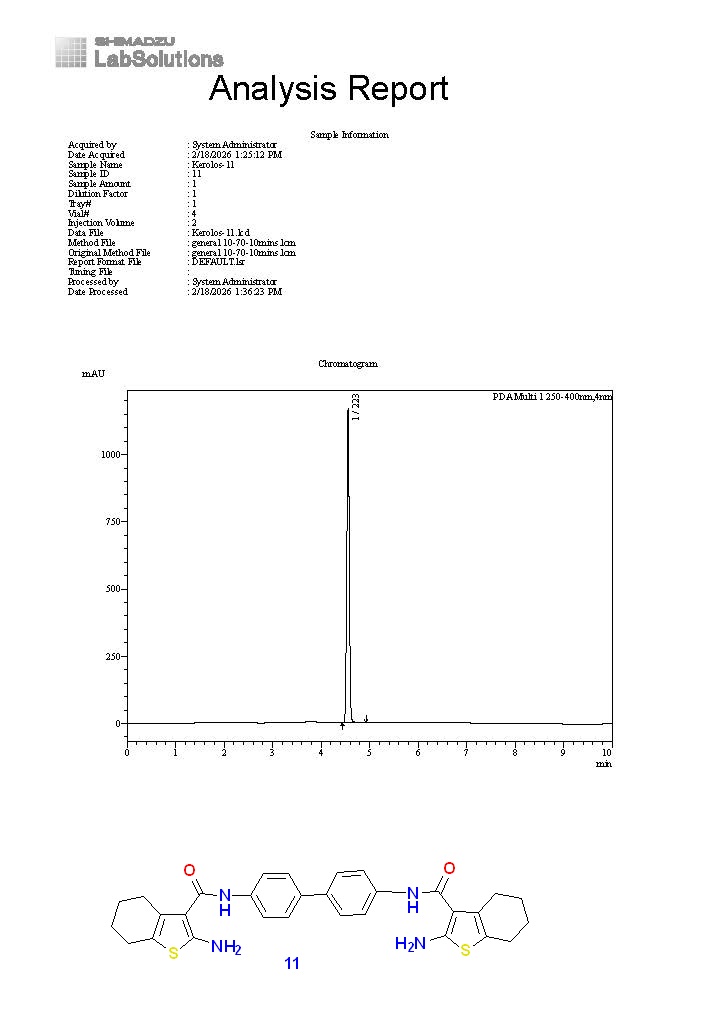


#
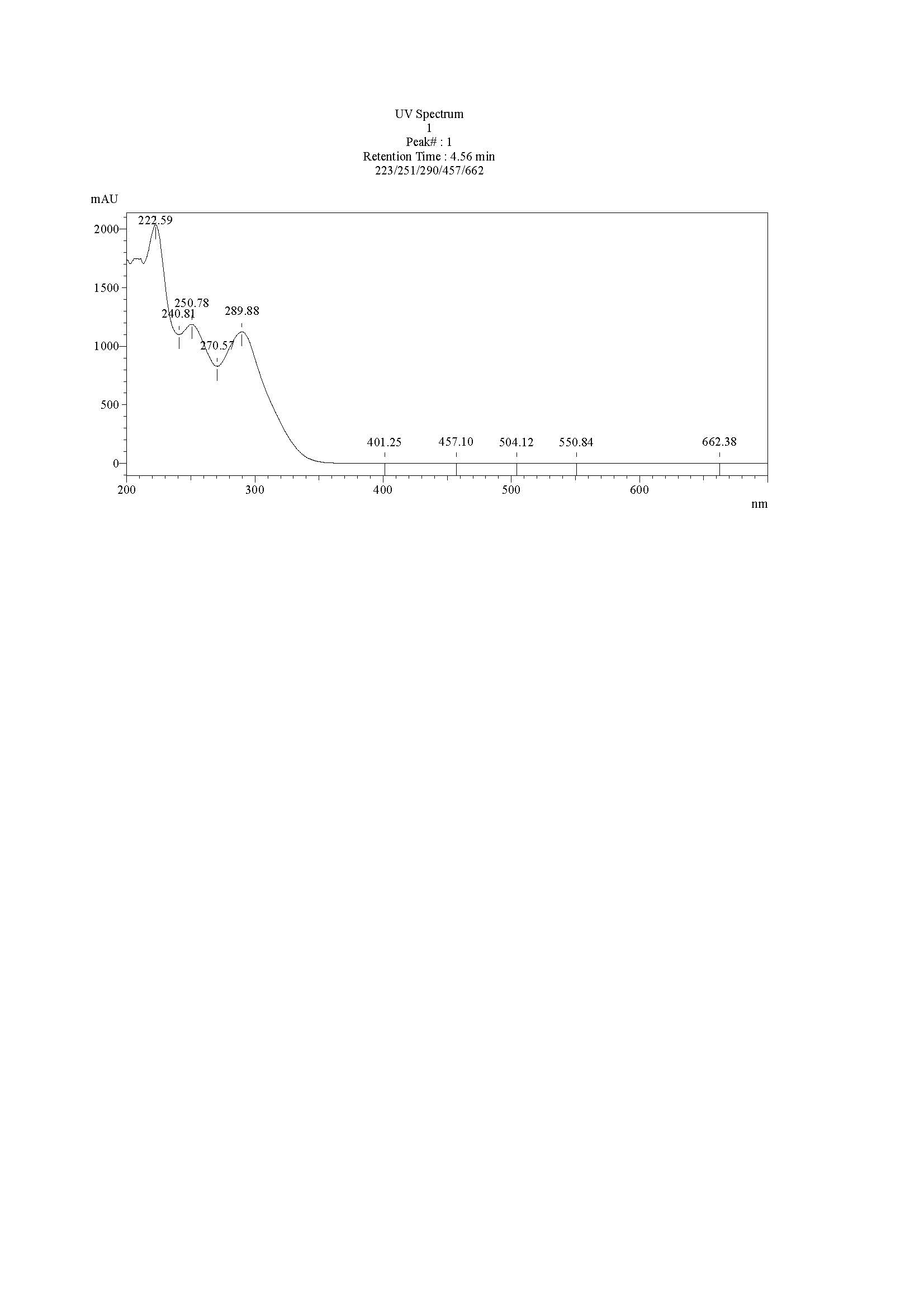


#
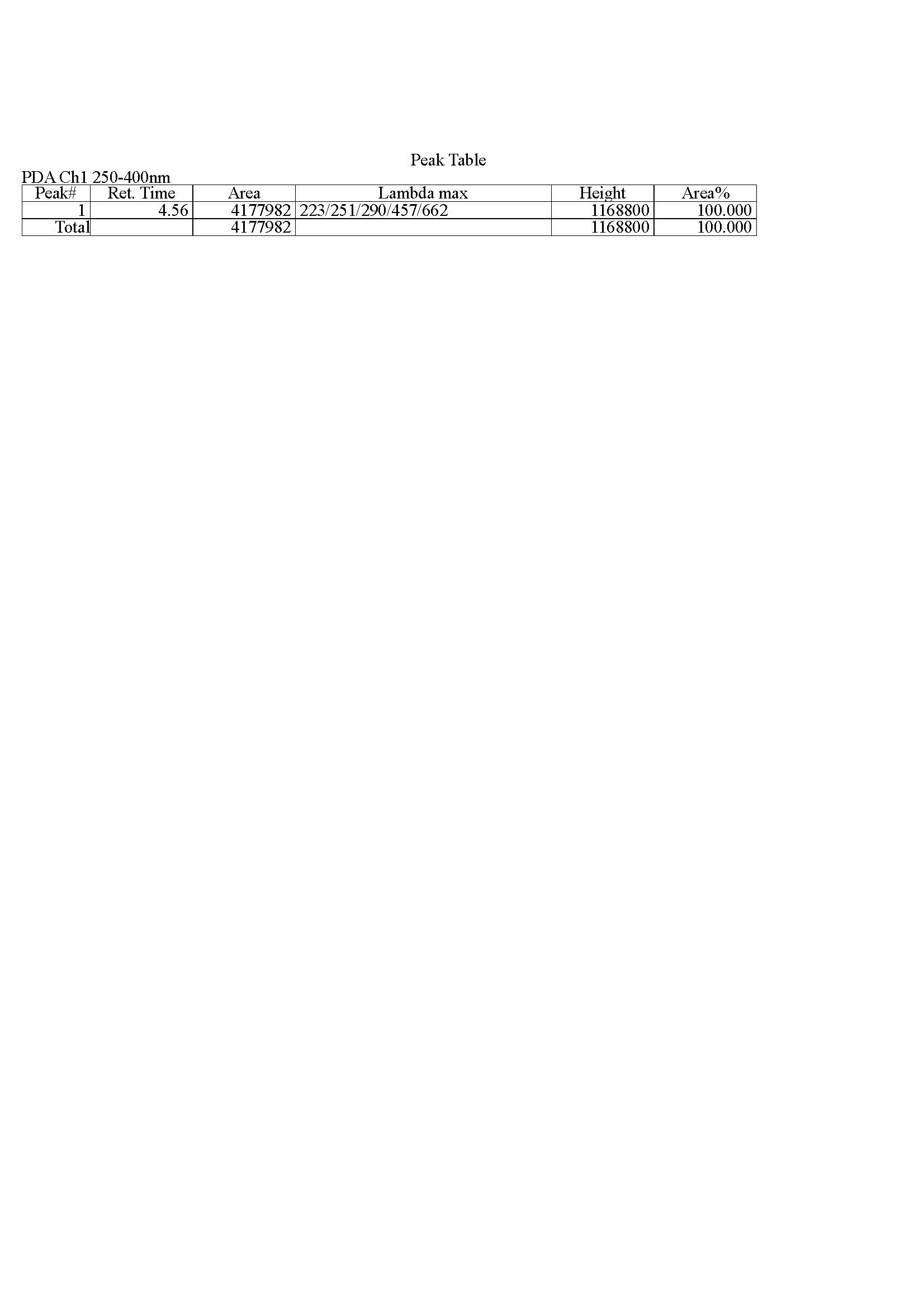


1. **CDK-2 inhibition**

The *in vitro* CDK2 Kinase assay was carried out for the target compounds using CDK2 luminescence kinase assay (Biosciences, SD, USA). Protocol steps involve diluting the enzyme and the substrate, 1 µL of ATP (500 µM) and the inhibitors in Kinase Buffer before the addition of 1 µL of inhibitor, 2 µL of the enzyme and 2 µL of the substrate/ATP mix. After 10 min incubation at room temperature, 5 µL of ADP-Glo™ Reagent was added then incubated for 40 min. Afterwards, an amount of Kinase Detection Reagent (10 µL) was added and incubated for 30 min at room temperature. For the control, add the same solution without inhibitor. Luminescence was then detected and the luminescent signal was directly correlated with the quantity of ATP present but inversely correlated with the kinase activity. The IC_50_ value was interpolated from dose response data with five different concentrations for each tested compound and ±SD value was recorded from triplicate experiment.

1. **Annexin-V-FITC apoptosis assay**

Apoptosis was estimated by staining the cells with Annexin V fluorescein isothiocyanate (FITC) and counterstaining with PI using the Annexin V-FITC/PI apoptosis detection kit (BD Biosciences, San Diego, CA) according to the manufacturer's instructions. Briefly, 4 × 10^6^ cell/T 75 flask were exposed to the examined compounds at three concentrations (IC_50_ (x), 5x and 10x) for 24 h. The cells then were collected by trypsinization and 0.5 × 10^6^ cells were washed twice with PBS and stained with 5 µL Annexin V-FITC and 5 µL PI in 1 × binding buffer for 15 min at room temperature in the dark. Analyses were performed using FACS Caliber flow cytometer (BD Biosciences, San Jose, CA). The data are expressed as mean±SD of three independent experiments.

**Table S1**: Apoptosis assay of compound **11**

| Compound | **Apoptotic cells (%)** | | | **Necrotic cells (%)** |
| --- | --- | --- | --- | --- |
|  | Total | Early | Late |  |
| compound **4** | 22.91±1.51 | 6.49±0.28 | 13.54±0.63 | 2.88±0.08 |
| Control | 2.42±0.07 | 0.54±0.03 | 0.21±0.01 | 1.67±0.04 |

1. **Cell cycle analysis**

The cells were treated with the selected compounds at three concentrations (IC_50_ (x), 5x and 10x) and at concentration 10x for 24 h. After treatment, the cells were washed twice with ice-cold phosphate buffer saline (PBS), collected by centrifugation, and fixed in ice-cold 70% (v/v) ethanol, washed with PBS, re-suspended with 0.1 mg/mL RNase, stained with 40 mg/mL propidium iodide (PI), and analyzed by flow cytometry using FACSCalibur (Becton Dickinson). The cell cycle distributions were calculated using Cell-Quest software (Becton Dickinson). The data are expressed as mean±SD of three independent experiments.

**Table S1.** Statistical fitting metrics and precision analysis for the cytotoxic activity of the synthesized compounds

| **Analog number** | **MCF-7** | | **MDA-MB-231** | | **HepG-2** | |
| --- | --- | --- | --- | --- | --- | --- |
|  | **95% CI (µM)** | **R^2^** | **95% CI (µM)** | **R^2^** | **95% CI (µM)** | **R^2^** |
| **2** | 62.36-80.26 | 0.97 | 54.82-71.05 | 0. 97 | 76.11-88.92 | 0.99 |
| **3** | 41.33-54.14 | 0.97 | 36.01-48.06 | 0. 96 | 48.91-63.59 | 0.97 |
| **4** | 13.00-17.94 | 0.95 | 7.44-9.14 | 0.98 | 36.47-48.82 | 0.96 |
| **5** | 3.40-3.80 | 0.99 | 2.58-3.03 | 0.99 | 5.74-7.35 | 0.97 |
| **6** | 8.41-10.74 | 0.97 | 10.41-13.67 | 0.97 | 7.89-9.69 | 0.98 |
| **7** | 26.22-32.19 | 0.98 | 14.91-19.75 | 0.97 | 33.99-42.58 | 0.98 |
| **8** | 55.51-67.94 | 0.97 | 51.03-66.81 | 0.97 | 26.86-34.93 | 0.97 |
| **9** | 32.00-40.92 | 0.97 | 28.99-37.62 | 0.97 | 55.51-75.87 | 0.95 |
| **10** | 19.72-26.37 | 0.96 | 20.55-29.41 | 0.94 | 15.70-21.30 | 0.96 |
| **11** | 6.28-7.54 | 0.98 | 5.16-6.11 | 0.99 | 18.63-24.85 | 0.96 |
| **Doxorubicin** | 3.99-4.37 | 0.99 | 2.99-3.39 | 0.99 | 4.26-4.773 | 0.99 |
| **Roscovitine** | 6.74-7.74 | 0.99 | 7.03-8.33 | 0.99 | 8.31-10.16 | 0.98 |
